# Supplementary material for: The development of children born to young mothers with no, first- or second-generation HIV acquisition in the Eastern Cape province, South Africa: a cross-sectional study
Source: BMJ Open. 2022 Oct 13;12(10):e058340. doi: 10.1136/bmjopen-2021-058340 (PMC9562751; doi:10.1136/bmjopen-2021-058340)
Supplement: Supplementary data [file bmjopen-2021-058340supp001.pdf]

Appendix 1

Infant (0-68 month) developmental outcomes, according to familial patterns of HIV infection – sample limited to adolescent mothers only

	Overall (*N*=1015)	Third Generation (*n*= 23)	Second Generation (*n*= 229)	No HIV Exposure (*n*= 721)	p-value
**Mullen Scales of Child Development** *(M, SD)*					
Gross Motor Score*	49.7 (12.5)	54.3 (16. 7)[a]	46.8 (12.7)[b]	50.3 (12.2)	<.001
Visual Reception Score	42.1 (14.2)	44.0 (13.7)	40.4 (14.1)	42.5 (14.2)	.12
Fine Motor Score	43.7(14.7)	45.0 (16.2)	41.3 (15.5)[b]	44.4 (14.3)	.017
Receptive Language Score	47.5 (13.5)	48.5 (14.0)	46.4 (14.9)	47.8 (13.0)	.37
Expressive Language Score	51.6 (13.5)	51.1 (14.0)	51.0 (14.8)	51.8 (13.0)	.71
Composite Score	93.3 (21.3)	95.0 (23.1)	90.6 (22.8)	94.1 (20.7)	.098
**WHO Disability Score- Any Disability** *(N, %)*	234 (26.9%)	6 (27.3%)	63 (28.9%)	165 (26.2%)	.73
**Any Days Sick** (past month) *(N, %)*	212 (22.1%)	3 (15.0%)	37 (16.2%)	172 (24.2%)	.032

Notes. Group comparisons were conducted using χ^2 tests for categorical variables and univariate analyses of variance for continuous variables. *Gross motor score: $n = 831$. Mullen subdomain scores range from 20-80, composite scores from 49-155. Tukey's range post hoc tests were undertaken to identify mean differences between groups for continuous variable: [a] Statistically different from the second generation ($p<0.05$), [b] Statistically different from HIV unexposed group ($p<0.05$).
