# Supplementary material for: The development of children born to young mothers with no, first- or second-generation HIV acquisition in the Eastern Cape province, South Africa: a cross-sectional study
Source: BMJ Open. 2022 Oct 13;12(10):e058340. doi: 10.1136/bmjopen-2021-058340 (PMC9562751; doi:10.1136/bmjopen-2021-058340)
Supplement: Author's manuscript [file bmjopen-2021-058340.draft_revisions.pdf]

BMJ Open

BMJ Open is committed to open peer review. As part of this commitment we make the peer review history of every article we publish publicly available.

When an article is published we post the peer reviewers' comments and the authors' responses online. We also post the versions of the paper that were used during peer review. These are the versions that the peer review comments apply to.

The versions of the paper that follow are the versions that were submitted during the peer review process. They are not the versions of record or the final published versions. They should not be cited or distributed as the published version of this manuscript.

BMJ Open is an open access journal and the full, final, typeset and author-corrected version of record of the manuscript is available on our site with no access controls, subscription charges or pay-per-view fees (http://bmjopen.bmj.com).

If you have any questions on BMJ Open's open peer review process please email info.bmjopen@bmj.com

BMJ Open

THE CHILDREN OF YOUNG MOTHERS BORN WITH HIV – A study of the cognitive development of infants who are the Third Generation affected by HIV

Journal:	*BMJ Open*
Manuscript ID	bmjopen-2021-058340
Article Type:	Original research
Date Submitted by the Author:	20-Oct-2021

Complete List of Authors:	Sherr, Lorraine; University College London<br>Haag, Katharina; UCL, Institute for Global Health<br>Roberts, Kathryn; UCL, Institute for Global Health<br>Cluver, Lucie; University of Oxford, Centre for Evidence-Based Social Intervention; University of Cape Town, Department of Psychiatry and Mental Health<br>Wittesaele, Camille; University of Oxford, Department of Social Policy & Intervention; London School of Hygiene & Tropical Medicine, Department of Infectious Disease Epidemiology<br>Saliwe, Bongiwe; University of Cape Town, Centre for Social Science Research<br>Tolmay, Janke; University of Cape Town, Centre for Social Science Research<br>Langwenya, Nontokozo; University of Cape Town, Centre for Social Science<br>Jochim, Janina; University of Oxford, Department of Social Policy & Intervention<br>Saal, Wylene; University of Cape Town, Centre for Social Science Research<br>Zhou, Siyanai; University of Cape Town, Centre for Social Science Research<br>Chen, Jenny; University of Oxford, Department of Social Policy & Intervention<br>Toska, Elona; University of Cape Town Faculty of Humanities, Centre for Social Science Research & Department of Sociology; University of Oxford, Social Policy and Intervention

Keywords:	HIV & AIDS < INFECTIOUS DISEASES, Community child health < PAEDIATRICS, EPIDEMIOLOGY, Health policy < HEALTH SERVICES ADMINISTRATION & MANAGEMENT, Developmental neurology & neurodisability < PAEDIATRICS

SCHOLARONE™
Manuscripts

I, the Submitting Author has the right to grant and does grant on behalf of all authors of the Work (as defined in the below author licence), an exclusive licence and/or a non-exclusive licence for contributions from authors who are: i) UK Crown employees; ii) where BMJ has agreed a CC-BY licence shall apply, and/or iii) in accordance with the terms applicable for US Federal Government officers or employees acting as part of their official duties; on a worldwide, perpetual, irrevocable, royalty-free basis to BMJ Publishing Group Ltd ("BMJ") its licensees and where the relevant Journal is co-owned by BMJ to the co-owners of the Journal, to publish the Work in this journal and any other BMJ products and to exploit all rights, as set out in our licence.

The Submitting Author accepts and understands that any supply made under these terms is made by BMJ to the Submitting Author unless you are acting as an employee on behalf of your employer or a postgraduate student of an affiliated institution which is paying any applicable article publishing charge ("APC") for Open Access articles. Where the Submitting Author wishes to make the Work available on an Open Access basis (and intends to pay the relevant APC), the terms of reuse of such Open Access shall be governed by a Creative Commons licence – details of these licences and which Creative Commons licence will apply to this Work are set out in our licence referred to above.

Other than as permitted in any relevant BMJ Author's Self Archiving Policies, I confirm this Work has not been accepted for publication elsewhere, is not being considered for publication elsewhere and does not duplicate material already published. I confirm all authors consent to publication of this Work and authorise the granting of this licence.

For peer review only

THE CHILDREN OF YOUNG MOTHERS BORN WITH HIV – A study of the cognitive development of infants who are the Third Generation affected by HIV

Authors: Lorraine Sherr[1], Katharina Haag[1], Kathryn Roberts[1], Lucie Cluver[2,3], Camille Wittesaele2,[4], Bongiwe Saliwe[5], Janke Tolmay[5], Nontokozo Langwenya[5], Janina Jochim[2], Wylene Saal[5], Siyanai Zhou[5], Jenny Chen[2], Elona Toska[2,5,6]

1 Institute for Global Health, University College London, UK

2 Department of Social Policy & Intervention, University of Oxford, Oxford, UK

3 Department of Psychiatry and Mental Health, University of Cape Town, Cape Town, South Africa

4 Department of Infectious Disease Epidemiology, London School of Hygiene & Tropical Medicine, London, UK

5 Centre for Social Science Research, University of Cape Town, Cape Town, South Africa

6 Department of Sociology, University of Cape Town, Cape Town, South Africa

Corresponding author:

Professor Lorraine Sherr (l.sherr@ucl.ac.uk), University College London, Department of Global Health, Royal Free Hospital Campus, Rowland Hill St, London, NW3 2PF

Tel: +442080168071, ext 68071

Fax: +44 207 794 1224

Word Count: 4630 (excluding title page, abstract, references, figures ad tables)

For peer review only - http://bmjopen.bmj.com/site/about/guidelines.xhtml

ABSTRACT (299 words)

Objectives: Long-term, inter-generational effects of HIV require investigation. We compared developmental outcomes of the "third generation" (i.e., children of perinatally infected mothers), the "second generation" (i.e., with recently infected mothers) and children of mothers not living with HIV.

Design, Setting and Participants: A cross-sectional community sample of *N*=1015 young mothers (ages 12-25) and their first children, from South Africa's Eastern Cape Province.

Outcome Measures: Child scores on the Mullen Scales of Early Learning (MSEL), the WHO Ten Questions Screen for Disability and maternal demographics were compared between groups using $\chi2$-tests and univariate ANOVAs. Hierarchical linear regressions investigated predictive effects of familial HIV infection patterns on child MSEL composite scores, controlling for demographic and family environment variables.

Results: 71.3% (*n*=724) of children were born to mothers not living with HIV; 2.7% (*n*=27; 1 living with HIV) were third and 26.0% (*n*=264; 11 living with HIV) second generation children. Second generation children performed poorer on gross (*M*=47.0, *SD*=13.1) and fine motor functioning (*M*=41.4, *SD*=15.2) and the MSEL composite score (*M*=90.6, *SD*=23.0) than children with non-infected mothers (gross motor: *M*=50.4, *SD*=12.3; fine motor: *M*=44.4, *SD*=14.1; composite score: *M*=94.1, *SD*=20.7). Predictive effects for the composite score were diminished when maternal risk factors were controlled for (p=.059). The third generation performed at similar levels to non-exposed children (gross motor: *M*=52.4, *SD*=16.1; fine motor: *M*=44.3, *SD*=16.1, composite score: *M*=94.7, *SD*=22.2), though analyses were underpowered for definite conclusions. No group differences were found for disability rates ($\chi^2 = .02$, $p = .90$).

Conclusion: Longstanding familial HIV infection may facilitate care pathways and coping, explaining similar cognitive development among not exposed and third generation children.

For peer review only - http://bmjopen.bmj.com/site/about/guidelines.xhtml

Recently infected mothers and their children may struggle due to the disruptiveness of new HIV diagnoses and incomplete access to care/ support during pregnancy and early motherhood. Targeted intervention and fast-tracking into services may improve maternal mental health and socioeconomic support.

Keywords: HIV; adolescent mothers, infants, cognitive development, mental health, environmental factors

For peer review only

For peer review only - http://bmjopen.bmj.com/site/about/guidelines.xhtml

STRENGTHS AND LIMITATIONS OF THIS STUDY

- To our knowledge, this is the first study to investigate developmental outcomes and life circumstances of the "third generation", i.e. children with a perinatally infected mothers.
- A particular strength is the comparison to both children of the "second generation" (i.e., with recently infected mothers) and children not affected by HIV but living in similar high-risk environments characterized by poverty and deprivation.
- While we found that children of the third generation perform equally well or better than children not directly exposed to HIV, the sample of the former was small, limiting our power to draw definite conclusion.
- Our sample consisted predominantly of young mothers. While both rates of HIV infection and young pregnancy may be compounded in deprived areas, the current findings need to be replicated in a more representative sample.

For peer review only

For peer review only - http://bmjopen.bmj.com/site/about/guidelines.xhtml

INTRODUCTION

Approximately 7.5 million people in South Africa were living with HIV in 2020, including 360,000 adolescents aged 10-19 years.[1] Generally, HIV infections have been linked to higher rates of morbidity, stigma, poverty, and other adverse outcomes.[2, 3] With the improved availability of antiretroviral therapy (ART), HIV is increasingly becoming a chronic illness [4] and rates of perinatal HIV infection are declining, mostly due to screening, treatment, and universal provision of lifelong ART, for instance in accordance with the PMTCT Option B+ guideline.[5] As the HIV epidemic enters the fourth decade since identification [6], understanding intergenerational effects of HIV is paramount. Historical exposure means that an increasing number of perinatally-infected children are now reaching adolescence and young adulthood and are becoming parents themselves, and there is a need to study their children's developmental outcomes, in order to devise effective support for these families.

HIV infections often tend to cluster in disadvantaged environments, leading to disproportionally affected communities, where risks of initial HIV infection and HIV being passed on across generations are heightened. These communities are often also affected by high adolescent pregnancy rates. Early pregnancy is common in Sub-Saharan Africa, with a weighted regional prevalence of 22% for 10- to 19-year-olds.[7] Adolescent pregnancy is on the one hand associated with a range of risks for mother and child such as poor educational prospects, exposure to poverty and violence, and poor developmental outcomes.[8, 9] On the other hand, it can also be a marker for adolescent sexual risk behaviours, and there may be a syndemic occurrence between HIV and adolescent parenthood risk among young women in Sub-Saharan Africa.[9] In this paper, we investigate the intergenerational effect of HIV in the context of adolescent motherhood, by comparing the developmental outcomes of three groups of children: 1) those who have an adolescent mother perinatally infected by HIV (termed the "third generation"), 2) those with a young mother who acquired HIV through sexual exposure

For peer review only

For peer review only - http://bmjopen.bmj.com/site/about/guidelines.xhtml

(the "second generation"), and 3) young mothers not living with HIV, but who live in similar surroundings and are likely to be HIV affected ("no direct familial HIV exposure").

To date, there have been a range of studies investigating outcomes of children of mothers living with HIV, though they commonly do not differentiate between mothers who were recently or perinatally infected. Such studies often focus on comparing HIV infected, HIV exposed and uninfected, and HIV unexposed children.[10, 11, 12] Findings suggest that the former two groups may show developmental sequelae, which can be explained by exposure to the virus as well as the environmental factors associated with HIV infection in the first place (economic, risk, stigma). However, specific knowledge on the third generation (i.e., children of mothers perinatally infected with HIV) is still extremely limited. The few existing studies focus mainly on maternal characteristics (e.g., ART adherence, age at pregnancy) and child infection status.[13] To our knowledge, no studies have yet investigated the effects of intergenerational HIV infection on child outcomes independent of child HIV status, and also in comparison to a group of children without familial HIV exposure, but who live in the same high-risk environment.

Of note, previous research on adolescents who were perinatally infected (potential mothers of the third generation) versus recently infected (potential mothers of the second generation) indicates that they may be facing different types of life challenges that could affect their parenting in divergent ways. Those perinatally infected tend to have experienced more chronic adversity, such as parental and/or sibling illness and death, caretaking responsibilities, poorer parenting and compromised parent-child relationships, poor wider family and community support, and compounded internalized and associative stigma, all of which may increase the risk for lower well-being and higher substance use.[2, 4, 14-18] Those recently infected, in contrast, have been proposed to more likely be members of marginalized groups (e.g., drug users, adolescents with street involvement), to struggle with the impacts of their

For peer review only - http://bmjopen.bmj.com/site/about/guidelines.xhtml

recent diagnosis, including biographical disruptions and reduced hope for the future, and to have difficulties with accessing and engaging in care. All of these issues may be compounded by mental health problems including anxiety, depression, substance use, and internalized stigma.[15, 18-20]

The current study was set up to explore the development of young children of adolescent and young adult mothers living in the Eastern Cape of South Africa, with a specific aim to gain a better understanding of third- and second-generation HIV effects. We had two main analysis aims: 1) to compare child developmental scores at ages 0-68 months in children of perinatally (third generation), recently infected (second generation) and non-infected mothers (independent of child HIV status) and 2) to investigate how mothers of these groups may differ in terms of their socio-economic status, well-being, and social support, all of which can shape the environment a child grows up in.

METHODS

Participants

Data utilised within these analyses originate from the "Helping Empower Youth Brought up in Adversity with their Babies and Young children" (HEY BABY) study. The study was conducted in rural and peri-urban health districts of South Africa's Eastern Cape. 1046 adolescent and young adult mothers (10-25 years) with at least one living child were interviewed between March 2018 and July 2019. The required sample size was estimated based on expected effect sizes for key outcomes. Participants were recruited through six parallel sampling strategies developed in cooperation with local experts and an adolescent mother advisory group to ensure representativeness. This comprised sampling through 73 known health facilities within the districts, 43 secondary schools, 9 maternity units, and referrals by service providers, social workers, and adolescent mothers themselves. 95%-98% of eligible

For peer review only - http://bmjopen.bmj.com/site/about/guidelines.xhtml

mothers from each recruitment channel were successfully enrolled into the study. The current analyses were limited to data on first children only, and children aged 68 months or younger who were within the normed range for the Mullen Scales (*n*= 31 excluded based on this age restriction), limiting the sample to *n* = 1015.

Patient and Public Involvement

The study team were advised on recruitment methods by adolescent mothers, whose suggestions were included in the study protocol. Furthermore, the team has been working with two Teen Advisory groups in the Eastern and Western Cape of South Africa, who were involved in piloting the study. Feedback was incorporated to improve relevance and acceptability of the questionnaire and the research procedure. Finally, through the engagement of community leaders, it was ensured that recruitment strategies were effective, sensitive to participant's circumstances and minimised stigma risks.

Procedure

The dataset analysed combined four data sources. Firstly, all adolescent mother participants completed a detailed study questionnaire relating to socio-demographic characteristics, sexual and reproductive health, physical and mental health, relationships, and social support. Secondly, they completed an adolescent parent questionnaire, which collected data on maternal and child health, child development, the father of the child, and maternal factors including social support, the parenting experience, and violence exposure. Thirdly, cognitive assessments of the children were performed by a trained administrator, using the Mullen Scales of Early Learning. Finally, details from the child's medical records (*Road to Health Booklet*) were also included in the database. All participants provided written consent, and interviews were conducted in the language of their choice (i.e., English or isiXhosa). Regardless of participation, all adolescents receive a small 'snack pack' containing a snack and

For peer review only

For peer review only - http://bmjopen.bmj.com/site/about/guidelines.xhtml

juice during the interview, and a small 'thank you pack', with personal products such as toothpaste and a toothbrush. Items included in these packs were selected by the adolescent advisory group as preferable and appropriate. New mothers also received a 'baby pack', the contents of which were also chosen by an adolescent advisory group, and included nappies and baby cream.

Research ethics statement

Ethical approvals were obtained from the Universities of Oxford (R48876/RE002) and Cape Town (HREC 226/2017) as well as University College London for secondary data analysis (14795/001). Additional local approvals and permissions were obtained from the Provincial Departments (Eastern Cape, South Africa) of Health, Education, and Social Development. The current research conforms to the principles embodied in the Declaration of Helsinki.

Data sharing agreement

The dataset is available upon request.

Measures

Mode of maternal HIV acquisition

Mode of maternal HIV acquisition (perinatal versus recent) was assessed through an algorithm, given that the study was community-based and not linked to clinical testing data. Accordingly, it was derived using a logic tree based on clinical and fieldwork experiences .[18] The algorithm allowed for categorisation according to self-report, age of ART initiation, and parental death information (Tolmay, Saal et al., in preparation). For the current analyses, we

For peer review only - http://bmjopen.bmj.com/site/about/guidelines.xhtml

compared the following three groups: children of the third generation (mother perinatally infected), children of the second generation (mother recently infected), and children of mothers not living with HIV (see Figure 1). Child HIV status was not taken into account in these classifications, since absolute numbers were low (n = 12 based on maternal self-reported data), with only one of these children being in the third generation. Thus, we were underpowered to study these children separately. We decided however to retain them in the analyses since they are relevant members of the second and third generations respectively.

Child developmental outcomes

Child developmental outcomes were assessed using the Mullen Scales of Early Development (MSEL).[21] The MSEL is normed for children aged 0 to 68 months (USA) and assesses child performance across five domains: gross motor (only for age <39 months), fine motor, visual reception, receptive and expressive language (score range: 20-80). A composite score (score range: 49-155) can be derived, and the scales have been validated for use in Sub-Saharan Africa.[22, 23, 24] Child disability status was assessed using the WHO Ten Questions Screen for Disability.[25] This measure can be applied to detect common disabilities (hearing, visual, physical, speech, mental, and epilepsy) in children. A score indicating any disability ("yes" across any of the 10 items) was derived for the current analyses.

Maternal variables

Adolescent mothers were compared on various variables, including demographic factors (maternal age, maternal and paternal age at pregnancy), child feeding method used (formula, breastfeeding, combined, other)), maternal school progression (self-report: repeated at least one school grade), poverty (number of the eight socially perceived necessities for children the family had access to, [26] household access to any government cash transfers (child support, foster child, pension, disability, or care dependency grant; South Africa's Census item [27]), food security (number of days there was not enough food for the household

For peer review only - http://bmjopen.bmj.com/site/about/guidelines.xhtml

in the past seven days [28]), full ART adherence over the past seven days (Patient Medication Adherence Questionnaire [29]), any HIV clinic appointments missed in the past year, extent of HIV-related stigma (Adolescents Living with HIV Stigma Scale [30]), depressive symptoms in the past two weeks (Child Depression Inventory-Short Form [31])), anxiety symptoms in the past month (Revised Children's Manifest Anxiety Scale [32]), PTSD symptoms in the past month (Child PTSD Checklist-Short Form [33]), suicidality symptoms during the past month (Mini International Psychiatric Interview for Children and Adolescents- Suicidality and Self-Harm Subscale [34]), extent of community violence exposure in the past year (item from the Child Exposure to Community Violence Checklist [35]), intimate partner violence exposure in the past month (Juvenile Victimization Questionnaire [36]), current parenting stress (Parental Stress Scale [37]) and amount of social support received (adjusted version of the Medical Outcomes Study Social Support Survey [38], previously used in the South African context [39]).

Statistical analyses

All analyses were conducted using STATA 16 SE. Firstly, child and adolescent mother descriptive information for the three groups of interest (children of the third generation, children of the second generation, and HIV-unexposed children) was provided. Next, child developmental outcomes and maternal factors surrounding socio-economic status, HIV-related variables (only HIV-affected mothers), mental health and social environment were compared between groups, using $\chi 2$ tests and univariate analyses of variance (ANOVA) as appropriate. For continuous variables, Tukey's range tests were used to conduct post-hoc group comparisons. Finally, a hierarchical regression model was run, with the child composite score on the Mullen scales as a key outcome. In a first step, child familial HIV exposure (third generation, second generation, HIV-unexposed) was added as a predictor. In a second step, we included relevant control variables that were found to be associated with the predictor or

For peer review only - http://bmjopen.bmj.com/site/about/guidelines.xhtml

For peer review only

outcome variables in univariate analyses or were identified as relevant within the literature to see whether child familial HIV exposure would remain a relevant predictor.

RESULTS

Descriptive variables

After applying the logic tree for mode of maternal HIV acquisition, 27 mothers (2.7%) were classified as perinatally infected, 264 (26.0%) as recently/sexually infected, and 724 (71.3%) as not living with HIV. Table 1 presents adolescent mother and child sample characteristics, split according to whether the child was in the third generation, the second generation, or not exposed to HIV. There were no significant group differences in child sex, HIV status, and early child development (ECD) program attendance; however, children of the second generation were on average older than those without HIV exposure (F= 21.20, p<0.001), likely due to maternal age differences (see below). No significant age differences were found between children of the third generation and the other two groups in post-hoc tests. Formula (without breastfeeding) use was the most common feeding method during the first six months of the child's life for mothers of the third generation (63.0%). For mothers of the second generation, formula use and breastfeeding only were similarly common (35.0% and 38.4%), while the majority of mothers of HIV-unexposed children opted for a mix of both (40.9%).

Similar to their children, mothers of the second generation (M= 19.7 years) were significantly older than those of third generation (M= 18.7 years), and the latter were older than mothers of HIV-unexposed children (M= 17.7 years) (F= 148.01, p<.001). Furthermore, mothers of the second and third generation were significantly older at pregnancy with the participating child (M= 17.8 and M= 17.3 versus M= 16.3 years respectively; F= 89.23, p <.001) compared to mothers of HIV-unexposed children. Fathers of the second generation (M= 23.2 years) were on average older at childbirth than those of the third generation and HIV-

For peer review only - http://bmjopen.bmj.com/site/about/guidelines.xhtml

unexposed children (*M*= 21.3 and *M*= 20.4 years respectively, *F*= 49.08, p <.001). There was no difference between groups regarding whether the mother lived in informal housing or a rural area, though mothers unaffected by HIV were marginally less likely to be in a relationship (64.3%, versus 77.8%% for the third generation and 71.5% for the second generation; χ^2= 6.52; p= .038). Mothers of the second generation were more likely to have repeated a school grade (69.3%), versus 61.5% for mothers of the third generation and 57.9% for mothers of HIV-unexposed children (χ^2= 9.91, p= .007).

Table 1

Sociodemographic characteristics of adolescent mothers and their children according to familial patterns of HIV infection

	Overall (*N*=1015)	Third Generation (*n*= 27)	Second Generation (*n*= 264)	No HIV Exposure (*n*= 724)	p-value
**Child Variables**					
Age in month (M, SD)	18.68 (14.69)	20.89 (16.69)	23.52 (16.44)[b]	16.83 (13.49)	<.001
Sex (1= female)	489 (48.2%)	16 (59.3%)	123 (48.2%)	350 (48.3%)	.46
HIV Status	12 (5.0%)	1 (9.1%)	11 (10.0%)	-	.92
Feeding					
Breastfeeding And Formula	347 (34.2%)	4 (14.8%)	47 (17.9%)	296 (40.9%)	<.001
Baby Formula Only	318 (31.4%)	17 (63.0%)	92 (35.0%)	209 (28.9%)	
Breastfeeding Only	258 (25.4%)	5 (18.5%)	101 (38.4%)	152 (21.0%)	
Other Milk or Water	91 (9.0%)	1 (3.7%)	23 (8.8%)	67 (9.3%)	
Attends Creche/ Early Child Development Programmes	304 (29.8%)	8 (29.6%)	80 (30.4%)	216 (29.8%)	.98
**Maternal Variables**					
Age (M, SD)	18.24 (1.83)	18.65 (1.60)[a],[b]	19.70 (1.95)[b]	17.72 (1.50)	<.001
Age at Pregnancy With The Participating Child (M, SD)	16.76 (1.70)	17.26 (1.99)	17.84 (1.90)	16.34 (1.42)	<.001
Current Relationship	670 (66.5%)	21 (77.8%)	186 (71.8%)	463 (64.2%)	.038
Paternal Age at Pregnancy (M, SD)	21.13 (3.89)	21.32 (4.25)[a]	23.17 (4.08)[b]	20.40 (3.54)	<.001
Informal Housing	217 (21.9%)	6 (23.1%)	60 (23.6%)	151 (21.2%)	.76
Living in Rural Area	293 (28.9%)	8 (29.6%)	63(23.9%)	222 (30.7%)	.11

For peer review only - http://bmjopen.bmj.com/site/about/guidelines.xhtml

Any School Grade Repeated	581 (60.8%)	16 (61.5%)	169 (69.3%)	396 (57.8%)	.007

Notes. Group comparisons were conducted using $\chi2$ tests for categorical variables and univariate analyses of variance for continuous variables. Tukey 's range post hoc tests were undertaken to identify mean differences between groups for continuous variables: [a] Statistically different from the second generation (p<0.05), [b] Statistically different from HIV unexposed group (p<0.05). Underlying *N*s vary depending on missing values (N= 933 to N= 1015).

Child developmental outcomes

Table 2 presents child developmental outcomes according to familial patterns of HIV infection. For the Mullen Scales of Early Child Development, we found that the third generation had the highest gross motor scores (M= 52.4, SD= 16.1), ahead of children not exposed to HIV (M= 50.4, SD = 12.3) and the second generation (M= 47.0, SD = 13.1; F = 6.50, p= .001). However, due to low power resulting from the small sample of children in the third generation, Tukey's range post hoc tests only identified a significant difference between the latter two groups (p= .002). Children of the second generation (M= 41.4, SD= 15.2) exhibited poorer fine motor scores than both HIV non-exposed children (M= 44.4, SD= 14.1) and children of the third generation (M= 44.3, SD= 16.1; F= 4.26, p= .014), though post-hoc tests again only indicated significant differences between the former two groups (p= .010), likely due to lack of power. No significant differences were found on the visual reception, receptive, and expressive language subscales (p=.14-.48), though scores for the second generation were consistently lowest, while children of the third generation tended to do as well or even better than non-exposed children. In terms of the composite scores, children of the second generation performed marginally lower (M= 90.6, SD= 23.0) than those not exposed to HIV (M= 94.1, SD= 20.7; p= .055) in post-hoc tests. Differences of the former to third generation (M= 94.7, SD= 22.2) were not significant in post-hoc tests due to power restrictions (p= .66), despite the scores of the third generation being approximately equal to those of non-infected children (overall model: F= 2.72 p= .066). Of note, all groups performed lower than

For peer review only - http://bmjopen.bmj.com/site/about/guidelines.xhtml

expected compared to the US reference population based on which the Mullen Scales were developed (*M*= 100.0 for the composite score; $p<.001$). No significant group differences were found for presence of any disability ($\chi^2 = .02$, $p= .90$). Finally, the rate of children who had been sick at least one day during the past month was lowest for the second generation (15.6%), with increasing rates towards the third generation (17.4%) and those not exposed to HIV (24.2%) ($\chi^2 = 8.62$, $p = .013$).

Table 2

Infant (0-68 month) developmental outcomes, according to familial patterns of HIV infection

	Overall (*N*=1015)	Third Generation (*n*= 27)	Second Generation (*n*= 264)	No HIV Exposure (*n*= 724)	p-value
**Mullen Scales of Child Development**					
Gross Motor Score*	49.61 (12.66)	52.38 (16.05)	46.96 (13.11)[b]	50.36 (12.26)	.002
Visual Reception Score	42.10 (14.24)	43.93 (14.65)	40.66 (14.28)	42.56 (14.19)	.14
Fine Motor Score	43.64 (14.59)	44.30 (16.14)	41.40 (15.17)[b]	44.44 (14.24)	.014
Receptive Language Score	47.39 (13.58)	47.74 (13.76)	46.13 (14.96)	47.84 (13.02)	.21
Expressive Language Score	51.57 (13.50)	51.74 (13.44)	50.71 (14.75)	51.88 (13.03)	.48
Composite Score	93.22 (21.43)	94.59 (23.42)	90.58 (22.98)[b]	94.13 (20.71)	.066
**WHO Disability Score- Any Disability**	242 (26.7%)	7 (26.9%)	69 (27.7%)	166 (26.2%)	.90
**Any Days Sick** (past month)	218 (21.8%)	4 (17.4%)	41 (15.6%)	173 (24.2%)	.013

Notes. Group comparisons were conducted using χ^2 tests for categorical variables and univariate analyses of variance for continuous variables. *Gross motor score: $n = 831$. Mullen subdomain scores range from 20-80, composite scores from 49-155. Tukey's range post hoc tests were undertaken to identify mean differences between groups for continuous variable: [a] Statistically different from the second generation ($p<0.05$), [b] Statistically different from HIV unexposed group ($p<0.05$).

Maternal characteristics for the three subgroups

For peer review only - http://bmjopen.bmj.com/site/about/guidelines.xhtml

Mothers of the second and third generations had on average fewer of the eight necessities available (F= 10.11, p< .001); post-hoc tests identified significant differences for second generation (p< .001) and non- significant differences for third generation mothers (p = .060) compared to those not living with HIV. Mothers of the second and third generations were also more likely to receive household cash grants (χ^2= 8.10; p= .017). However, mothers of the third generation had the lowest average days on which they had no food available for the child in the past week, followed by mothers not living with HIV, and then recently infected mothers, though the overall model was only close to significant (F=2.73; p = .066). Mothers of the second and third generations did not differ regarding ART adherence, clinic appointments missed, and HIV-related stigma (p= .69-.95). Both groups were about half as likely to have been in excellent physical health in the past twelve months as mothers of non-exposed children (χ^2= 108.46; p <.001). Mental health symptom scores were overall low, and no differences were found between groups in terms of anxiety (F= .89, p= .41) and only marginal differences for PTSD, with mothers from the second generation scoring higher than bother other groups (F= 2.38 p = .093). Mothers of the second generation also had the highest depression scores (F= 6.12, p = .002), with post-hoc tests indicating significant differences to those not HIV exposed (p = .005) and marginal differences to mothers of third generation (p = .053). Second generation mothers also scored substantially higher than both other groups on suicidality levels (F= 4.11, p = .017), though due to low power, only significant differences to mothers not living with HIV were identified in post-hoc tests (p = .017). Finally, no group differences were detected for exposure to intimate partner and community violence or parenting stress (p= .39-.97). However, mothers of the third generation indicated the lowest levels of social support, followed by the second generation and then mothers of non-exposed children (F= 3.98, p= .019), though post-hoc tests indicated only close to significant differences between mothers of the third generation and those of HIV-unexposed children (p = .061).

For peer review only - http://bmjopen.bmj.com/site/about/guidelines.xhtml

Table 3

Comparison of maternal life circumstances, according to familial patterns of HIV infection

	Overall (*N*=1015)	Third Generation (*n*= 27)	Second Generation (*n*= 264)	No HIV Exposure (*n*= 724)	p-value
**Poverty and Social Protection**					
Number of Eight Necessities Available	5.23 (2.22)	4.44 (2.69)	4.78 (2.31)[b]	5.43 (2.14)	<.001
Any Household Grant	944 (92.5%)	26 (96.3%)	254 (96.2%)	659 (91.1%)	.017
Days Insufficient Food for Child (past week)	.32 (1.07)	.04(.20)	.43 (1.22)	.28 (1.03)	.07
**HIV-associated variables**					
Full ART Adherence past 7 days	213 (75.8%)	19 (79.2%)	194 (75.5%)	-	.69
Any Clinic Appointments Missed past month	10 (4.1%)	1 (4.6%)	9 (4.1%)	-	.92
Any Stigma	56 (20.3%)	5 (20.8%)	51 (20.2%)	-	.95
**Physical and Mental Health**					
Excellent Physical Health past 12 months	598 (58.6%)	9 (33.3%)	88 (33.3%)	499 (68.9%)	<.001
Depression Score past 2 weeks (0-20)	.71 (1.42)	.30 (.67)	.96 (1.72)[b]	.64 (1.30)	.002
Anxiety Score past month (0-14)	.75 (1.88)	.74 (1.58)	.62 (1.92)	.80 (1.88)	.41
PTSD Score past month (0-36)	3.01 (5.60)	1.96 (4.93)	3.55 (5.60)	2.86 (4.94)	.09
Suicidality Score past month (0-5)	.18 (.80)	.07 (.27)	.30 (1.08)[b]	.14 (.67)	.017
**Social and Environmental Factors**					
Parenting Stress Score (18-90)	24.82 (5.75)	24.67 (4.77)	24.88 (5.64)	24.81 (5.82)	.97
Social Support Score (0-14)	13.39 (2.04)	12.74 (2.49)	13.16 (2.29)	13.49 (1.91)	.019
Extent of Intimate Partner Violence Exposure (0-4)	.13 (.56)	.08 (.40)	.17 (.66)	.11 (.53)	.39

For peer review only - http://bmjopen.bmj.com/site/about/guidelines.xhtml

Extent of Community Violence Exposure (0-4)	.27 (.79)	.22 (.64)	.30 (.84)	.26 (.78)	.79

Notes. Group comparisons were conducted using χ^2 tests for categorical variables and univariate analysis of variance for continuous variables. Tukey's range post hoc tests were undertaken to identify mean differences between groups for continuous variable: [a] Statistically different from the second generation (p<0.05), [b] Statistically different from HIV unexposed group (p<0.05). Underlying *N*s vary depending on missing values (*N*= 950 to *N*= 1015; *N* = 828 for Intimate Partner Violence- low response rates may be due to the sensitive topic).

Regression models for predicting child cognitive development composite score

Table 4 shows the results of the hierarchical linear regression model predicting the child composite score on the Mullen Scales. In step 1, compared to the baseline class of children not exposed to HIV, being a child of the second generation was associated with a lower developmental score (p= .021); no predictive effect was found for being a child of the third generation (p = .91; model: F(2,1012) = 2.72, p = .066). The former association was retained at a marginal level in step 2 (p = .059; overall model: F(8,889) = 4.08, p <.001), with mothers having repeated a school grade additionally being predictive of poorer outcomes, and higher maternal age at birth exerting a protective influence. Overall, however, the amount of variance explained by both models was relatively low.

Table 4

Multiple regression analysis predicting child developmental score from familial patterns of HIV infection and relevant child and maternal variables

	B (95% CI)	β	p-value
**Step 1**			
Mother not living with HIV (Ref.)	-		
Mother perinatally infected	.46 (-7.77; 8.69)	.00	.91
Mother recently infected	-3.5 (-6.57; -.53)	-.07	.021
Adjusted R2	.003		
**Step 2**			
Mother not living with HIV (Ref.)	-		
Mother perinatally infected	1.06 (-7.51; 9.65)	.01	.81
Mother recently infected	-3.36 (-6.85; .12)	-.07	.059
Maternal Depression Score	-.45 (-1.46; .57)	-.03	.39

For peer review only - http://bmjopen.bmj.com/site/about/guidelines.xhtml

Maternal Social Support	.21 (-.47; .89)	.02	.54
Number of Necessities	.36 (-.30; .1.03)	.04	.29
Maternal Age at Pregnancy	1.10 (.21; 1.99)	.09	.016
Mother repeated any school grade	-5.98 (-8.87; -3.07)	-.14	<.001
Number of days insufficient food for child	-.32 (-1.66; 1.01)	-.02	.64
Adjusted R2	.027		

Note. Step 2 based on *N* = 898, due to missing data. B = unstandardized regression weights. β = standardized regression weights.

DISCUSSION

In this sample of young mothers and their infants living in South Africa, we found that children of the second generation (i.e., with mothers recently infected with HIV) scored significantly lower on gross motor and fine motor functioning. For the overall composite score they tended to show lower scores as well than children not exposed to HIV. Children of the third generation (i.e., of perinatally infected mothers) appeared score similarly or even higher than children not exposed to HIV across all domains. However, the comparatively small sample size in this group makes it challenging to draw definite conclusions. Mothers also differed in their life circumstances, with mothers of the second generation showing higher rates of depression and suicidality symptoms, as well as lower food security, while mothers of the third generation indicated lower social support. No differences were found on adherence and clinical attendance variables.

Any interpretation of the current data warrants caution, due to low power resulting from the small sample size of the third-generation group. Still, our data appear to provide a tentative first indication that children of the third generation could be doing similarly well as those not exposed to familial HIV across most developmental domains. This would be promising and may indicate that families affected by HIV are in the long-term profiting from efforts to provide social protection, links to services and antiretroviral treatment, which could prevent chronic

For peer review only - http://bmjopen.bmj.com/site/about/guidelines.xhtml

adversity from being perpetuated. However, it remains to be seen whether such benefits are sustained, with studies in children living with HIV suggesting positive early developmental outcomes, but declines with age [24; Roberts et al., in preparation]. While the aim is to ultimately prevent perinatal HIV infections, larger studies of already infected young adults and their children may provide further insights into the development and needs of children of the third generation.

Children of the second generation, on the other hand, tended to do poorer across several domains, an effect that was marginally retained when controlling for maternal mental health, social support, and socio-demographic factors. The shock of maternal recent HIV diagnosis may have placed a major burden on these families, compounded by risk drivers which may have accounted for the HIV exposure in the first place, such as economic and food insecurity, or mental health struggles. This indicates that affected families may profit from additional support, including early cognitive interventions for infants [40, 41] and emotional and social support for mothers at the time of and following diagnosis. Furthermore, these mothers may need to be fast-tracked into services, given the particular double vulnerability of HIV and teen pregnancy.

Of note, the overall child sample underperformed somewhat compared to the American normative sample [21] and a similar group of children in Zimbabwe, who had caregivers living with HIV.[42] Although the level of disability did not differ across the three groups, 26% of the total sample scored for any disability. Targeted interventions could improve developmental outcomes,[43] and adapted disability services may be needed for many of these young children. The results from the current study indicate that addressing factors such as young pregnancy or poor maternal education could be of particular benefit. Maternal school progression delay in particular has been shown to be a risk factor for both early motherhood and HIV infection, as well as an outcome of perinatal HIV infection.[44, 45, 46] Therefore, it will be important to

For peer review only - http://bmjopen.bmj.com/site/about/guidelines.xhtml

For peer review only

study this factor as a risk marker for child poor developmental outcomes. However, it is important to note that our model only explained a low amount of variance overall, suggesting that other factors may be key for improving child developmental outcomes.

As described above, previous studies have found that perinatally and recently infected adolescents tend to face different challenges,[18] with the former mainly suffering from chronic effects of HIV on their family lives, while the latter tend to struggle with the impact of recent diagnoses. This seems to also extend to their experience as young parents, with perinatally infected mothers of the third generation mostly experiencing low social support, while recently infected mothers of the second generation struggled with higher food insecurity and mental-health related difficulties. This highlights that especially mothers of the second generation may require additional support in the form of social protection and access to services. Mothers of the third generation, on the other hand, appeared to have adapted to some degree, but may profit from initiatives to reduce chronic stigma and improve social support in an environment that has been long-term affected by HIV. It is important to note though that all adolescent mothers and their children in the current sample had a number of challenges to contend with. Only 24.6% attended any early child development programme of any sort (no difference by group). The mean age for first childbirth was 16.76 years, with the teens living with HIV being on average about a year older. Over 90% were in receipt of some form of household grant. This highlights the compounded disadvantages that adolescent/young mothers living in marginalized areas may face, and the urgent need for support and intervention.

The current study has several limitations. Firstly, the data collected are cross-sectional in nature, and thus do not allow any deductions about temporal relations or causality. Furthermore, they stem solely from South Africa, potentially limiting their generalizability to other countries and contexts. Thirdly, the majority of maternal data were collected through self-report, allowing the possibility for bias. Particularly maternal mental health difficulty rates

For peer review only - http://bmjopen.bmj.com/site/about/guidelines.xhtml

were overall very low in this sample, leading to the question of whether this is due to high resilience or measurement difficulties. Fourth, as the study was community-based, and clinic records were wanting, an algorithm was required to categorise perinatal infection. Fifth, the standardised developmental inventory has been used widely in Africa but is set against USA norms, and local inventories with local references groups may be more sensitive for future studies. However, this scale has been used in studies in Zimbabwe, Zambia and South Africa to good effect. Sixth, our study focused on adolescent and young mothers, and as such may not generalise to older mothers. Data on fathers may also enhance our understanding. Yet it seems important that real life multiple challenges (i.e. compounded effects of HIV exposure and young motherhood) should be appreciated, and these insights applied to wider groups. Finally, the group of children of the third generation was relatively small, which -while encouraging giving HIV preventative efforts- limited our power to investigate group differences and to perform more complex analyses, such as mediation or path models, to better understand pathways for intervention. The effects of child HIV on cognitive outcomes in the overall sample are explored elsewhere (Roberts et al., in preparation).

CONCLUSION

The current paper takes an important first look at the children of the "third generation", giving an impression of their developmental status and life circumstances. The data suggests tentatively that it is possible to break the intergenerational cycle of burden, and that third generation children can rally. It may well be that young mothers who have lived in families with HIV for many years learn to understand and accommodate the situation, have worked out pathways to support, and may not have to deal with the shock of HIV diagnosis at the time of pregnancy. Newly infected adolescents may not have the same level of family support and understanding, and they may be coming to terms with their diagnosis at the same time as adjusting to motherhood. For all children, access to early childhood programmes was low,

For peer review only - http://bmjopen.bmj.com/site/about/guidelines.xhtml

despite them having a good evidence base for enhancing child outcomes. Policies around such provisions should be incorporated for these groups. Future studies will benefit from longitudinal data, including a larger group of children of mothers perinatally infected with HIV, to better understand child cognitive development of the second and third generation, and how this is influenced by maternal factors. While the long-term aim will be to eradicate perinatal and later life HIV infections completely, it is important to further understand how to support affected families adequately.

Acknowledgements:

The authors are grateful to the young mothers and their families, the tireless data collection team, and partner organizations who supported the research process. Thanks also to Marguerite Marlow for providing training to the team on the administration of the Mullen Scales. We also thank our funders, including FHI360, the European Research Council, United States Agency for International Development, USAID, United States President's Emergency Plan for AIDS Relief, Research England, UK Medical Council, UK Department for International Development, National Institute of Health Research, Leverhulme Trust, University College London HelpAge, UNICED Eastern and Southern Africa Office, International AIDS Society, International AIDS society, UKRI GCRF Accelerating Achievement for Africa's Adolescents Hub, Fogarty International Center, National Institute on Mental Health, National Institutes of Health, Oak Foundation and the Economic and Social Research Council.

Conflicts of Interest: None to declare

Contributorship Statement: LS, ET, & LC conceptualised the overall study. ET, LC, CW, BS, NL, JJ, JC and WS were involved in study design, data collection and/or data management. KH led and KR, JT, WS, and SZ contributed to the current analyses. LS and KH were predominantly responsible for the writing of the paper, with contributions from the rest of the team. Two adolescent advisory groups served as advisors for contents and acceptability of the questionnaires and recruitment strategies. Community leaders served as advisors for recruitment.

Funding:
This study received funding from the European Research Council (ERC) under the European Union's Horizon 2020 research and innovation programme (grant agreement No 771468); the United States Agency for International Development (USAID) Cooperative Agreement No.

For peer review only - http://bmjopen.bmj.com/site/about/guidelines.xhtml

AID-OAA-LA-13-00001 and was made possible by the generous support of the American people through USAID and the United States President's Emergency Plan for AIDS Relief. The contents are the responsibility of Oxford University and do not necessarily reflect the views of USAID or the United States Government; Research England [0005218]; jointly from the UK Medical Research Council (MRC) and the UK Department for International Development (DFID) under the MRC/DFID Concordat agreement, and by the Department of Health Social Care (DHSC) through its National Institutes of Health Research (NIHR) [MR/R022372/1]; the Leverhulme Trust [PLP-2014-095]; UCL's HelpAge funding; UNICEF Eastern and Southern Africa Office (UNICEF-ESARO); from a CIPHER grant from International AIDS Society [2018/625-TOS]. The views expressed in written materials or publications [OR presented in this presentation] do not necessarily reflect the official policies of the International AIDS society; UKRI GCRF Accelerating Achievement for Africa's Adolescents (Accelerate) Hub (Grant Ref: ES/S008101/1); the Fogarty International Center, National Institute on Mental Health, National Institutes of Health under Award Number K43TW011434. The content is solely the responsibility of the authors and does not represent the official views of the National Institutes of Health; Oak Foundation [grant number: OFIL-20-057]; the Economic and Social Research Council [grant number ES/R501037/1]; the Economic and Social Research Council (grant number ES/J500112/1) the Grand Union - Doctoral Training Partnership; an Economic Social Research Council (ESRC) PhD studentship through the UBEL DTP.

REFERENCES

[1] UNAIDS (2020). Infographic- people living with HIV. https://www.unaids.org/sites/default/files/media_asset/2020_aids-data-book_en.pdf [Date Accessed: 14 May 2021]

[2] LeCroix RH, Chan WY, Henrich C et al. Maternal HIV and adolescent Functioning in South Africa: The role of the mother-child relationship. *The Journal of Early Adolescence*. 2020;40(1):83103.doi:10.1177/0272431618824726 [published Online First: 24 January 2019].

[3] Richter LM, Sherr L, Adato M, et al. Strengthening families to support children affected by HIV and AIDS. *AIDS care*. 2009;21(sup1):3-12.doi: 10.1080/09540120902923121 [published Online First: 30 September 2009]

[4] Lowenthal ED, Bakeera-Kitaka S, Marukutira et al. Perinatally acquired HIV infection in adolescents from sub-Saharan Africa: a review of emerging challenges. *The Lancet infectious diseases*. 2014;14(7):627-39.doi:10.1016/S1473-3099(13)70363-3 [published Online First: 7 January 2014].

[5] South African National Department of Health. National Consolidated Guidelines for the Prevention of Mother-To-Child Transmission of HIV (PMTCT) and the Management of HIV in Children, Adolescents and Adults. https://sahivsoc.org/Files/ART%20Guidelines%2015052015.pdf [Date Accessed: 10 September 2021].

[6] Mann JM. A worldwide pandemic. In Gottlieb MS et al., eds. Current Topics in AIDS - Volume 2. Wiley and Sons.

[7] Yah CS, Ndlovu S, Kutywayo A, et al. The prevalence of pregnancy among adolescent girls and young women across the Southern African development community economic hub: A systematic review and meta-analysis. *Health promotion perspectives*. 2020;10(4):325. doi:10.34172/hpp.2020.51 [published Online First: 7 November 2020].

[8] Glassman AL, Silverman R, McQueston K. Adolescent fertility in low-and middle-income countries: effects and solutions. Center for Global Development Working

For peer review only - http://bmjopen.bmj.com/site/about/guidelines.xhtml

Paper. 2012;295.Available at SSRN: https://ssrn.com/abstract=2102763 or http://dx.doi.org/10.2139/ssrn.2102763 [Date Accessed: 10 September 2021]
[9] Toska E, Laurenzi CA, Roberts KJ et al. Adolescent mothers affected by HIV and their children: a scoping review of evidence and experiences from sub-Saharan Africa. *Global Public Health.* 2020;15(11):1655-73.doi: 10.1080/17441692.2020.1775867 [published Online First: 6 June 2020].
[10] Banks LM, Zuurmond M, Ferrand R, Kuper H. The relationship between HIV and prevalence of disabilities in sub-Saharan Africa: systematic review (FA). *Tropical medicine & international health*. 2015; 20(4):411-29.doi: 10.1111/tmi.12449 [published Online First: 12 December 2014].
[11] McHenry MS, McAteer CI, Oyungu E et al. Neurodevelopment in young children born to HIV-infected mothers: a meta-analysis. *Pediatrics*. 2018;141(2). doi:10.1542/peds.2017-2888 [published Online First: 1 February 2018].
[12] Sherr L, Croome N, Castaneda KP et al. Developmental challenges in HIV infected children—An updated systematic review. *Children and Youth Services Review*. 2014;45:74-89.doi: 10.1016/j.childyouth.2014.03.040 [published Online First: 4 April 2014].
[13] Cruz ML, Cardoso CA, Joao EC et al. Pregnancy in HIV vertically infected adolescents and young women: a new generation of HIV-exposed infants. Aids. 2010;24(17):2727-31. doi: 10.1097/QAD.0b013e32833e50d4 [published Online First: 13 November 2010].
[14] Allen AB, Finestone M, Eloff I et al. The role of parenting in affecting the behavior and adaptive functioning of young children of HIV-infected mothers in South Africa. *AIDS and Behavior.* 2014;18(3):605-16.doi:10.1007/s10461-013-0544-7 [published Online First: 28 July 2013].
[15] Bailey H, Cruz ML, Songtaweesin WN, Puthanakit T. Adolescents with HIV and transition to adult care in the Caribbean, Central America and South America, Eastern Europe and Asia and Pacific regions. *Journal of the International AIDS Society.* 2017;20:21475.doi:10.7448/IAS.20.4.21475 [published Online First: 16 May 2017].
[16] Earnshaw VA, Kidman RC, Violari A. Stigma, depression, and substance use problems among perinatally HIV-infected youth in South Africa. *AIDS and Behavior*. 2018; 22(12):3892-6.doi: 0.1007/s10461-018-2201-7 [published Online First: 16 June 2018].
[17] Rochat T, Netsi E, Redinger S, Stein A. Parenting and HIV. *Current opinion in psychology*. 2017;15:155-61.doi: 10.1016/j.copsyc.2017.02.019 [published Online First: 22 February 2017].
[18] Sherr L, Cluver LD, Toska E, He E. Differing psychological vulnerabilities among behaviourally and perinatally HIV infected adolescents in South Africa–implications for targeted health service provision. *AIDS care*. 2018;30(sup2):92-101. 10.1080/09540121.2018.1476664 [published Online First: 30 May 2018].
[19] Anderson M, Elam G, Gerver S, et al. “It took a piece of me”: Initial responses to a positive HIV diagnosis by Caribbean people in the UK. *AIDS care*. 2010;22(12):1493-8. doi: 10.1080/09540121.2010.482125 [published Online First: 6 December 2018].
[20] Ramirez-Avila L, Regan S, Giddy J, et al. Depressive symptoms and their impact on health-seeking behaviors in newly-diagnosed HIV-infected patients in Durban, South Africa. *AIDS and Behavior*. 2012;16(8):2226-35.doi: 10.1007/s10461-012-0160-y [published Online First: 27 March 2012].
[21] Mullen EM. Mullen scales of early learning. Circle Pines, MN: AGS 1995:58-64.

For peer review only - http://bmjopen.bmj.com/site/about/guidelines.xhtml

[22] Bornman J, Romski M, Tonsing K, et al. Adapting and translating the Mullen Scales of Early Learning for the South African context. *South African Journal of Communication Disorders*. 2018;65(1):1-9.doi: 10.4102/sajcd.v65i1.571[published Online First: 8 March 2018].
[23] Koura GK, Boivin MJ, Davidson LL et al. Adaptation of the mullen scales of early learning to Benin setting. *American Journal of Epidemiology*. 2012;175(11):S128-.
[24] Milosavljevic B, Vellekoop P, Maris H, et al. Adaptation of the Mullen Scales of Early Learning for use among infants aged 5-to 24-months in rural Gambia. Developmental science. 2019;22(5):e12808. doi:10.1111/desc.12808 [published Online First: 9 February 2019].
[25] Durkin MS, Wang W, Shrout PE et al. Evaluating a Ten Questions screen for childhood disability: reliability and internal structure in different cultures. *Journal of Clinical Epidemiology*, 1995;48(5):657-66. doi:10.1016/0895-4356(94)00163-k
[26] Pillay U, Roberts B, Rule S. South African social attitudes: changing times, diverse voices. Cape Town: HSRC press 2006.
[27] Statistics South Africa. *Census 2001: Household Questionnaire*. Pretoria, SA: Statistics SA 2001.
[28] Labadarios D, Steyn NP, Maunder E et al. The national food consumption survey (NFCS): South Africa, 1999. *Public health nutrition*. 2005;8(5):533-43.doi:10.1079/phn2005816 [published Online First: 2 January 2007].
[29] Duong M, Piroth L, Grappin M et al. Evaluation of the Patient Medication Adherence Questionnaire as a tool for self-reported adherence assessment in HIV-infected patients on antiretroviral regimens. *HIV clinical trials*. 2001;2(2):128-35.doi: 10.1310/M3JR-G390-LXCM-F62G [published Online First: 29 January 2015].
[30] Pantelic M, Boyes M, Cluver L, Thabeng M. 'They say HIV is a punishment from god or from ancestors': cross-cultural adaptation and psychometric assessment of an HIV stigma scale for South African adolescents living with HIV (ALHIV-SS). *Child indicators research*. 2018;11(1):207-23. doi:10.1007/s12187-016-9428-5 [published Online First: 23 November 2016].
[31] Kovacs M. Children's depression inventory (CDI). Toronto: Multi-Health System 2003.
[32] Reynolds CR, & Richmond BO. Revised Children's Manifest Anxiety Scales (RCMAS). Los Angeles: Western Psychological Services 1985.
[33] Amaya-Jackson L, McCarthy G, Cherney MS & Newman E. Child PTSD checklist. Durham, NC: Duke University Medical Center 1995.
[34] Sheehan DV, Lecrubier Y, Sheehan KH et al. The Mini-International Neuropsychiatric Interview (MINI): the development and validation of a structured diagnostic psychiatric interview for DSM-IV and ICD-10. *Journal of clinical psychiatry*. 1998;59(20):22-33.
[35] Martin L, Revington N, Seedat S. The 39-item Child Exposure to Community Violence (CECV) Scale: Exploratory factor analysis and relationship to PTSD symptomatology in trauma-exposed children and adolescents. *International journal of behavioral medicine.* 2013;20(4):599-608.doi: 10.1007/s12529-012-9269-7 [published Online First: 2 October 2012].
[36] Finkelhor D, Hamby SL, Ormrod R, Turner H. The Juvenile Victimization Questionnaire: reliability, validity, and national norms. *Child abuse & neglect*. 2005;29(4):383-412.doi: 10.1016/j.chiabu.2004.11.001 [published Online First: 28 April 2005].
[37] Berry JO, Jones WH. The parental stress scale: Initial psychometric evidence. *Journal of social and personal relationships*. 1995 Aug;12(3):463-72.doi: 10.1177/0265407595123009

For peer review only - http://bmjopen.bmj.com/site/about/guidelines.xhtml

[38] Sherbourne CD, Stewart AL. The MOS social support survey. *Social science & medicine*. 1991;32(6):705-14.doi: 10.1016/0277-9536(91)90150-B
[39] Casale M. The importance of family and community support for the health of HIV-affected populations in Southern Africa: What do we know and where to from here?. *British Journal of Health Psychology*. 2015;20(1):21-35.doi: 10.1111/bjhp.12127 [first Published Online: 9 December 2014].
[40] Sherr L, Croome N, Bradshaw K, Parra Castaneda K. A systematic review examining whether interventions are effective in reducing cognitive delay in children infected and affected with HIV. *Aids Care*. 2014;26(sup1):S70-7.doi: 10.1080/09540121.2014.906560 [first Published Online: 10 April 2014].
[41] Thomas T, Ahmed Y, Tan M, Grigorenko EL. Cognitive and Educational Interventions for Orphans and Vulnerable Children Affected by HIV/AIDS: A Meta-Analysis. *Child development*. 2021;92(1):7-20. DOI: 10.1111/cdev.13438
[42] Mebrahtu H, Simms V, Mupambireyi Z et al. Effects of parenting classes and economic strengthening for caregivers on the cognition of HIV-exposed infants: a pragmatic cluster randomised controlled trial in rural Zimbabwe. *BMJ global health*. 2019;4(5):e001651.doi: 10.1136/bmjgh-2019-001651[published Online First: 11 September 2019].
[43] Rao N, Sun J, Wong JM et al. Early childhood development and cognitive development in developing countries: A rigorous literature review. http://cerc.edu.hku.hk/wp-content/uploads/ECD-review.pdf [Date accessed: 10 May 2021]
[44] Johnson LF, Dorrington RE, Bradshaw D et al. The effect of educational attainment and other factors on HIV risk in South African women: results from antenatal surveillance, 2000–2005. *Aids*. 2009;23(12):1583-8. doi: 10.1097/QAD.0b013e32832d407e [published Online First: 31 July 2009].
[45] Merville O, Puangmala P, Suksawas P et al. School trajectory disruption among adolescents living with perinatal HIV receiving antiretroviral treatments: a case-control study in Thailand. *BMC public health*. 2021;21(1):1-2.doi: 10.1186/s12889-021-10189-x [published Online First: 21 January 2021].
[46] Toska E, Cluver L, Orkin M, Bains A, Sherr L, Berezin M, Gulaid L. Screening and supporting through schools: Educational experiences and needs of adolescents living with HIV in a South African cohort. *BMC Public Health*. 2019;19(1):1-0. doi: 10.1186/s12889-019-6580-0 [published Online First: 6 March 2019].

For peer review only

Figure Legend

Figure 1

Flow Chart of Classification by Mode of Maternal HIV Acquisition

For peer review only - http://bmjopen.bmj.com/site/about/guidelines.xhtml

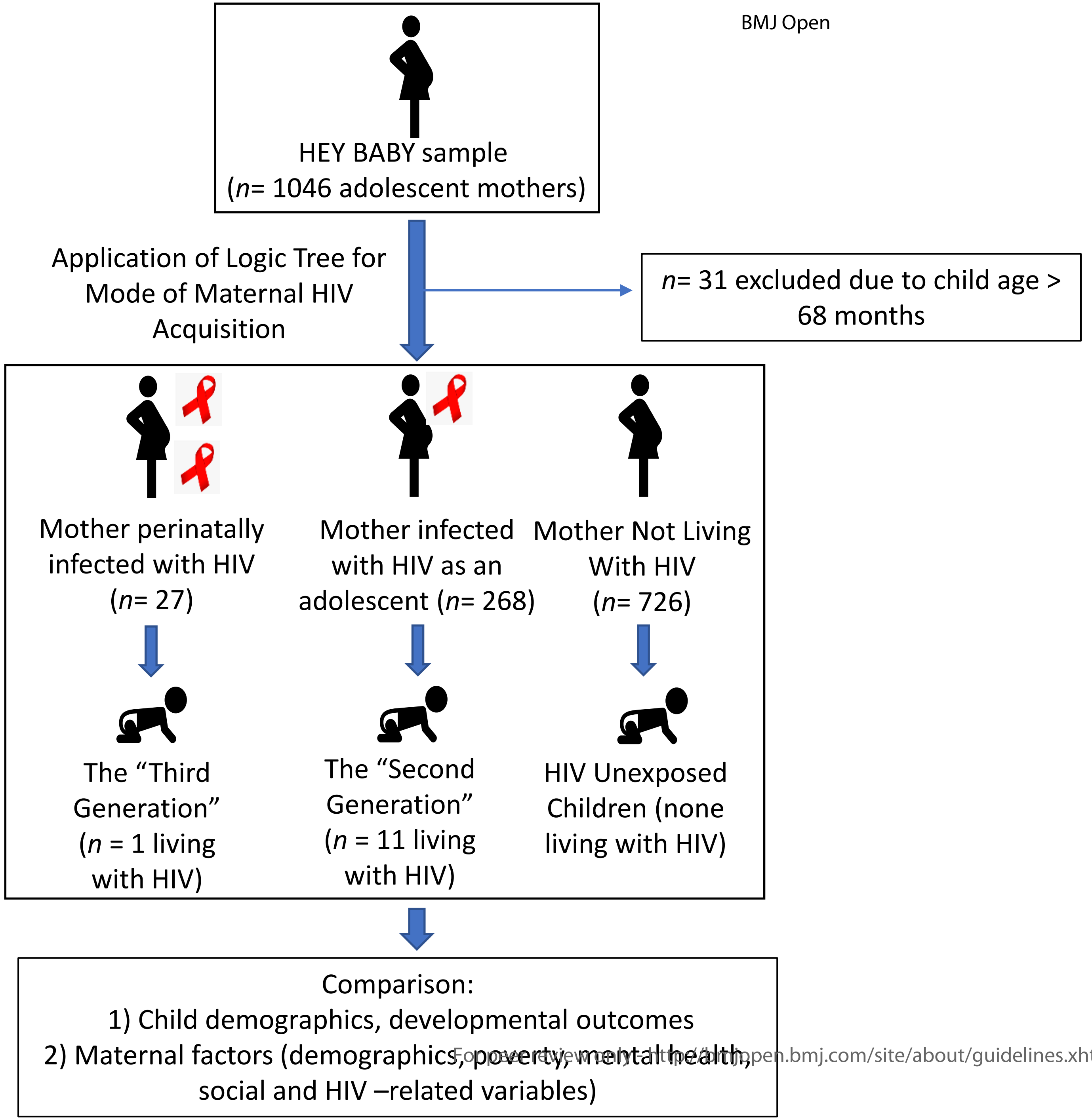

For peer review only - http://bmjopen.bmj.com/site/about/guidelines.xhtml

BMJ Open

THE CHILDREN OF YOUNG MOTHERS BORN WITH HIV – A study of the cognitive development of infants who are the Third Generation affected by HIV

Journal:	*BMJ Open*
Manuscript ID	bmjopen-2021-058340.R1
Article Type:	Original research
Date Submitted by the Author:	26-Apr-2022
Complete List of Authors:	Sherr, Lorraine; University College London<br>Haag, Katharina; UCL, Institute for Global Health<br>Roberts, Kathryn; UCL, Institute for Global Health<br>Cluver, Lucie; University of Oxford, Centre for Evidence-Based Social Intervention; University of Cape Town, Department of Psychiatry and Mental Health<br>Wittesaele, Camille; University of Oxford, Department of Social Policy & Intervention; London School of Hygiene & Tropical Medicine, Department of Infectious Disease Epidemiology<br>Saliwe, Bongiwe; University of Cape Town, Centre for Social Science Research<br>Tolmay, Janke; University of Cape Town, Centre for Social Science Research<br>Langwenya, Nontokozo; University of Cape Town, Centre for Social Science<br>Jochim, Janina; University of Oxford, Department of Social Policy & Intervention<br>Saal, Wylene; University of Cape Town, Centre for Social Science Research<br>Zhou, Siyanai; University of Cape Town, Centre for Social Science Research<br>Marlow, Marguerite; University of Stellenbosch, Institute for Life Course Health Research, Department of Global Health, Faculty of Medicine and Health Sciences<br>Chen-Charles, Jenny; University of Oxford, Department of Social Policy & Intervention<br>Toska, Elona; University of Cape Town Faculty of Humanities, Centre for Social Science Research & Department of Sociology; University of Oxford, Social Policy and Intervention
**Primary Subject Heading**:	HIV/AIDS
Secondary Subject Heading:	Global health, Mental health
Keywords:	HIV & AIDS < INFECTIOUS DISEASES, Community child health < PAEDIATRICS, EPIDEMIOLOGY, Health policy < HEALTH SERVICES ADMINISTRATION & MANAGEMENT, Developmental neurology & neurodisability < PAEDIATRICS

SCHOLARONE™
Manuscripts

I, the Submitting Author has the right to grant and does grant on behalf of all authors of the Work (as defined in the below author licence), an exclusive licence and/or a non-exclusive licence for contributions from authors who are: i) UK Crown employees; ii) where BMJ has agreed a CC-BY licence shall apply, and/or iii) in accordance with the terms applicable for US Federal Government officers or employees acting as part of their official duties; on a worldwide, perpetual, irrevocable, royalty-free basis to BMJ Publishing Group Ltd ("BMJ") its licensees and where the relevant Journal is co-owned by BMJ to the co-owners of the Journal, to publish the Work in this journal and any other BMJ products and to exploit all rights, as set out in our licence.

The Submitting Author accepts and understands that any supply made under these terms is made by BMJ to the Submitting Author unless you are acting as an employee on behalf of your employer or a postgraduate student of an affiliated institution which is paying any applicable article publishing charge ("APC") for Open Access articles. Where the Submitting Author wishes to make the Work available on an Open Access basis (and intends to pay the relevant APC), the terms of reuse of such Open Access shall be governed by a Creative Commons licence – details of these licences and which Creative Commons licence will apply to this Work are set out in our licence referred to above.

Other than as permitted in any relevant BMJ Author's Self Archiving Policies, I confirm this Work has not been accepted for publication elsewhere, is not being considered for publication elsewhere and does not duplicate material already published. I confirm all authors consent to publication of this Work and authorise the granting of this licence.

For peer review only

THE CHILDREN OF YOUNG MOTHERS BORN WITH HIV – A study of the cognitive development of infants who are the Third Generation affected by HIV

Authors: Lorraine Sherr[1], Katharina Haag[1], Kathryn Roberts[1], Lucie Cluver[2,3], Camille Wittesaele2,[4], Bongiwe Saliwe[5], Janke Tolmay[5], Nontokozo Langwenya[5], Janina Jochim[2], Wylene Saal[5], Siyanai Zhou[5], Marguerite Marlow[6], Jenny Chen-Charles[2], Elona Toska[2,5,7]

1 Institute for Global Health, University College London, UK

2 Department of Social Policy & Intervention, University of Oxford, Oxford, UK

3 Department of Psychiatry and Mental Health, University of Cape Town, Cape Town, South Africa

4 Department of Infectious Disease Epidemiology, London School of Hygiene & Tropical Medicine, London, UK

5 Centre for Social Science Research, University of Cape Town, Cape Town, South Africa

6 Institute for Life Course Health Research, Department of Global Health, Faculty of Medicine and Health Sciences, Stellenbosch University, South Africa

7 Department of Sociology, University of Cape Town, Cape Town, South Africa

Corresponding author:

Professor Lorraine Sherr (l.sherr@ucl.ac.uk), University College London, Department of Global Health, Royal Free Hospital Campus, Rowland Hill St, London, NW3 2PF

Tel: +442080168071, ext 68071

Fax: +44 207 794 1224

Word Count: 4699 (excluding title page, abstract, references, figures and tables)

For peer review only - http://bmjopen.bmj.com/site/about/guidelines.xhtml

ABSTRACT

Background: Long-term, inter-generational effects of HIV require investigation. We compared developmental outcomes of the "third generation" (i.e., children of perinatally infected mothers), the "second generation" (i.e., with recently infected mothers) and children of mothers not living with HIV.

Methods: A cross-sectional community sample of *N*=1015 young mothers (12-25 years) and their first children (2- 68 months, 48.2% female), from South Africa's Eastern Cape Province. 71.3% (*n*=724) of children were born to mothers not living with HIV; 2.7% (*n*=27; 1 living with HIV) were third and 26.0% (*n*=264; 11 living with HIV) second generation children. Child scores on the Mullen Scales of Early Learning (MSEL), the WHO Ten Questions Screen for Disability and maternal demographics were compared between groups using χ2-tests and univariate ANOVAs. Hierarchical linear regressions investigated predictive effects of familial HIV infection patterns on child MSEL composite scores, controlling for demographic and family environment variables.

Results: Second generation children performed poorer on gross (*M*=47.0, *SD*=13.1) and fine motor functioning (*M*=41.4, *SD*=15.2) and the MSEL composite score (*M*=90.6, *SD*=23.0) than children with non-infected mothers (gross motor: *M*=50.4, *SD*=12.3; fine motor: *M*=44.4, *SD*=14.1; composite score: *M*=94.1, *SD*=20.7). The third generation performed at similar levels to non-exposed children (gross motor: *M*=52.4, *SD*=16.1; fine motor: *M*=44.3, *SD*=16.1, composite score: *M*=94.7, *SD*=22.2), though analyses were underpowered for definite conclusions. Hierarchical regressions suggest marginal predictive effects of being second generation child compared to having a mother not living with HIV (*B*=-3.36, *CI*=-6.85; .12) on MSEL total scores, and non-significant predictive effects of being a third generation child (*B*= 1.06, *CI* = -7.51: 9.65) when controlling for covariates. No group differences were found for disability rates (χ^2 = .02, *p* = .90).

For peer review only - http://bmjopen.bmj.com/site/about/guidelines.xhtml

Conclusion: Longstanding familial HIV infection may facilitate care pathways and coping, explaining similar cognitive development among not exposed and third generation children. Recently infected mothers and their children may struggle due to the disruptiveness of new HIV diagnoses and incomplete access to care/ support during pregnancy and early motherhood. Targeted intervention and fast-tracking into services may improve maternal mental health and socioeconomic support.

Keywords: HIV; adolescent mothers, infants, cognitive development, mental health, environmental factors

For peer review only - http://bmjopen.bmj.com/site/about/guidelines.xhtml

STRENGTHS AND LIMITATIONS OF THIS STUDY

- To our knowledge, this is the first study to investigate developmental outcomes and life circumstances of the "third generation", i.e. children with a perinatally infected mothers.
- A particular strength is the comparison to both children of the "second generation" (i.e., with recently infected mothers) and children not affected by HIV but living in similar high-risk environments characterized by poverty and deprivation.
- While we found that children of the third generation perform equally well or better than children not directly exposed to HIV, the sample of the former was small, limiting our power to draw definite conclusion.
- Our sample consisted predominantly of young mothers. While both rates of HIV infection and young pregnancy may be compounded in deprived areas, the current findings need to be replicated in a more representative sample.

For peer review only

INTRODUCTION

Approximately 7.5 million people in South Africa were living with HIV in 2020, including 360,000 adolescents aged 10-19 years.[1] Generally, HIV infections have been linked to higher rates of morbidity, stigma, poverty, and other adverse outcomes.[2, 3] While rates of perinatal HIV infection are declining, due to improved screening and treatment options [4-6], historical exposure means that an increasing number of perinatally-infected children are now reaching adolescence and young adulthood and are becoming parents themselves. There is a need to study their children's developmental outcomes, in order to devise effective support for these families.

To knowledge of the authors, to date, there have been limited studies focusing on this "third generation", i.e., children who have adolescent mother perinatally infected by HIV. Most designs do not differentiate between mothers who were recently or perinatally infected and instead focus on comparing HIV infected, HIV exposed and uninfected, and HIV unexposed children.[7-9] Findings suggest that the former two groups may show developmental sequelae (including cognitive and motor), which can be explained by exposure to the virus (in utero) and ARTs as well as environmental factors associated with HIV infection (e.g., prematurity, poor parental mental health and substance use, stigma, caretaker changes, food insecurity).[10, 11] However, specific knowledge on the third generation (i.e., children of mothers perinatally infected with HIV) is still extremely limited. The few existing studies focus mainly on maternal characteristics (e.g., ART adherence, age at pregnancy) and child infection status.[12] To our knowledge, no studies have yet investigated the effects of intergenerational HIV infection on child outcomes independent of child HIV status, and also in comparison to a group of children without familial HIV exposure, but who live in the same high-risk environment.

Of note, previous research on adolescents who were perinatally infected (potential mothers of the third generation) versus recently infected (potential mothers of the second

For peer review only - http://bmjopen.bmj.com/site/about/guidelines.xhtml

generation) indicates that they may be facing different types of life challenges that could affect their parenting in divergent ways. Those perinatally infected tend to have experienced more chronic adversity, such as parental and/or sibling illness and death, caretaking responsibilities, poorer parenting and compromised parent-child relationships, poor wider family and community support, and compounded internalized and associative stigma, all of which may increase the risk for lower well-being and higher substance use.[2, 4, 13-17] Those recently infected, in contrast, have been proposed to more likely be members of marginalized groups (e.g., drug users, adolescents with street involvement), to struggle with the impacts of their recent diagnosis, including biographical disruptions and reduced hope for the future, and to have difficulties with accessing and engaging in care. All of these issues may be compounded by mental health problems including anxiety, depression, substance use, and internalized stigma.[14, 17-19]

The current study was set up to explore the development of young children of adolescent and young adult mothers living in the Eastern Cape of South Africa, with a specific aim to gain a better understanding of third- and second-generation HIV effects. HIV infections often tend to cluster in disadvantaged environments, leading to disproportionally affected communities, where risks of initial HIV infection and HIV being passed on across generations are heightened. These communities are often also affected by high adolescent pregnancy rates,[20] which are associated with a range of risks for mother and child such as poor educational prospects, exposure to poverty and violence, and poor developmental outcomes.[21,22] Furthermore, they can also be a marker for adolescent sexual risk behaviours, and there may be a syndemic occurrence between HIV and adolescent parenthood risk among young women in Sub-Saharan Africa.[22] The current sample, which predominantly consists of young mothers (age 19 or under) thus provides an unique opportunity to study the compounded effects of HIV and young pregnancy.

For peer review only - http://bmjopen.bmj.com/site/about/guidelines.xhtml

We had two main analysis aims: 1) to compare child developmental scores (fine and gross motor, visual reception, expressive and receptive language skills) at ages 2-68 months, independent of child HIV status, in children of mothers who were perinatally infected (third generation), recently infected through sexual exposure (second generation) and non-infected but living in the same high-risk environment) and 2) to investigate how mothers of these groups may differ in terms of their socio-economic status, well-being, and social support, all of which can shape the environment a child grows up in.

METHODS

Participants

Data utilised within these analyses originate from the "Helping Empower Youth Brought up in Adversity with their Babies and Young children" (HEY BABY) study. The study was conducted in rural and peri-urban health districts of South Africa's Eastern Cape and aimed at investigating the effects of adolescent motherhood, as well as intergenerational effects of HIV exposure. 1046 adolescent and young adult mothers (10-25 years) with at least one living child were interviewed between March 2018 and July 2019. The required sample size was estimated based on expected effect sizes for key outcomes. Participants were partially recruited from a previous study (*n*= 159: any young mothers included), as well as through six parallel sampling strategies developed in cooperation with local experts and an adolescent mother advisory group to ensure representativeness (*n*= 887: only adolescent mothers). This comprised sampling through 73 known health facilities within the districts, 43 secondary schools, 9 maternity units, and referrals by service providers, social workers, and adolescent mothers themselves. 95%-98% of eligible mothers from each recruitment channel were successfully enrolled into the study. The current analyses were limited to data on first children of the young mother only, and children aged 68 months or younger who were within the normed range for

For peer review only - http://bmjopen.bmj.com/site/about/guidelines.xhtml

the Mullen Scales (n= 31 excluded based on this age restriction), limiting the sample to n = 1015. We also performed sensitivity analyses including adolescent mothers (age at pregnancy ≤ 19, n= 972) only for the key outcomes (see Appendix 1), as these mothers had been the main recruitment focus for the study.

Patient and Public Involvement

The study team were advised on recruitment methods by adolescent mothers, whose suggestions were included in the study protocol. Furthermore, the team has been working with two Teen Advisory groups in the Eastern and Western Cape of South Africa, who were involved in piloting the study. Feedback was incorporated to improve relevance and acceptability of the questionnaire and the research procedure. Finally, through the engagement of community leaders, it was ensured that recruitment strategies were effective, sensitive to participant's circumstances and minimised stigma risks.

Procedure

The dataset analysed combined four data sources. Firstly, all adolescent mother participants completed a detailed study questionnaire relating to socio-demographic characteristics, sexual and reproductive health, physical and mental health, relationships, and social support. Secondly, they completed an adolescent parent questionnaire, which collected data on maternal and child health, child development, the father of the child, and maternal factors including social support, the parenting experience, and violence exposure. Thirdly, cognitive assessments of the children were performed by a trained administrator, using the Mullen Scales of Early Learning. Finally, details from the child's medical records (*Road to Health Booklet*) were also included in the database. All participants provided written consent, and interviews were conducted in the language of their choice (i.e., English or isiXhosa). Regardless of participation, all adolescents receive a small 'snack pack' containing a snack and

For peer review only - http://bmjopen.bmj.com/site/about/guidelines.xhtml

juice during the interview, and a small ‘thank you pack’, with personal products such as toothpaste and a toothbrush. Items included in these packs were selected by the adolescent advisory group as preferable and appropriate. New mothers also received a ‘baby pack’, the contents of which were also chosen by an adolescent advisory group, and included nappies and baby cream.

Data sharing agreement

The dataset is available upon request.

Measures

Mode of maternal HIV acquisition

Mode of maternal HIV acquisition (perinatal versus recent) was assessed through an algorithm, given that the study was community-based and not linked to clinical testing data. Accordingly, it was derived using a logic tree based on clinical and fieldwork experiences .[17] The algorithm allowed for categorisation according to self-report, age of ART initiation, and parental death information (Tolmay, Saal et al., in preparation). For the current analyses, we compared the following three groups: children of the third generation (mother perinatally infected), children of the second generation (mother recently infected), and children of mothers not living with HIV (see Figure 1). Child HIV status was not taken into account in these classifications, since absolute numbers were low (*n* = 12 based on maternal self-reported data), with only one of these children being in the third generation. Thus, we were underpowered to study these children separately. We decided however to retain them in the analyses since they are relevant members of the second and third generations respectively.

For peer review only - http://bmjopen.bmj.com/site/about/guidelines.xhtml
For peer review only

Child developmental outcomes

Child developmental outcomes were assessed using the Mullen Scales of Early Development (MSEL).[23] The MSEL is normed for children aged 0 to 68 months (USA) and assesses child performance across five domains: gross motor (only for age <39 months), fine motor, visual reception, receptive and expressive language (score range: 20-80). A composite score (score range: 49-155) can be derived, and the scales have been validated for use in Sub-Saharan Africa.[24-26] Child disability status was assessed using the WHO Ten Questions Screen for Disability.[27] This measure can be applied to detect common disabilities (hearing, visual, physical, speech, mental, and epilepsy) in children. A score indicating any disability ("yes" across any of the 10 items) was derived for the current analyses.

Maternal variables

Adolescent mothers were compared on various variables, including demographic factors (maternal age, maternal and paternal age at pregnancy), child feeding method used (formula, breastfeeding, combined, other)), maternal school progression (self-report: repeated at least one school grade), poverty (number of the eight socially perceived necessities for children the family had access to, [28] household access to any government cash transfers (child support, foster child, pension, disability, or care dependency grant; South Africa's Census item [29]), food security (number of days there was not enough food for the household in the past seven days [30]), full ART adherence over the past seven days (Patient Medication Adherence Questionnaire [31]), any HIV clinic appointments missed in the past year, extent of HIV-related stigma (Adolescents Living with HIV Stigma Scale [32]), depressive symptoms in the past two weeks (Child Depression Inventory-Short Form [33])), anxiety symptoms in the past month (Revised Children's Manifest Anxiety Scale [34]), PTSD symptoms in the past month (Child PTSD Checklist-Short Form [35]), suicidality symptoms during the past month (Mini International Psychiatric Interview for Children and Adolescents- Suicidality and Self-

For peer review only - http://bmjopen.bmj.com/site/about/guidelines.xhtml

Harm Subscale [36]), extent of community violence exposure in the past year (item from the Child Exposure to Community Violence Checklist [37]), intimate partner violence exposure in the past month (Juvenile Victimization Questionnaire [38]), current parenting stress (Parental Stress Scale [39]) and amount of social support received (adjusted version of the Medical Outcomes Study Social Support Survey [40], previously used in the South African context [41]).

Statistical analyses

All analyses were conducted using STATA 16 SE. Firstly, child and adolescent mother descriptive information for the three groups of interest (children of the third generation, children of the second generation, and HIV-unexposed children) was provided. Next, child developmental outcomes and maternal factors surrounding socio-economic status, HIV-related variables (only HIV-affected mothers), mental health and social environment were compared between groups, using $\chi 2$ tests and univariate analyses of variance (ANOVA) as appropriate. For continuous variables, Tukey's range tests were used to conduct post-hoc group comparisons. Finally, a hierarchical regression model was run, with the child composite score on the Mullen scales as a key outcome. In a first step, child familial HIV exposure (third generation, second generation, HIV-unexposed) was added as a predictor. In a second step, we included relevant control variables that were found to be associated with the predictor or outcome variables in univariate analyses or were identified as relevant within the literature to see whether child familial HIV exposure would remain a relevant predictor.

Research ethics statement

Ethical approvals were obtained from the Universities of Oxford (R48876/RE002) and Cape Town (HREC 226/2017) as well as University College London for secondary data analysis (14795/001). Additional local approvals and permissions were obtained from the

For peer review only - http://bmjopen.bmj.com/site/about/guidelines.xhtml

Provincial Departments (Eastern Cape, South Africa) of Health, Education, and Social Development. The current research conforms to the principles embodied in the Declaration of Helsinki.

RESULTS

Descriptive variables

After applying the logic tree for mode of maternal HIV acquisition, 27 mothers (2.7%) were classified as perinatally infected, 264 (26.0%) as recently/sexually infected, and 724 (71.3%) as not living with HIV. Table 1 presents adolescent mother and child sample characteristics, split according to whether the child was in the third generation, the second generation, or not exposed to HIV. There were no significant group differences in child sex, HIV status, and creche attendance; however, children of the second generation were on average older than those without HIV exposure (F= 21.20, p<0.001), likely due to maternal age differences (see below). No significant age differences were found between children of the third generation and the other two groups in post-hoc tests. Formula (without breastfeeding) use was the most common feeding method during the first six months of the child's life for mothers of the third generation (63.0%). For mothers of the second generation, formula use and breastfeeding only were similarly common (35.0% and 38.4%), while the majority of mothers of HIV-unexposed children opted for a mix of both (40.9%).

Similar to their children, mothers of the second generation (M= 19.7 years) were significantly older than those of third generation (M= 18.7 years), and the latter were older than mothers of HIV-unexposed children (M= 17.7 years) (F= 148.01, p<.001). Furthermore, mothers of the second and third generation were significantly older at pregnancy with the participating child (M= 17.8 and M= 17.3 versus M= 16.3 years respectively; F= 89.23, p <.001) compared to mothers of HIV-unexposed children. Fathers of the second generation (M=

For peer review only - http://bmjopen.bmj.com/site/about/guidelines.xhtml

23.2 years) were on average older at childbirth than those of the third generation and HIV-unexposed children (*M*= 21.3 and *M*= 20.4 years respectively, $F= 49.08$, $p <.001$). There was no difference between groups regarding whether the mother lived in informal housing or a rural area, though mothers unaffected by HIV were marginally less likely to be in a relationship (64.3%, versus 77.8%% for the third generation and 71.5% for the second generation; $\chi^2= 6.52$; $p= .038$). Mothers of the second generation were more likely to have repeated a school grade (69.3%), versus 61.5% for mothers of the third generation and 57.9% for mothers of HIV-unexposed children ($\chi^2= 9.91$, $p= .007$).

Table 1

Sociodemographic characteristics of adolescent mothers and their children according to familial patterns of HIV infection

	Overall (*N*=1015)	Third Generation (*n*= 27)	Second Generation (*n*= 264)	No HIV Exposure (*n*= 724)	p-value
**Child Variables**					
Age in months (M, SD)	18.7 (14.7)	20.9 (16.7)	23.5 (16.4)[b]	16.8 (13.5)	<.001
Sex (1= female)	489 (48.2%)	16 (59.3%)	123 (48.2%)	350 (48.3%)	.46
HIV Status	12 (5.0%)	1 (9.1%)	11 (10.0%)	-	.92
Feeding					
Breastfeeding And Formula	347 (34.2%)	4 (14.8%)	47 (17.9%)	296 (40.9%)	<.001
Baby Formula Only	318 (31.4%)	17 (63.0%)	92 (35.0%)	209 (28.9%)	
Breastfeeding Only	258 (25.4%)	5 (18.5%)	101 (38.4%)	152 (21.0%)	
Other Milk or Water	91 (9.0%)	1 (3.7%)	23 (8.8%)	67 (9.3%)	
Creche Attendance	304 (29.8%)	8 (29.6%)	80 (30.4%)	216 (29.8%)	.98
**Maternal Variables**					
Age in years (M, SD)	18.24 (1.8)	18.7 (1.6)[a],[b]	19.7 (2.0)[b]	17.7 (1.5)	<.001
Age at Pregnancy With The Participating Child (years; M, SD)	16.8 (1.7)	17.3 (2.0)	17.8 (1.9)	16.3 (1.4)	<.001
Paternal Age at Pregnancy (years, M, SD)	21.1 (3. 9)	21.3 (4.3)[a]	23.2 (4.08)[b]	20.4 (3.5)	<.001
Informal Housing	217 (21.9%)	6 (23.1%)	60 (23.6%)	151 (21.2%)	.76

For peer review only - http://bmjopen.bmj.com/site/about/guidelines.xhtml

Living in Rural Area	293 (28.9%)	8 (29.6%)	63(23.9%)	222 (30.7%)	.11
Any School Grade Repeated	581 (60.8%)	16 (61.5%)	169 (69.3%)	396 (57.8%)	.007
Current Relationship	670 (66.5%)	21 (77.8%)	186 (71.8%)	463 (64.2%)	.038

Notes. Group comparisons were conducted using χ2 tests for categorical variables and univariate analyses of variance for continuous variables. Tukey 's range post hoc tests were undertaken to identify mean differences between groups for continuous variables: [a] Statistically different from the second generation (*p*<0.05), [b] Statistically different from HIV unexposed group (*p*<0.05). Underlying *N*s vary depending on missing values (*N*= 933 to *N*= 1015).

Child developmental outcomes

Table 2 presents child developmental outcomes according to familial patterns of HIV infection. For the Mullen Scales of Early Child Development, we found that the third generation had the highest gross motor scores (*M*= 52.4, *SD*= 16.1), ahead of children not exposed to HIV (*M*= 50.4, *SD* = 12.3) and the second generation (*M*= 47.0, *SD* = 13.1; *F* = 6.50, *p*= .001). However, due to low power resulting from the small sample of children in the third generation, Tukey's range post hoc tests only identified a significant difference between the latter two groups (*Diff*= 3.41, *95% CI*= 1.08; 5.73) Children of the second generation (*M*= 41.4, *SD*= 15.2) exhibited poorer fine motor scores than both HIV non-exposed children (*M*= 44.4, *SD*= 14.1) and children of the third generation (*M*= 44.3, *SD*= 16.1; *F*= 4.26, *p*= .014), though post-hoc tests again only indicated significant differences between the former two groups (*Diff*= 3.04, *95%CI*= .59: 5.49. No significant differences were found on the visual reception, receptive, and expressive language subscales (*p*=.14-.48), though scores for the second generation were consistently lowest, while children of the third generation tended to do as well or even better than non-exposed children. In terms of the composite scores, children of the second generation performed marginally lower (*M*= 90.6, *SD*= 23.0) than those not exposed to HIV (*M*= 94.1, *SD*= 20.7; *p*= .055) in post-hoc tests (*Diff*= 3.55, *95%CI*= -.05; 7.16). Differences of the former to third generation (*M*= 94.7, *SD*= 22.2) were not significant in post-hoc tests (*p*= .66), despite the scores of the third generation being approximately equal to those

For peer review only - http://bmjopen.bmj.com/site/about/guidelines.xhtml

of non-infected children (overall model: F= 2.72 p= .066). Of note, all groups performed lower than expected compared to the US reference population based on which the Mullen Scales were developed (M= 100.0 for the composite score; p<.001). No significant group differences were found for presence of any disability (χ^2 = .02, p= .90). Finally, the rate of children who had been sick at least one day during the past month was lowest for the second generation (15.6%), with increasing rates towards the third generation (17.4%) and those not exposed to HIV (24.2%) (χ^2 = 8.62, p = .013). When limiting the sample to adolescent mothers (age <=19) only, the same magnitude and directions of findings were obtained (see Appendix 1).

Table 2

Infant (0-68 month) developmental outcomes, according to familial patterns of HIV infection

	Overall (*N*=1015)	Third Generation (*n*= 27)	Second Generation (*n*= 264)	No HIV Exposure (*n*= 724)	p-value
**Mullen Scales of Child Development**					
Gross Motor Score*	49.6 (12.7)	52.4(16.1)	47.0 (13.1)[b]	50.4 (12.3)	.002
Visual Reception Score	42.1 (14.2)	43.9 (14.7)	40.7 (14.3)	42.6 (14.2)	.14
Fine Motor Score	43.6 (14.6)	44.3 (16.1)	41.4 (15.2)[b]	44.4 (14.2)	.014
Receptive Language Score	47.4 (13.6)	47.7 (13.8)	46.1 (15.0)	47.8 (13.0)	.21
Expressive Language Score	51.6 (13.5)	51.7 (13.4)	50.7 (14.8)	51.9 (13.0)	.48
Composite Score	93.2 (21.4)	94.6 (23.4)	90.6 (23.0)[b]	94.1 (20.7)	.066
**WHO Disability Score- Any Disability**	242 (26.7%)	7 (26.9%)	69 (27.7%)	166 (26.2%)	.90
**Any Days Sick** (past month)	218 (21.8%)	4 (17.4%)	41 (15.6%)	173 (24.2%)	.013

Notes. Group comparisons were conducted using χ^2 tests for categorical variables and univariate analyses of variance for continuous variables. *Gross motor score: n = 831. Mullen subdomain scores range from 20-80, composite scores from 49-155. Tukey's range post hoc tests were undertaken to identify mean differences between groups for continuous variable: [a] Statistically different from the second generation (p<0.05), [b] Statistically different from HIV unexposed group (p<0.05).

Maternal characteristics for the three subgroups

For peer review only - http://bmjopen.bmj.com/site/about/guidelines.xhtml

Mothers of the second and third generations had on average fewer of the eight necessities available (F= 10.11, p< .001); post-hoc tests identified significant differences for second generation (p< .001) and non- significant differences for third generation mothers (p = .060) compared to those not living with HIV. Mothers of the second and third generations were also more likely to receive household cash grants (χ^2= 8.10; p= .017). However, mothers of the third generation had the lowest average days on which they had no food available for the child in the past week, followed by mothers not living with HIV, and then recently infected mothers, though the overall model was only close to significant (F=2.73; p = .066). Mothers of the second and third generations did not differ regarding ART adherence, clinic appointments missed, and HIV-related stigma (p= .69-.95). Both groups were about half as likely to have been in excellent physical health in the past twelve months as mothers of non-exposed children (χ^2= 108.46; p <.001).

Mental health symptom scores were overall low, and no differences were found between groups in terms of anxiety (F= .89, p= .41) and only marginal differences for PTSD, with mothers from the second generation scoring higher than bother other groups (F= 2.38 p = .093). Mothers of the second generation also had the highest depression scores (F= 6.12, p = .002), with post-hoc tests indicating significant differences to those not HIV exposed (p = .005) and marginal differences to mothers of third generation (p = .053). Second generation mothers also scored substantially higher than both other groups on suicidality levels (F= 4.11, p = .017), though due to low power, only significant differences to mothers not living with HIV were identified in post-hoc tests (p = .017). Finally, no group differences were detected for exposure to intimate partner and community violence or parenting stress (p= .39-.97). However, mothers of the third generation indicated the lowest levels of social support, followed by the second generation and then mothers of non-exposed children (F= 3.98, p= .019), though post-hoc tests

For peer review only - http://bmjopen.bmj.com/site/about/guidelines.xhtml

indicated only close to significant differences between mothers of the third generation and those of HIV-unexposed children (p = .061).

Table 3

Comparison of maternal life circumstances, according to familial patterns of HIV infection

	Overall (*N*=1015)	Third Generation (*n*= 27)	Second Generation (*n*= 264)	No HIV Exposure (*n*= 724)	p-value
**Poverty and Social Protection**					
Number of Eight Necessities Available	5.2 (2.2)	4.4 (2.7)	4.8 (2.3)[b]	5.4 (2.1)	<.001
Any Household Grant	944 (92.5%)	26 (96.3%)	254 (96.2%)	659 (91.1%)	.017
Days Insufficient Food for Child (past week)	.3 (1.1)	.0 (.2)	.4 (1.2)	.3 (1.0)	.07
**HIV-associated variables**					
Full ART Adherence past 7 days	213 (75.8%)	19 (79.2%)	194 (75.5%)	-	.69
Any Clinic Appointments Missed past month	10 (4.1%)	1 (4.6%)	9 (4.1%)	-	.92
Any Stigma	56 (20.3%)	5 (20.8%)	51 (20.2%)	-	.95
**Physical and Mental Health**					
Excellent Physical Health past 12 months	598 (58.6%)	9 (33.3%)	88 (33.3%)	499 (68.9%)	<.001
Depression Score past 2 weeks (0-20)	.7 (1.4)	.3 (.7)	1.0 (1.7)[b]	.6 (1.3)	.002
Anxiety Score past month (0-14)	.8 (1.9)	.7 (1.6)	.6 (1.9)	.8 (1.9)	.41
PTSD Score past month (0-36)	3.0 (5.6)	2.0 (4.9)	3.6 (5.6)	2.9 (4.9)	.09
Suicidality Score past month (0-5)	.2 (.8)	.1 (.3)	.3 (1.1)[b]	.1 (.7)	.017
**Social and Environmental Factors**					
Parenting Stress Score (18-90)	24.8 (5.8)	24.7 (4.8)	24.9 (5.6)	24.8 (5.8)	.97

For peer review only - http://bmjopen.bmj.com/site/about/guidelines.xhtml

Social Support Score (0-14)	13.4 (2.04)	12.7 (2.5)	13.2(2.3)	13.5 (1.9)	.019
Extent of Intimate Partner Violence Exposure (0-4)	.1 (.6)	.1 (.4)	.2 (.7)	.1 (.5)	.39
Extent of Community Violence Exposure (0-4)	.3 (.8)	.2 (.6)	.3 (.8)	.3 (.8)	.79

Notes. Group comparisons were conducted using χ^2 tests for categorical variables and univariate analysis of variance for continuous variables. Tukey's range post hoc tests were undertaken to identify mean differences between groups for continuous variable: [a] Statistically different from the second generation (p<0.05), [b] Statistically different from HIV unexposed group (p<0.05). Underlying *N*s vary depending on missing values (N= 950 to N= 1015; N = 828 for Intimate Partner Violence- low response rates may be due to the sensitive topic).

Regression models for predicting child cognitive development composite score

Table 4 shows the results of the hierarchical linear regression model predicting the child composite score on the Mullen Scales. In step 1, compared to the baseline class of children not exposed to HIV, being a child of the second generation was associated with a lower developmental score (p= .021); no predictive effect was found for being a child of the third generation (p = .91; model: F(2,1012) = 2.72, p = .066). The former association was retained at a marginal level in the adjusted model in step 2 (p = .059; overall model: F(8,889) = 4.08, p <.001), with mothers having repeated a school grade additionally being predictive of poorer outcomes, and higher maternal age at birth exerting a protective influence. Overall, however, the amount of variance explained by both models was relatively low.

Table 4

Multiple regression analysis predicting child developmental score from familial patterns of HIV infection in step 1, with step 2 controlling for relevant child and maternal variables

	B (95% CI)	β	p-value
**Step 1: Unadjusted model**			
Mother not living with HIV (Ref.)	-		
Mother perinatally infected	.46 (-7.77; 8.69)	.00	.91
Mother recently infected	-3.5 (-6.57; -.53)	-.07	.021
Adjusted R2	.003		

For peer review only - http://bmjopen.bmj.com/site/about/guidelines.xhtml

**Step 2: Adjusted model**			
Mother not living with HIV (Ref.)	-		
Mother perinatally infected	1.06 (-7.51; 9.65)	.01	.81
Mother recently infected	-3.36 (-6.85; .12)	-.07	.059
Maternal Depression Score	-.45 (-1.46; .57)	-.03	.39
Maternal Social Support	.21 (-.47; .89)	.02	.54
Number of Necessities	.36 (-.30; .1.03)	.04	.29
Maternal Age at Pregnancy	1.10 (.21; 1.99)	.09	.016
Mother repeated any school grade	-5.98 (-8.87; -3.07)	-.14	<.001
Number of days insufficient food for child	-.32 (-1.66; 1.01)	-.02	.64
Adjusted R2	.027		

Note. Step 2 (adjusted model) based on $N = 898$, due to missing data on the control variables. B = unstandardized regression weights. β = standardized regression weights.

DISCUSSION

In this sample of young mothers and their infants living in South Africa, we found that children of the second generation (i.e., with mothers recently infected with HIV) scored significantly lower on gross motor and fine motor functioning. For the overall composite score they tended to show lower scores as well than children not exposed to HIV. Children of the third generation (i.e., of perinatally infected mothers) appeared score similarly or even higher than children not exposed to HIV across all domains. However, the comparatively small sample size in this group makes it challenging to draw definite conclusions. Mothers also differed in their life circumstances, with mothers of the second generation showing higher rates of depression and suicidality symptoms, as well as lower food security, while mothers of the third generation indicated lower social support. No differences were found on adherence and clinical attendance variables.

Any interpretation of the current data warrants caution, due to low power resulting from the small sample size of the third-generation group. Still, our data appear to provide a tentative first indication that children of the third generation could be doing similarly well as those not

For peer review only - http://bmjopen.bmj.com/site/about/guidelines.xhtml

exposed to familial HIV across most developmental domains. This would be promising and may indicate that families affected by HIV are in the long-term profiting from efforts to provide social protection, links to services and antiretroviral treatment, which could prevent chronic adversity from being perpetuated. However, it remains to be seen whether such benefits are sustained, with studies in children living with HIV suggesting positive early developmental outcomes, but declines with age [26; Roberts et al., in preparation]. While the aim is to ultimately prevent perinatal HIV infections, larger studies of already infected young adults and their children may provide further insights into the development and needs of children of the third generation.

Children of the second generation, on the other hand, tended to do poorer across several domains, an effect that was marginally retained when controlling for maternal mental health, social support, and socio-demographic factors. The shock of maternal recent HIV diagnosis may have placed a major burden on these families, compounded by risk drivers which may have accounted for the HIV exposure in the first place, such as economic and food insecurity, or mental health struggles. This indicates that affected families may profit from additional support, including early cognitive interventions for infants [10, 42] and emotional and social support for mothers at the time of and following diagnosis. Furthermore, these mothers may need to be fast-tracked into services, given the particular double vulnerability of HIV and teen pregnancy.

Of note, the overall child sample underperformed somewhat compared to the American normative sample [23] and a similar group of children in Zimbabwe, who had caregivers living with HIV.[43] Although the level of disability did not differ across the three groups, 26% of the total sample scored for any disability. Targeted interventions could improve developmental outcomes,[44] and adapted disability services may be needed for many of these young children. The results from the current study indicate that addressing factors such as young pregnancy or

For peer review only - http://bmjopen.bmj.com/site/about/guidelines.xhtml

poor maternal education could be of particular benefit. Maternal school progression delay in particular has been shown to be a risk factor for both early motherhood and HIV infection, as well as an outcome of perinatal HIV infection.[45, 46, 47] Therefore, it will be important to study this factor as a risk marker for child poor developmental outcomes. However, it is important to note that our model only explained a low amount of variance overall, suggesting that other factors may be key for improving child developmental outcomes.

As described above, previous studies have found that perinatally and recently infected adolescents tend to face different challenges,[16] with the former mainly suffering from chronic effects of HIV on their family lives, while the latter tend to struggle with the impact of recent diagnoses. This seems to also extend to their experience as young parents, with perinatally infected mothers of the third generation mostly experiencing low social support, while recently infected mothers of the second generation struggled with higher food insecurity and mental-health related difficulties. This highlights that especially mothers of the second generation may require additional support in the form of social protection and access to services. Mothers of the third generation, on the other hand, appeared to have adapted to some degree, but may profit from initiatives to reduce chronic stigma and improve social support in an environment that has been long-term affected by HIV. It is important to note though that all adolescent mothers and their children in the current sample had a number of challenges to contend with. Only 24.6% attended creche (no difference by group). The mean age for first childbirth was 16.76 years, with the teens living with HIV being on average about a year older. Over 90% were in receipt of some form of household grant. This highlights the compounded disadvantages that adolescent/young mothers living in marginalized areas may face, and the urgent need for support and intervention.

The current study has several limitations. Firstly, the data collected are cross-sectional in nature, and thus do not allow any deductions about temporal relations or causality.

For peer review only - http://bmjopen.bmj.com/site/about/guidelines.xhtml

Furthermore, they stem solely from South Africa, potentially limiting their generalizability to other countries and contexts. Thirdly, the majority of maternal data were collected through self-report, allowing the possibility for bias. Particularly maternal mental health difficulty rates were overall very low in this sample, leading to the question of whether this is due to high resilience or measurement difficulties. Fourth, as the study was community-based, and clinic records were wanting, an algorithm was required to categorise perinatal infection. Fifth, the standardised developmental inventory has been used widely in Africa but is set against USA norms, and local inventories with local references groups may be more sensitive for future studies. However, this scale has been used in studies in Zimbabwe, Zambia and South Africa to good effect. Sixth, while our sample predominantly comprised adolescent mothers, some older mothers up to age 25 whom we had historical data for were also included in our analyses to increase analytical power. This limited our ability to draw conclusions for adolescent mothers specifically. Furthermore, our study may not generalise to older mothers and data on fathers could help to further enhance our understanding. Yet it seems important that real life multiple challenges (i.e., compounded effects of HIV exposure and young motherhood) should be appreciated, and these insights applied to wider groups. Finally, the group of children of the third generation was relatively small, which -while encouraging giving HIV preventative efforts- limited our power to investigate group differences and to perform more complex analyses, such as mediation or path models, to better understand pathways for intervention. Future studies may aim at specifically recruiting a larger sample of third generation children to replicate and extend our findings. The effects of child HIV on cognitive outcomes in the overall sample are explored elsewhere (Roberts et al., in preparation).

CONCLUSION

The current paper takes an important first look at the children of the "third generation", giving an impression of their developmental status and life circumstances. The data suggests

For peer review only - http://bmjopen.bmj.com/site/about/guidelines.xhtml

tentatively that it is possible to break the intergenerational cycle of burden, and that third generation children can rally. It may well be that young mothers who have lived in families with HIV for many years learn to understand and accommodate the situation, have worked out pathways to support, and may not have to deal with the shock of HIV diagnosis at the time of pregnancy. Newly infected adolescents may not have the same level of family support and understanding, and they may be coming to terms with their diagnosis at the same time as adjusting to motherhood. For all children, access to early childhood programmes was low, despite them having a good evidence base for enhancing child outcomes. Policies around such provisions should be incorporated for these groups. Future studies will benefit from longitudinal data, including a larger group of children of mothers perinatally infected with HIV, to better understand child cognitive development of the second and third generation, and how this is influenced by maternal factors. While the long-term aim will be to eradicate perinatal and later life HIV infections completely, it is important to further understand how to support affected families adequately.

For peer review only

Acknowledgements:

The authors are grateful to the young mothers and their families, the tireless data collection team, and partner organizations who supported the research process. Thanks also to Marguerite Marlow for providing training to the team on the administration of the Mullen Scales. We also thank our funders, including FHI360, the European Research Council, United States Agency for International Development, USAID, United States President's Emergency Plan for AIDS Relief, Research England, UK Medical Council, UK Department for International Development, National Institute of Health Research, Leverhulme Trust, University College London HelpAge, UNICED Eastern and Southern Africa Office, International AIDS Society, International AIDS society, UKRI GCRF Accelerating Achievement for Africa's Adolescents Hub, Fogarty International Center, National Institute on Mental Health, National Institutes of Health, Oak Foundation and the Economic and Social Research Council.

Conflicts of Interest: None to declare

Contributorship Statement: LS, ET, & LC conceptualised the overall study. ET, LC, CW, BS, NL, JJ, JC and WS were involved in study design, data collection and/or data management. KH led and KR, JT, WS, and SZ contributed to the current analyses. LS and KH were predominantly responsible for the writing of the paper, with contributions from the rest of the team. Two adolescent advisory groups served as advisors for contents and acceptability of the questionnaires and recruitment strategies. Community leaders served as advisors for recruitment.

Funding:
This study received funding from the European Research Council (ERC) under the European Union's Horizon 2020 research and innovation programme (grant agreement No 771468); the United States Agency for International Development (USAID) Cooperative Agreement No. AID-OAA-LA-13-00001 and was made possible by the generous support of the American people through USAID and the United States President's Emergency Plan for AIDS Relief. The contents are the responsibility of Oxford University and do not necessarily reflect the views of USAID or the United States Government; Research England [0005218]; jointly from the UK Medical Research Council (MRC) and the UK Department for International Development (DFID) under the MRC/DFID Concordat agreement, and by the Department of Health Social Care (DHSC) through its National Institutes of Health Research (NIHR) [MR/R022372/1]; the Leverhulme Trust [PLP-2014-095]; UCL's HelpAge funding; UNICEF Eastern and Southern Africa Office (UNICEF-ESARO); from a CIPHER grant from International AIDS Society [2018/625-TOS]. The views expressed in written materials or publications [OR presented in this presentation] do not necessarily reflect the official policies of the International AIDS society; UKRI GCRF Accelerating Achievement for Africa's Adolescents (Accelerate) Hub (Grant Ref: ES/S008101/1); the Fogarty International Center, National Institute on Mental Health, National Institutes of Health under Award Number

For peer review only - http://bmjopen.bmj.com/site/about/guidelines.xhtml

K43TW011434. The content is solely the responsibility of the authors and does not represent the official views of the National Institutes of Health; Oak Foundation [grant number: OFIL-20-057]; the Economic and Social Research Council [grant number ES/R501037/1]; the Economic and Social Research Council (grant number ES/J500112/1) the Grand Union - Doctoral Training Partnership; an Economic Social Research Council (ESRC) PhD studentship through the UBEL DTP.

REFERENCES

[1] UNAIDS (2020). Infographic- people living with HIV. https://www.unaids.org/sites/default/files/media_asset/2020_aids-data-book_en.pdf [Date Accessed: 14 May 2021]

[2] LeCroix RH, Chan WY, Henrich C et al. Maternal HIV and adolescent Functioning in South Africa: The role of the mother-child relationship. *The Journal of Early Adolescence*. 2020;40(1):83103.doi:10.1177/0272431618824726 [published Online First: 24 January 2019].

[3] Richter LM, Sherr L, Adato M, et al. Strengthening families to support children affected by HIV and AIDS. *AIDS care*. 2009;21(sup1):3-12.doi: 10.1080/09540120902923121 [published Online First: 30 September 2009]

[4] Lowenthal ED, Bakeera-Kitaka S, Marukutira et al. Perinatally acquired HIV infection in adolescents from sub-Saharan Africa: a review of emerging challenges. *The Lancet infectious diseases*. 2014;14(7):627-39.doi:10.1016/S1473-3099(13)70363-3 [published Online First: 7 January 2014].

[5] South African National Department of Health. National Consolidated Guidelines for the Prevention of Mother-To-Child Transmission of HIV (PMTCT) and the Management of HIV in Children, Adolescents and Adults. https://sahivsoc.org/Files/ART%20Guidelines%2015052015.pdf [Date Accessed: 10 September 2021].

[6] Mann JM. A worldwide pandemic. In Gottlieb MS et al., eds. Current Topics in AIDS - Volume 2. Wiley and Sons.

[7] Banks LM, Zuurmond M, Ferrand R, Kuper H. The relationship between HIV and prevalence of disabilities in sub-Saharan Africa: systematic review (FA). *Tropical medicine & international health*. 2015; 20(4):411-29.doi: 10.1111/tmi.12449 [published Online First: 12 December 2014].

[8] McHenry MS, McAteer CI, Oyungu E et al. Neurodevelopment in young children born to HIV-infected mothers: a meta-analysis. *Pediatrics*. 2018;141(2). doi:10.1542/peds.2017-2888 [published Online First: 1 February 2018].

[9] Sherr L, Croome N, Castaneda KP et al. Developmental challenges in HIV infected children—An updated systematic review. *Children and Youth Services Review*. 2014;45:74-89.doi: 10.1016/j.childyouth.2014.03.040 [published Online First: 4 April 2014].

[10] Sherr L, Croome N, Bradshaw K, Parra Castaneda K. A systematic review examining whether interventions are effective in reducing cognitive delay in children infected and affected with HIV. *Aids Care*. 2014;26(sup1):S70-7.doi: 10.1080/09540121.2014.906560 [first Published Online: 10 April 2014].

[11] Townsend C, Schulte J, Thorne C et al. Antiretroviral therapy and preterm delivery-a pooled analysis of data from the United States and Europe. *BJOG : an international journal of obstetrics and gynaecology*.2010; 117(11):1399–1410. doi:10.1111/j.1471-0528.2010.02689.x

[12] Cruz ML, Cardoso CA, Joao EC et al. Pregnancy in HIV vertically infected adolescents and young women: a new generation of HIV-exposed infants. Aids.

For peer review only - http://bmjopen.bmj.com/site/about/guidelines.xhtml

2010;24(17):2727-31. doi: 10.1097/QAD.0b013e32833e50d4 [published Online First: 13 November 2010].
[13] Allen AB, Finestone M, Eloff I et al. The role of parenting in affecting the behavior and adaptive functioning of young children of HIV-infected mothers in South Africa. *AIDS and Behavior.* 2014;18(3):605-16.doi:10.1007/s10461-013-0544-7 [published Online First: 28 July 2013].
[14] Bailey H, Cruz ML, Songtaweesin WN, Puthanakit T. Adolescents with HIV and transition to adult care in the Caribbean, Central America and South America, Eastern Europe and Asia and Pacific regions. *Journal of the International AIDS Society.* 2017;20:21475.doi:10.7448/IAS.20.4.21475 [published Online First: 16 May 2017].
[15] Earnshaw VA, Kidman RC, Violari A. Stigma, depression, and substance use problems among perinatally HIV-infected youth in South Africa. *AIDS and Behavior*. 2018; 22(12):3892-6.doi: 0.1007/s10461-018-2201-7 [published Online First: 16 June 2018].
[16] Rochat T, Netsi E, Redinger S, Stein A. Parenting and HIV. *Current opinion in psychology*. 2017;15:155-61.doi: 10.1016/j.copsyc.2017.02.019 [published Online First: 22 February 2017].
[17] Sherr L, Cluver LD, Toska E, He E. Differing psychological vulnerabilities among behaviourally and perinatally HIV infected adolescents in South Africa–implications for targeted health service provision. *AIDS care*. 2018;30(sup2):92-101. 10.1080/09540121.2018.1476664 [published Online First: 30 May 2018].
[18] Anderson M, Elam G, Gerver S, et al. "It took a piece of me": Initial responses to a positive HIV diagnosis by Caribbean people in the UK. *AIDS care*. 2010;22(12):1493-8. doi: 10.1080/09540121.2010.482125 [published Online First: 6 December 2018].
[19] Ramirez-Avila L, Regan S, Giddy J, et al. Depressive symptoms and their impact on health-seeking behaviors in newly-diagnosed HIV-infected patients in Durban, South Africa. *AIDS and Behavior*. 2012;16(8):2226-35.doi: 10.1007/s10461-012-0160-y [published Online First: 27 March 2012].
[20] Yah CS, Ndlovu S, Kutywayo A, et al. The prevalence of pregnancy among adolescent girls and young women across the Southern African development community economic hub: A systematic review and meta-analysis. *Health promotion perspectives*. 2020;10(4):325. doi:10.34172/hpp.2020.51 [published Online First: 7 November 2020].
[21] Glassman AL, Silverman R, McQueston K. Adolescent fertility in low-and middle-income countries: effects and solutions. Center for Global Development Working Paper. 2012;295.Available at SSRN: https://ssrn.com/abstract=2102763 or http://dx.doi.org/10.2139/ssrn.2102763 [Date Accessed: 10 September 2021]
[22] Toska E, Laurenzi CA, Roberts KJ et al. Adolescent mothers affected by HIV and their children: a scoping review of evidence and experiences from sub-Saharan Africa. *Global Public Health.* 2020;15(11):1655-73.doi: 10.1080/17441692.2020.1775867 [published Online First: 6 June 2020].
[23] Mullen EM. Mullen scales of early learning. Circle Pines, MN: AGS 1995:58-64.
[24] Bornman J, Romski M, Tonsing K, et al. Adapting and translating the Mullen Scales of Early Learning for the South African context. *South African Journal of Communication Disorders*. 2018;65(1):1-9.doi: 10.4102/sajcd.v65i1.571[published Online First: 8 March 2018].
[25] Koura GK, Boivin MJ, Davidson LL et al. Adaptation of the mullen scales of early learning to Benin setting. *American Journal of Epidemiology*. 2012;175(11):S128-.

For peer review only - http://bmjopen.bmj.com/site/about/guidelines.xhtml

[26] Milosavljevic B, Vellekoop P, Maris H, et al. Adaptation of the Mullen Scales of Early Learning for use among infants aged 5-to 24-months in rural Gambia. Developmental science. 2019;22(5):e12808. doi:10.1111/desc.12808 [published Online First: 9 February 2019].
[27] Durkin MS, Wang W, Shrout PE et al. Evaluating a Ten Questions screen for childhood disability: reliability and internal structure in different cultures. *Journal of Clinical Epidemiology*, 1995;48(5):657-66. doi:10.1016/0895-4356(94)00163-k
[28] Pillay U, Roberts B, Rule S. South African social attitudes: changing times, diverse voices. Cape Town: HSRC press 2006.
[29] Statistics South Africa. *Census 2001: Household Questionnaire*. Pretoria, SA: Statistics SA 2001.
[30] Labadarios D, Steyn NP, Maunder E et al. The national food consumption survey (NFCS): South Africa, 1999. *Public health nutrition*. 2005;8(5):533-43.doi:10.1079/phn2005816 [published Online First: 2 January 2007].
[31] Duong M, Piroth L, Grappin M et al. Evaluation of the Patient Medication Adherence Questionnaire as a tool for self-reported adherence assessment in HIV-infected patients on antiretroviral regimens. *HIV clinical trials*. 2001;2(2):128-35.doi: 10.1310/M3JR-G390-LXCM-F62G [published Online First: 29 January 2015].
[32] Pantelic M, Boyes M, Cluver L, Thabeng M. 'They say HIV is a punishment from god or from ancestors': cross-cultural adaptation and psychometric assessment of an HIV stigma scale for South African adolescents living with HIV (ALHIV-SS). *Child indicators research*. 2018;11(1):207-23. doi:10.1007/s12187-016-9428-5 [published Online First: 23 November 2016].
[33] Kovacs M. Children's depression inventory (CDI). Toronto: Multi-Health System 2003.
[34] Reynolds CR, & Richmond BO. Revised Children's Manifest Anxiety Scales (RCMAS). Los Angeles: Western Psychological Services 1985.
[35] Amaya-Jackson L, McCarthy G, Cherney MS & Newman E. Child PTSD checklist. Durham, NC: Duke University Medical Center 1995.
[36] Sheehan DV, Lecrubier Y, Sheehan KH et al. The Mini-International Neuropsychiatric Interview (MINI): the development and validation of a structured diagnostic psychiatric interview for DSM-IV and ICD-10. *Journal of clinical psychiatry*. 1998;59(20):22-33.
[37] Martin L, Revington N, Seedat S. The 39-item Child Exposure to Community Violence (CECV) Scale: Exploratory factor analysis and relationship to PTSD symptomatology in trauma-exposed children and adolescents. *International journal of behavioral medicine.* 2013;20(4):599-608.doi: 10.1007/s12529-012-9269-7 [published Online First: 2 October 2012].
[38] Finkelhor D, Hamby SL, Ormrod R, Turner H. The Juvenile Victimization Questionnaire: reliability, validity, and national norms. *Child abuse & neglect*. 2005;29(4):383-412.doi: 10.1016/j.chiabu.2004.11.001 [published Online First: 28 April 2005].
[39] Berry JO, Jones WH. The parental stress scale: Initial psychometric evidence. *Journal of social and personal relationships*. 1995 Aug;12(3):463-72.doi: 10.1177/0265407595123009
[40] Sherbourne CD, Stewart AL. The MOS social support survey. *Social science & medicine*. 1991;32(6):705-14.doi: 10.1016/0277-9536(91)90150-B
[41] Casale M. The importance of family and community support for the health of HIV-affected populations in Southern Africa: What do we know and where to from here?. *British Journal of Health Psychology*. 2015;20(1):21-35.doi: 10.1111/bjhp.12127 [first Published Online: 9 December 2014].

For peer review only - http://bmjopen.bmj.com/site/about/guidelines.xhtml

[42] Thomas T, Ahmed Y, Tan M, Grigorenko EL. Cognitive and Educational Interventions for Orphans and Vulnerable Children Affected by HIV/AIDS: A Meta-Analysis. *Child development*. 2021;92(1):7-20. DOI: 10.1111/cdev.13438

[43] Mebrahtu H, Simms V, Mupambireyi Z et al. Effects of parenting classes and economic strengthening for caregivers on the cognition of HIV-exposed infants: a pragmatic cluster randomised controlled trial in rural Zimbabwe. *BMJ global health*. 2019;4(5):e001651.doi: 10.1136/bmjgh-2019-001651[published Online First: 11 September 2019].

[44] Rao N, Sun J, Wong JM et al. Early childhood development and cognitive development in developing countries: A rigorous literature review. http://cerc.edu.hku.hk/wp-content/uploads/ECD-review.pdf [Date accessed: 10 May 2021]

[45] Johnson LF, Dorrington RE, Bradshaw D et al. The effect of educational attainment and other factors on HIV risk in South African women: results from antenatal surveillance, 2000–2005. *Aids*. 2009;23(12):1583-8. doi: 10.1097/QAD.0b013e32832d407e [published Online First: 31 July 2009].

[46] Merville O, Puangmala P, Suksawas P et al. School trajectory disruption among adolescents living with perinatal HIV receiving antiretroviral treatments: a case-control study in Thailand. *BMC public health*. 2021;21(1):1-2.doi: 10.1186/s12889-021-10189-x [published Online First: 21 January 2021].

[47] Toska E, Cluver L, Orkin M, Bains A, Sherr L, Berezin M, Gulaid L. Screening and supporting through schools: Educational experiences and needs of adolescents living with HIV in a South African cohort. *BMC Public Health*. 2019;19(1):1-0. doi: 10.1186/s12889-019-6580-0 [published Online First: 6 March 2019].

For peer review only - http://bmjopen.bmj.com/site/about/guidelines.xhtml

Figure Legend

Figure 1

Flow Chart of Classification by Mode of Maternal HIV Acquisition

For peer review only

For peer review only - http://bmjopen.bmj.com/site/about/guidelines.xhtml

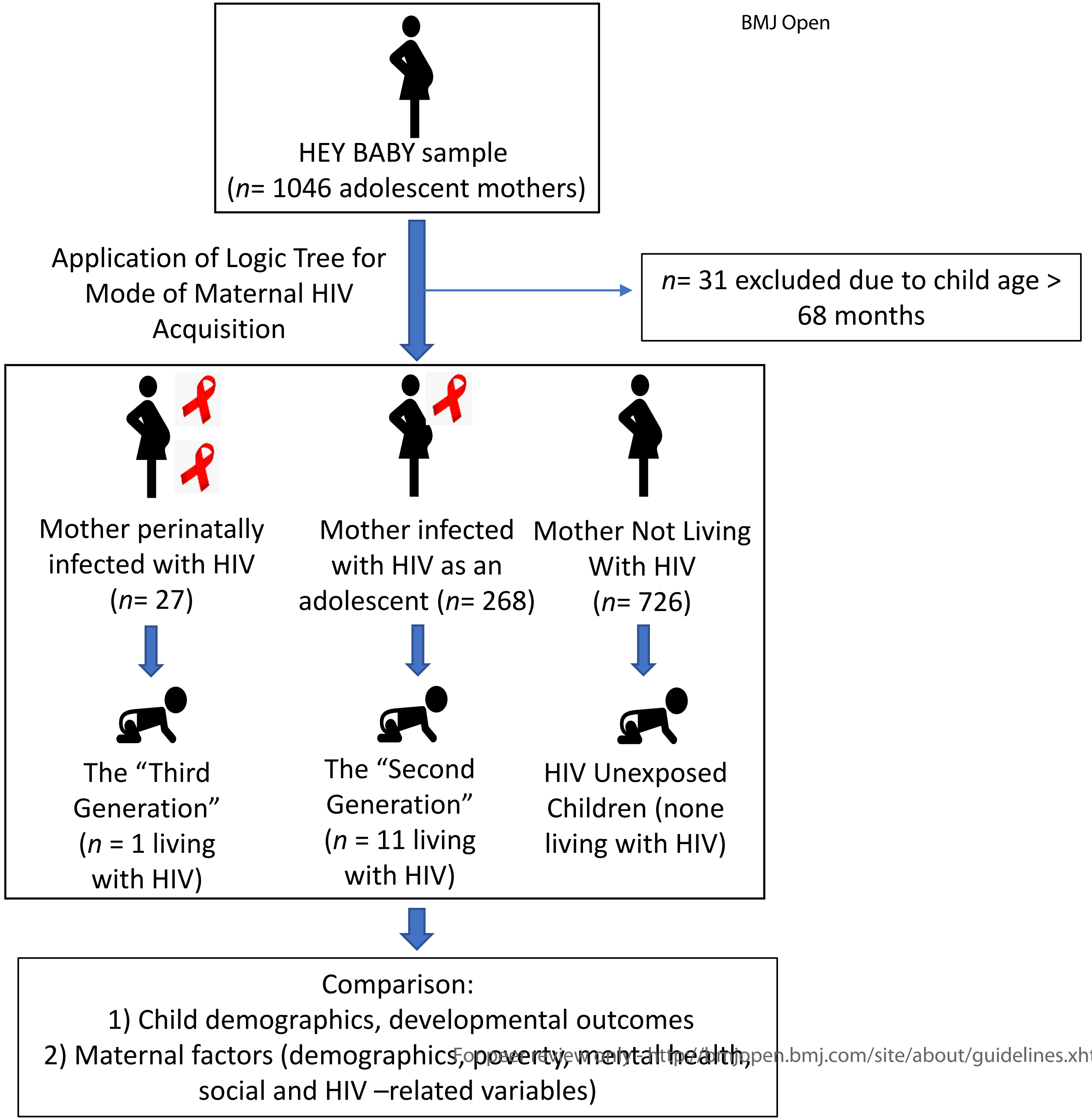

For peer review only - http://bmjopen.bmj.com/site/about/guidelines.xhtml

Appendix 1

Infant (0-68 month) developmental outcomes, according to familial patterns of HIV infection – sample limited to adolescent mothers only

	Overall (*N*=1015)	Third Generation (*n*= 23)	Second Generation (*n*= 229)	No HIV Exposure (*n*= 721)	p-value
**Mullen Scales of Child Development**					
Gross Motor Score*	49.7 (12.5)	54.3 (16. 7)[a]	46.8 (12.7)[b]	50.3 (12.2)	<.001
Visual Reception Score	42.1 (14.2)	44.0 (13.7)	40.4 (14.1)	42.5 (14.2)	.12
Fine Motor Score	43.7(14.7)	45.0 (16.2)	41.3 (15.5)[b]	44.4 (14.3)	.017
Receptive Language Score	47.5 (13.5)	48.5 (14.0)	46.4 (14.9)	47.8 (13.0)	.37
Expressive Language Score	51.6 (13.5)	51.1 (14.0)	51.0 (14.8)	51.8 (13.0)	.71
Composite Score	93.3 (21.3)	95.0 (23.1)	90.6 (22.8)	94.1 (20.7)	.098
**WHO Disability Score- Any Disability**	234 (26.9%)	6 (27.3%)	63 (28.9%)	165 (26.2%)	.73
**Any Days Sick** (past month)	212 (22.1%)	3 (15.0%)	37 (16.2%)	172 (24.2%)	.032

Notes. Group comparisons were conducted using χ^2 tests for categorical variables and univariate analyses of variance for continuous variables. *Gross motor score: n = 831. Mullen subdomain scores range from 20-80, composite scores from 49-155. Tukey's range post hoc tests were undertaken to identify mean differences between groups for continuous variable: [a] Statistically different from the second generation (p<0.05), [b] Statistically different from HIV unexposed group (p<0.05).

For peer review only

BMJ Open

The cognitive characteristics of young children born to young mothers with no, first or second generation HIV acquisition in the Eastern Cape province, South Africa: a cross-sectional study

Journal:	*BMJ Open*
Manuscript ID	bmjopen-2021-058340.R2
Article Type:	Original research
Date Submitted by the Author:	11-Aug-2022

Complete List of Authors:	Sherr, Lorraine; University College London<br>Haag, Katharina; UCL, Institute for Global Health<br>Roberts, Kathryn; UCL, Institute for Global Health<br>Cluver, Lucie; University of Oxford, Centre for Evidence-Based Social Intervention; University of Cape Town, Department of Psychiatry and Mental Health<br>Wittesaele, Camille; University of Oxford, Department of Social Policy & Intervention; London School of Hygiene & Tropical Medicine, Department of Infectious Disease Epidemiology<br>Saliwe, Bongiwe; University of Cape Town, Centre for Social Science Research<br>Tolmay, Janke; University of Cape Town, Centre for Social Science Research<br>Langwenya, Nontokozo; University of Cape Town, Centre for Social Science<br>Jochim, Janina; University of Oxford, Department of Social Policy & Intervention<br>Saal, Wylene; University of Cape Town, Centre for Social Science Research<br>Zhou, Siyanai; University of Cape Town, Centre for Social Science Research<br>Marlow, Marguerite; University of Stellenbosch, Institute for Life Course Health Research, Department of Global Health, Faculty of Medicine and Health Sciences<br>Chen-Charles, Jenny; University of Oxford, Department of Social Policy & Intervention<br>Toska, Elona; University of Cape Town Faculty of Humanities, Centre for Social Science Research & Department of Sociology; University of Oxford, Social Policy and Intervention

**Primary Subject Heading**:	HIV/AIDS
Secondary Subject Heading:	Global health, Mental health
Keywords:	HIV & AIDS < INFECTIOUS DISEASES, Community child health < PAEDIATRICS, EPIDEMIOLOGY, Health policy < HEALTH SERVICES ADMINISTRATION & MANAGEMENT, Developmental neurology &

	neurodisability < PAEDIATRICS

SCHOLARONE™
Manuscripts

I, the Submitting Author has the right to grant and does grant on behalf of all authors of the Work (as defined in the below author licence), an exclusive licence and/or a non-exclusive licence for contributions from authors who are: i) UK Crown employees; ii) where BMJ has agreed a CC-BY licence shall apply, and/or iii) in accordance with the terms applicable for US Federal Government officers or employees acting as part of their official duties; on a worldwide, perpetual, irrevocable, royalty-free basis to BMJ Publishing Group Ltd ("BMJ") its licensees and where the relevant Journal is co-owned by BMJ to the co-owners of the Journal, to publish the Work in this journal and any other BMJ products and to exploit all rights, as set out in our licence.

The Submitting Author accepts and understands that any supply made under these terms is made by BMJ to the Submitting Author unless you are acting as an employee on behalf of your employer or a postgraduate student of an affiliated institution which is paying any applicable article publishing charge ("APC") for Open Access articles. Where the Submitting Author wishes to make the Work available on an Open Access basis (and intends to pay the relevant APC), the terms of reuse of such Open Access shall be governed by a Creative Commons licence – details of these licences and which Creative Commons licence will apply to this Work are set out in our licence referred to above.

Other than as permitted in any relevant BMJ Author's Self Archiving Policies, I confirm this Work has not been accepted for publication elsewhere, is not being considered for publication elsewhere and does not duplicate material already published. I confirm all authors consent to publication of this Work and authorise the granting of this licence.

For peer review only

Title: **The cognitive characteristics of young children born to young mothers with no, first or second generation HIV acquisition in the Eastern Cape province, South Africa: a cross-sectional study**

Authors: Lorraine Sherr[1], Katharina Haag[1], Kathryn Steventon Roberts[1], Lucie Dale Cluver[2,3], Camille Wittesaele2,[4], Bongiwe Saliwe[5], Janke Tolmay[5], Nontokozo Langwenya[5], Janina Jochim[2], Wylene Saal[5], Siyanai Zhou[5], Marguerite Marlow[6], Jenny Chen-Charles[2], Elona Toska[2,5,7]

1 Institute for Global Health, University College London, UK

2 Department of Social Policy & Intervention, University of Oxford, Oxford, UK

3 Department of Psychiatry and Mental Health, University of Cape Town, Cape Town, South Africa

4 Department of Infectious Disease Epidemiology, London School of Hygiene & Tropical Medicine, London, UK

5 Centre for Social Science Research, University of Cape Town, Cape Town, South Africa

6 Institute for Life Course Health Research, Department of Global Health, Faculty of Medicine and Health Sciences, Stellenbosch University, South Africa

7 Department of Sociology, University of Cape Town, Cape Town, South Africa

Corresponding author:

Professor Lorraine Sherr (l.sherr@ucl.ac.uk), University College London, Department of Global Health, Royal Free Hospital Campus, Rowland Hill St, London, NW3 2PF

Tel: +442080168071, ext 68071

Fax: +44 207 794 1224

Word Count: 4668 (excluding title page, abstract, references, figures and tables)

For peer review only - http://bmjopen.bmj.com/site/about/guidelines.xhtml

ABSTRACT

Background: Long-term, inter-generational effects of HIV require investigation. We compared developmental outcomes of the "third generation" (i.e., children of perinatally infected mothers), the "second generation" (i.e., with recently infected mothers) and children of mothers not living with HIV.

Methods: A cross-sectional community sample of *N*=1015 young mothers (12-25 years) and their first children (2- 68 months, 48.2% female), from South Africa's Eastern Cape Province. 71.3% (*n*=724) of children were born to mothers not living with HIV; 2.7% (*n*=27; 1 living with HIV) were third and 26.0% (*n*=264; 11 living with HIV) second generation children. Child scores on the Mullen Scales of Early Learning (MSEL), the WHO Ten Questions Screen for Disability and maternal demographics were compared between groups using $\chi 2$-tests and univariate ANOVAs. Hierarchical linear regressions investigated predictive effects of familial HIV infection patterns on child MSEL composite scores, controlling for demographic and family environment variables.

Results: Second generation children performed poorer on gross (*M*=47.0, *SD*=13.1) and fine motor functioning (*M*=41.4, *SD*=15.2) and the MSEL composite score (*M*=90.6, *SD*=23.0) than children with non-infected mothers (gross motor: *M*=50.4, *SD*=12.3; fine motor: *M*=44.4, *SD*=14.1; composite score: *M*=94.1, *SD*=20.7). The third generation performed at similar levels to non-exposed children (gross motor: *M*=52.4, *SD*=16.1; fine motor: *M*=44.3, *SD*=16.1, composite score: *M*=94.7, *SD*=22.2), though analyses were underpowered for definite conclusions. Hierarchical regressions suggest marginal predictive effects of being second generation child compared to having a mother not living with HIV (*B*=-3.3, *CI*=-6.8; .1) on MSEL total scores, and non-significant predictive effects of being a third generation child (*B*= 1.1, *CI* = -7.5: 9.7) when controlling for covariates. No group differences were found for

For peer review only - http://bmjopen.bmj.com/site/about/guidelines.xhtml

disability rates (26.9% third generation, 27.7% second generation, 26.2% non-exposed; χ^2 = .02, p = .90).

Conclusion: Recently infected mothers and their children may struggle due to the disruptiveness of new HIV diagnoses and incomplete access to care/ support during pregnancy and early motherhood. Longstanding familial HIV infection may facilitate care pathways and coping, explaining similar cognitive development among not exposed and third generation children. Targeted intervention and fast-tracking into services may improve maternal mental health and socioeconomic support.

Keywords: HIV; adolescent mothers, infants, cognitive development, mental health, environmental factors

For peer review only - http://bmjopen.bmj.com/site/about/guidelines.xhtml

STRENGTHS AND LIMITATIONS OF THIS STUDY

- To our knowledge, this is the first study to investigate developmental outcomes and life circumstances of the "third generation", i.e. children with a perinatally infected mothers.
- A particular strength is the comparison to both children of the "second generation" (i.e., with recently infected mothers) and children not affected by HIV but living in similar high-risk environments characterized by poverty and deprivation.
- While we found that children of the third generation perform equally well or better than children not directly exposed to HIV, the sample of the former was small, limiting our power to draw definite conclusion.
- Our sample consisted predominantly of young mothers. While both rates of HIV infection and young pregnancy may be compounded in deprived areas, the current findings need to be replicated in a more representative sample.

For peer review only

INTRODUCTION

Approximately 7.5 million people in South Africa were living with HIV in 2020, including 360,000 adolescents aged 10-19 years.[1] Generally, HIV infections have been linked to higher rates of morbidity, stigma, poverty, and other adverse outcomes.[2, 3] While rates of perinatal HIV infection are declining, due to improved screening and treatment options [4-6], historical exposure means that an increasing number of perinatally-infected children are now reaching adolescence and young adulthood and are becoming parents themselves. There is a need to study their children's developmental outcomes, in order to devise effective support for these families.

To knowledge of the authors, to date, there have been limited studies focusing on this "third generation", i.e., children who have adolescent mother perinatally infected by HIV. Most designs do not differentiate between mothers who were recently or perinatally infected and instead focus on comparing HIV infected, HIV exposed and uninfected, and HIV unexposed children.[7-9] Findings suggest that the former two groups may show developmental sequelae (including cognitive and motor), which can be explained by exposure to the virus (in utero) and ARTs as well as environmental factors associated with HIV infection (e.g., prematurity, poor parental mental health and substance use, stigma, caretaker changes, food insecurity).[10, 11] However, specific knowledge on the third generation (i.e., children of mothers perinatally infected with HIV) is still extremely limited. The few existing studies focus mainly on maternal characteristics (e.g., ART adherence, age at pregnancy) and child infection status.[12] To our knowledge, no studies have yet investigated the effects of intergenerational HIV infection on child outcomes independent of child HIV status, and also in comparison to a group of children without familial HIV exposure, but who live in the same high-risk environment.

Of note, previous research on adolescents who were perinatally infected (potential mothers of the third generation) versus recently infected (potential mothers of the second

For peer review only - http://bmjopen.bmj.com/site/about/guidelines.xhtml

generation) indicates that they may be facing different types of life challenges that could affect their parenting in divergent ways. Those perinatally infected tend to have experienced more chronic adversity, such as parental and/or sibling illness and death, caretaking responsibilities, poorer parenting and compromised parent-child relationships, poor wider family and community support, and compounded internalized and associative stigma, all of which may increase the risk for lower well-being and higher substance use.[2, 4, 13-17] Those recently infected, in contrast, have been proposed to more likely be members of marginalized groups (e.g., drug users, adolescents with street involvement), to struggle with the impacts of their recent diagnosis, including biographical disruptions and reduced hope for the future, and to have difficulties with accessing and engaging in care. All of these issues may be compounded by mental health problems including anxiety, depression, substance use, and internalized stigma.[14, 17-19]

The current study was set up to explore the development of young children of adolescent and young adult mothers living in the Eastern Cape of South Africa, with a specific aim to gain a better understanding of third- and second-generation HIV effects. HIV infections often tend to cluster in disadvantaged environments, leading to disproportionally affected communities, where risks of initial HIV infection and HIV being passed on across generations are heightened. These communities are often also affected by high adolescent pregnancy rates,[20] which are associated with a range of risks for mother and child such as poor educational prospects, exposure to poverty and violence, and poor developmental outcomes.[21,22] Furthermore, they can also be a marker for adolescent sexual risk behaviours, and there may be a syndemic occurrence between HIV and adolescent parenthood risk among young women in sub-Saharan Africa.[22] The current sample, which predominantly consists of adolescent mothers (age 19 or under) thus provides an unique opportunity to study the compounded effects of HIV and young pregnancy.

For peer review only - http://bmjopen.bmj.com/site/about/guidelines.xhtml

We had two main analysis aims: 1) to compare child developmental scores (fine and gross motor, visual reception, expressive and receptive language skills) at ages 2-68 months, independent of child HIV status, in children of mothers who were perinatally infected (third generation), recently infected through sexual exposure (second generation) and non-infected but living in the same high-risk environment) and 2) to investigate how mothers of these groups may differ in terms of their socio-economic status, well-being, and social support, all of which can shape the environment a child grows up in.

METHODS

Participants

Data utilised within these analyses originate from the "Helping Empower Youth Brought up in Adversity with their Babies and Young children" (HEY BABY) study. The study was conducted in rural and peri-urban health districts of South Africa's Eastern Cape and aimed at investigating the effects of adolescent motherhood, as well as intergenerational effects of HIV exposure. 1046 adolescent and young adult mothers (10-25 years) with at least one living child were interviewed between March 2018 and July 2019. The required sample size was estimated based on expected effect sizes for key outcomes. Participants were partially recruited from a previous study (*n*= 159: any young mothers included), as well as through six parallel sampling strategies developed in cooperation with local experts and an adolescent mother advisory group to ensure representativeness (*n*= 887: only adolescent mothers). This comprised sampling through 73 known health facilities within the districts, 43 secondary schools, 9 maternity units, and referrals by service providers, social workers, and adolescent mothers themselves. 95%-98% of eligible mothers from each recruitment channel were successfully enrolled into the study. The current analyses were limited to data on first childof the young mother only, and children aged 68 months or younger who were within the normed range for

For peer review only - http://bmjopen.bmj.com/site/about/guidelines.xhtml

For peer review only

the Mullen Scales (n= 31 excluded based on this age restriction), limiting the sample to n = 1015. We also performed sensitivity analyses including adolescent mothers (age at pregnancy ≤ 19, n= 972) only for the key outcomes (see Appendix 1), as these mothers had been the main recruitment focus for the study.

Patient and Public Involvement

The study team were advised on recruitment methods by adolescent mothers, whose suggestions were included in the study protocol. Furthermore, the team has been working with two Teen Advisory groups in the Eastern and Western Cape of South Africa, who were involved in piloting the study. Feedback was incorporated to improve relevance and acceptability of the questionnaire and the research procedure. Finally, through the engagement of community leaders, it was ensured that recruitment strategies were effective, sensitive to participant's circumstances and minimised stigma risks.

Procedure

The dataset analysed combined four data sources. Firstly, all adolescent mother participants completed a detailed study questionnaire relating to socio-demographic characteristics, sexual and reproductive health, physical and mental health, relationships, and social support. Secondly, they completed an adolescent parent questionnaire, which collected data on maternal and child health, child development, the father of the child, and maternal factors including social support, the parenting experience, and violence exposure. Thirdly, cognitive assessments of the children were performed by a trained administrator, using the Mullen Scales of Early Learning. Finally, details from the child's medical records (*Road to Health Booklet*) were also included in the database. All participants provided written consent, and interviews were conducted in the language of their choice (i.e., English or isiXhosa). Regardless of participation, all adolescents received a small 'snack pack' containing a snack

For peer review only - http://bmjopen.bmj.com/site/about/guidelines.xhtml

and juice during the interview, and a small 'thank you pack', with personal products such as toothpaste and a toothbrush. Items included in these packs were selected by the adolescent advisory group as preferable and appropriate. New mothers also received a 'baby pack', the contents of which were also chosen by an adolescent advisory group, and included nappies and baby cream.

Measures

Mode of maternal HIV acquisition

Mode of maternal HIV acquisition (perinatal versus recent) was assessed through an algorithm, given that the study was community-based and not linked to clinical testing data. Accordingly, it was derived using a logic tree based on clinical and fieldwork experiences .[17] The algorithm allowed for categorisation according to self-report, age of ART initiation, and parental death information (Tolmay, Saal et al., in preparation). For the current analyses, we compared the following three groups: children of the third generation (mother perinatally infected), children of the second generation (mother recently infected), and children of mothers not living with HIV (see Figure 1). Child HIV status was not taken into account in these classifications, since absolute numbers were low (n = 12 based on maternal self-reported data), with only one of these children being in the third generation. Thus, we were underpowered to study these children separately. We decided however to retain them in the analyses since they are relevant members of the second and third generations respectively.

Child developmental outcomes

Child developmental outcomes were assessed using the Mullen Scales of Early Development (MSEL).[23] The MSEL is normed for children aged 0 to 68 months (USA) and

For peer review only - http://bmjopen.bmj.com/site/about/guidelines.xhtml

assesses child performance across five domains: gross motor (only for age <39 months), fine motor, visual reception, receptive and expressive language (score range: 20-80). A composite score (score range: 49-155) can be derived, and the scales have been validated for use in Sub-Saharan Africa.[24-26] Child disability status was assessed using the WHO Ten Questions Screen for Disability.[27] This measure can be applied to detect common disabilities (hearing, visual, physical, speech, mental, and epilepsy) in children. A score indicating any disability ("yes" across any of the 10 items) was derived for the current analyses.

Maternal variables

Adolescent mothers were compared on various variables, including demographic factors (maternal age, maternal and paternal age at pregnancy), child feeding method used (formula, breastfeeding, combined, other), maternal school progression (self-report: repeated at least one school grade), poverty (number of the eight socially perceived necessities for children the family had access to, [28] household access to any government cash transfers (child support, foster child, pension, disability, or care dependency grant, measured via South Africa Census item [29]), food security (number of days there was not enough food for the household in the past seven days [30]), full ART adherence over the past seven days (Patient Medication Adherence Questionnaire [31]), any HIV clinic appointments missed in the past year, extent of HIV-related stigma (Adolescents Living with HIV Stigma Scale [32]), depressive symptoms in the past two weeks (Child Depression Inventory-Short Form [33])), anxiety symptoms in the past month (Revised Children's Manifest Anxiety Scale [34]), PTSD symptoms in the past month (Child PTSD Checklist-Short Form [35]), suicidality symptoms during the past month (Mini International Psychiatric Interview for Children and Adolescents-Suicidality and Self-Harm Subscale [36]), extent of community violence exposure in the past year (item from the Child Exposure to Community Violence Checklist [37]), intimate partner violence exposure in the past month (Juvenile Victimization Questionnaire [38]), current

For peer review only - http://bmjopen.bmj.com/site/about/guidelines.xhtml

parenting stress (Parental Stress Scale [39]) and amount of social support received (adjusted version of the Medical Outcomes Study Social Support Survey [40], previously used in the South African context [41]).

Statistical analyses

All analyses were conducted using STATA 16 SE. Firstly, child and adolescent mother descriptive information for the three groups of interest (children of the third generation, children of the second generation, and HIV-unexposed children) was provided. Next, child developmental outcomes and maternal factors surrounding socio-economic status, HIV-related variables (only HIV-affected mothers), mental health and social environment were compared between groups, using $\chi2$ tests and univariate analyses of variance (ANOVA) as appropriate. For continuous variables, Tukey's range tests were used to conduct post-hoc group comparisons. Effects at $p<.05$ were considered relevant. Finally, a hierarchical regression model was run, with the child composite score on the Mullen scales as a key outcome. In a first step, child familial HIV exposure (third generation, second generation, HIV-unexposed) was added as a predictor. In a second step, we included relevant control variables that were found to be associated with the predictor or outcome variables in univariate analyses or were identified as relevant within the literature to see whether child familial HIV exposure would remain a relevant predictor.

RESULTS

Descriptive variables

After applying the logic tree for mode of maternal HIV acquisition, 27 mothers (2.7%) were classified as perinatally infected, 264 (26.0%) as recently/sexually infected, and 724 (71.3%) as not living with HIV. Table 1 presents adolescent mother and child sample characteristics, split according to whether the child was in the third generation, the second

For peer review only - http://bmjopen.bmj.com/site/about/guidelines.xhtml

generation, or not exposed to HIV. There were no significant group differences in child sex, HIV status, and creche attendance; however, children of the second generation were on average older than those without HIV exposure (*Diff*= 6.69, *95% CI*= 4.26; 9.12), likely due to maternal age differences (see below). No significant age differences were found between children of the third generation and the other two groups (*Diff*= 2.63, *95% CI*= -4.20; 9.46). Formula (without breastfeeding) use was the most common feeding method during the first six months of the child's life for mothers of the third generation (63.0%). For mothers of the second generation, formula use (only) and breastfeeding (only) were similarly common (35.0% and 38.4%), while the majority of mothers of HIV-unexposed children opted for a mix of both (40.9%).

Similar to their children, mothers of the second generation (*M*= 19.7 years) were significantly older than those of third generation (*M*= 18.7 years), and the latter were older than mothers of HIV-unexposed children (*M*= 17.7 years) (*F*= 148.01, *p*<.001). Furthermore, mothers of the second and third generation were significantly older at pregnancy with the participating child (*M*= 17.8 and *M*= 17.3 versus *M*= 16.3 years respectively; *F*= 89.23, *p* <.001) compared to mothers of HIV-unexposed children. Fathers of the second generation (*M*= 23.2 years) were on average older at childbirth than those of the third generation and HIV-unexposed children (*M*= 21.3 and *M*= 20.4 years respectively, *F*= 49.08, *p* <.001). There was no difference between groups regarding whether the mother lived in informal housing or a rural area, though mothers unaffected by HIV were marginally less likely to be in a relationship (64.3%, versus 77.8%% for the third generation and 71.5% for the second generation; χ^2= 6.52; *p*= .038). Mothers of the second generation were more likely to have repeated a school grade (69.3%), versus 61.5% for mothers of the third generation and 57.9% for mothers of HIV-unexposed children (χ^2= 9.91, *p*= .007).

Table 1

For peer review only - http://bmjopen.bmj.com/site/about/guidelines.xhtml

Sociodemographic characteristics of adolescent mothers and their children according to familial patterns of HIV infection

	Overall (*N*=1015)	Third Generation (*n*= 27)	Second Generation (*n*= 264)	No HIV Exposure (*n*= 724)	p-value
**Child Variables**					
Age in months (M, SD)	18.7 (14.7)	20.9 (16.7)	23.5 (16.4)[b]	16.8 (13.5)	<.001
Sex (1= female)	489 (48.2%)	16 (59.3%)	123 (48.2%)	350 (48.3%)	.46
HIV Status	12 (5.0%)	1 (9.1%)	11 (10.0%)	-	.92
Feeding					
Breastfeeding And Formula	347 (34.2%)	4 (14.8%)	47 (17.9%)	296 (40.9%)	<.001
Baby Formula Only	318 (31.4%)	17 (63.0%)	92 (35.0%)	209 (28.9%)	
Breastfeeding Only	258 (25.4%)	5 (18.5%)	101 (38.4%)	152 (21.0%)	
Other Milk or Water	91 (9.0%)	1 (3.7%)	23 (8.8%)	67 (9.3%)	
Creche Attendance	304 (29.8%)	8 (29.6%)	80 (30.4%)	216 (29.8%)	.98
**Maternal Variables**					
Age in years (M, SD)	18.24 (1.8)	18.7 (1.6)[a],[b]	19.7 (2.0)[b]	17.7 (1.5)	<.001
Age at Pregnancy With The Participating Child (years; M, SD)	16.8 (1.7)	17.3 (2.0)	17.8 (1.9)	16.3 (1.4)	<.001
Paternal Age at Pregnancy (years, M, SD)	21.1 (3. 9)	21.3 (4.3)[a]	23.2 (4.08)[b]	20.4 (3.5)	<.001
Informal Housing	217 (21.9%)	6 (23.1%)	60 (23.6%)	151 (21.2%)	.76
Living in Rural Area	293 (28.9%)	8 (29.6%)	63(23.9%)	222 (30.7%)	.11
Any School Grade Repeated	581 (60.8%)	16 (61.5%)	169 (69.3%)	396 (57.8%)	.007
Current Relationship	670 (66.5%)	21 (77.8%)	186 (71.8%)	463 (64.2%)	.038

Notes. Group comparisons were conducted using $\chi2$ tests for categorical variables and univariate analyses of variance for continuous variables. Tukey 's range post hoc tests were undertaken to identify mean differences between groups for continuous variables: [a] Statistically different from the second generation (p<0.05), [b] Statistically different from HIV unexposed group (p<0.05). Underlying *N*s vary depending on missing values (*N*= 933 to *N*= 1015).

Child developmental outcomes

Table 2 presents child developmental outcomes according to familial patterns of HIV infection. For the Mullen Scales of Early Child Development, we found that the third generation had the highest gross motor scores (*M*= 52.4, *SD*= 16.1), ahead of children not

For peer review only - http://bmjopen.bmj.com/site/about/guidelines.xhtml

exposed to HIV (*M*= 50.4, *SD* = 12.3) and the second generation (*M*= 47.0, *SD* = 13.1; *F* = 6.50, *p*= .001). However, due to low power resulting from the small sample of children in the third generation, Tukey's range post hoc tests only identified a significant difference between the latter two groups (*Diff*= 3.41, *95% CI*= 1.08; 5.73). Children of the second generation (*M*= 41.4, *SD*= 15.2) exhibited poorer fine motor scores than both HIV non-exposed children (*M*= 44.4, *SD*= 14.1) and children of the third generation (*M*= 44.3, *SD*= 16.1; *F*= 4.26, *p*= .014), though post-hoc tests again only indicated significant differences between the former two groups (*Diff*= 3.04, *95%CI*= .59: 5.49). No significant differences were found on the visual reception, receptive, and expressive language subscales,though scores for the second generation were consistently lowest, while children of the third generation tended to do as well or even better than non-exposed children. In terms of the composite scores, children of the second generation performed marginally lower (*M*= 90.6, *SD*= 23.0) than those not exposed to HIV (*M*= 94.1, *SD*= 20.7; *p*= .055) in post-hoc tests (*Diff*= 3.55, *95%CI*= -.05; 7.16). Differences of the former to third generation (*M*= 94.7, *SD*= 22.2) were not significant in post-hoc tests (*p*= .66), despite the scores of the third generation being approximately equal to those of non-infected children (overall model: *F*= 2.72 *p*= .066). Of note, all groups performed lower than expected compared to the US reference population based on which the Mullen Scales were developed (*M*= 100.0 for the composite score; *p*<.001). No significant group differences were found for presence of any disability (χ^2 = .02, *p*= .90). Finally, the rate of children who had been sick at least one day during the past month was lowest for the second generation (15.6%), with increasing rates towards the third generation (17.4%) and those not exposed to HIV (24.2%) (χ^2 = 8.62, *p* = .013). When limiting the sample to adolescent mothers (age <=19) only, the same magnitude and directions of findings were obtained (see Appendix 1).

Table 2

Infant (0-68 month) developmental outcomes, according to familial patterns of HIV infection

For peer review only - http://bmjopen.bmj.com/site/about/guidelines.xhtml

	Overall (N=1015)	Third Generation (n= 27)	Second Generation (n= 264)	No HIV Exposure (n= 724)	p-value
**Mullen Scales of Child Development** *(M, SD)*					
Gross Motor Score*	49.6 (12.7)	52.4(16.1)	47.0 (13.1)[b]	50.4 (12.3)	.002
Visual Reception Score	42.1 (14.2)	43.9 (14.7)	40.7 (14.3)	42.6 (14.2)	.14
Fine Motor Score	43.6 (14.6)	44.3 (16.1)	41.4 (15.2)[b]	44.4 (14.2)	.014
Receptive Language Score	47.4 (13.6)	47.7 (13.8)	46.1 (15.0)	47.8 (13.0)	.21
Expressive Language Score	51.6 (13.5)	51.7 (13.4)	50.7 (14.8)	51.9 (13.0)	.48
Composite Score	93.2 (21.4)	94.6 (23.4)	90.6 (23.0)[b]	94.1 (20.7)	.066
**WHO Disability Score- Any Disability** *(N, %)*	242 (26.7%)	7 (26.9%)	69 (27.7%)	166 (26.2%)	.90
**Any Days Sick** (past month) *(N, %)*	218 (21.8%)	4 (17.4%)	41 (15.6%)	173 (24.2%)	.013

Notes. Group comparisons were conducted using χ^2 tests for categorical variables and univariate analyses of variance for continuous variables. *Gross motor score: n = 831. Mullen subdomain scores range from 20-80, composite scores from 49-155. Tukey's range post hoc tests were undertaken to identify mean differences between groups for continuous variable: [a] Statistically different from the second generation (p<0.05), [b] Statistically different from HIV unexposed group (p<0.05).

Maternal characteristics for the three subgroups

Mothers of the second and third generations had on average fewer of the eight necessities available (F= 10.11, p< .001) (see Table 3); post-hoc tests identified significant differences for second generation (p< .001) and non- significant differences for third generation mothers (p = .060) compared to those not living with HIV. Mothers of the second and third generations were also more likely to receive household cash grants (χ^2= 8.10; p= .017). However, mothers of the third generation had the lowest average days on which they had no food available for the child in the past week, followed by mothers not living with HIV, and then recently infected mothers, though the overall model was only close to significant (F=2.73; p = .066). Mothers of the second and third generations did not differ regarding ART adherence, clinic appointments missed, and HIV-related stigma (p= .69-.95). Both groups were about half

For peer review only - http://bmjopen.bmj.com/site/about/guidelines.xhtml

as likely to have been in excellent physical health in the past twelve months as mothers of non-exposed children (χ^2= 108.46; p <.001).

Mental health symptom scores were overall low, and no differences were found between groups in terms of anxiety (F= .89, p= .41) and only marginal differences for PTSD, with mothers from the second generation scoring higher than bother other groups (F= 2.38 p = .093). Mothers of the second generation also had the highest depression scores (F= 6.12, p = .002), with post-hoc tests indicating significant differences to those not HIV exposed (p = .005) and marginal differences to mothers of third generation (p = .053). Second generation mothers also scored substantially higher than both other groups on suicidality levels (F= 4.11, p = .017), though due to low power, only significant differences to mothers not living with HIV were identified in post-hoc tests (p = .017). Finally, no group differences were detected for exposure to intimate partner and community violence or parenting stress (p= .39-.97). However, mothers of the third generation indicated the lowest levels of social support, followed by the second generation and then mothers of non-exposed children (F= 3.98, p= .019), though post-hoc tests indicated only close to significant differences between mothers of the third generation and those of HIV-unexposed children (p = .061).

Table 3

Comparison of maternal life circumstances, according to familial patterns of HIV infection

	Overall (*N*=1015)	Third Generation (*n*= 27)	Second Generation (*n*= 264)	No HIV Exposure (*n*= 724)	p-value
**Poverty and Social Protection**					
Number of Eight Necessities Available	5.2 (2.2)	4.4 (2.7)	4.8 (2.3)^{b}	5.4 (2.1)	<.001
Any Household Grant	944 (92.5%)	26 (96.3%)	254 (96.2%)	659 (91.1%)	.017
Days Insufficient Food for Child (past week)	.3 (1.1)	.0 (.2)	.4 (1.2)	.3 (1.0)	.07

For peer review only - http://bmjopen.bmj.com/site/about/guidelines.xhtml

**HIV-associated variables**					
Full ART Adherence past 7 days	213 (75.8%)	19 (79.2%)	194 (75.5%)	-	.69
Any Clinic Appointments Missed past month	10 (4.1%)	1 (4.6%)	9 (4.1%)	-	.92
Any Stigma	56 (20.3%)	5 (20.8%)	51 (20.2%)	-	.95
**Physical and Mental Health**					
Excellent Physical Health past 12 months	598 (58.6%)	9 (33.3%)	88 (33.3%)	499 (68.9%)	<.001
Depression Score past 2 weeks (0-20)	.7 (1.4)	.3 (.7)	1.0 (1.7)[b]	.6 (1.3)	.002
Anxiety Score past month (0-14)	.8 (1.9)	.7 (1.6)	.6 (1.9)	.8 (1.9)	.41
PTSD Score past month (0-36)	3.0 (5.6)	2.0 (4.9)	3.6 (5.6)	2.9 (4.9)	.09
Suicidality Score past month (0-5)	.2 (.8)	.1 (.3)	.3 (1.1)[b]	.1 (.7)	.017
**Social and Environmental Factors**					
Parenting Stress Score (18-90)	24.8 (5.8)	24.7 (4.8)	24.9 (5.6)	24.8 (5.8)	.97
Social Support Score (0-14)	13.4 (2.04)	12.7 (2.5)	13.2(2.3)	13.5 (1.9)	.019
Extent of Intimate Partner Violence Exposure (0-4)	.1 (.6)	.1 (.4)	.2 (.7)	.1 (.5)	.39
Extent of Community Violence Exposure (0-4)	.3 (.8)	.2 (.6)	.3 (.8)	.3 (.8)	.79

Notes. Group comparisons were conducted using χ^2 tests for categorical variables and univariate analysis of variance for continuous variables. Tukey's range post hoc tests were undertaken to identify mean differences between groups for continuous variable: [a] Statistically different from the second generation (p<0.05), [b] Statistically different from HIV unexposed group (p<0.05). Underlying Ns vary depending on missing values (N= 950 to N= 1015; N = 828 for Intimate Partner Violence- low response rates may be due to the sensitive topic).

Regression models for predicting child cognitive development composite score

Table 4 shows the results of the hierarchical linear regression model predicting the child composite score on the Mullen Scales. In step 1, compared to the baseline class of children not

For peer review only

exposed to HIV, being a child of the second generation was associated with a lower developmental score (*p*= .021); no predictive effect was found for being a child of the third generation (*p* = .91; model: *F*(2,1012) = 2.72, *p* = .066). The former association was retained at a marginal level in the adjusted model in step 2 (*p* = .059; overall model: *F*(8,889) = 4.08, *p* <.001), with mothers having repeated a school grade additionally being predictive of poorer outcomes, and higher maternal age at birth exerting a protective influence. No predictive effects were found for maternal depression, social support, number of necessities, and food insecurity. Overall, however, the amount of variance explained by both models was relatively low.

Table 4

Multiple regression analysis predicting child developmental score from familial patterns of HIV infection in step 1, with step 2 controlling for relevant child and maternal variables

	B (95% CI)	β	p-value
**Step 1: Unadjusted model**			
Mother not living with HIV (Ref.)	-		
Mother perinatally infected	.46 (-7.77; 8.69)	.00	.91
Mother recently infected	-3.5 (-6.57; -.53)	-.07	.021
Adjusted R2	.003		
**Step 2: Adjusted model**			
Mother not living with HIV (Ref.)	-		
Mother perinatally infected	1.06 (-7.51; 9.65)	.01	.81
Mother recently infected	-3.36 (-6.85; .12)	-.07	.059
Maternal Depression Score	-.45 (-1.46; .57)	-.03	.39
Maternal Social Support	.21 (-.47; .89)	.02	.54
Number of Necessities	.36 (-.30; .1.03)	.04	.29
Maternal Age at Pregnancy	1.10 (.21; 1.99)	.09	.016
Mother repeated any school grade	-5.98 (-8.87; -3.07)	-.14	<.001
Number of days insufficient food for child	-.32 (-1.66; 1.01)	-.02	.64
Adjusted R2	.027		

Note. Step 2 (adjusted model) based on *N* = 898, due to missing data on the control variables. B = unstandardized regression weights. β = standardized regression weights.

DISCUSSION

For peer review only - http://bmjopen.bmj.com/site/about/guidelines.xhtml

In this sample of young mothers and their infants living in South Africa, we found that children of the second generation (i.e., with mothers recently infected with HIV) scored significantly lower on gross motor and fine motor functioning. For the overall composite score they tended to show lower scores as well than children not exposed to HIV. Children of the third generation (i.e., of perinatally infected mothers) appeared score similarly or even higher than children not exposed to HIV across all domains. However, the comparatively small sample size in this group makes it challenging to draw definite conclusions. Mothers also differed in their life circumstances, with mothers of the second generation showing higher rates of depression and suicidality symptoms, as well as lower food security, while mothers of the third generation indicated lower social support. No differences were found on adherence and clinical attendance variables.

Any interpretation of the current data warrants caution, due to low power resulting from the small sample size of the third-generation group. Still, our data appear to provide a tentative first indication that children of the third generation could be doing similarly well as those not exposed to familial HIV across most developmental domains. This would be promising and may indicate that families affected by HIV are in the long-term profiting from efforts to provide social protection, links to services and antiretroviral treatment, which could prevent chronic adversity from being perpetuated. However, it remains to be seen whether such benefits are sustained, with studies in children living with HIV suggesting positive early developmental outcomes, but declines with age [26; Roberts et al., in preparation]. While the aim is to ultimately prevent perinatal HIV infections, larger studies of already infected young adults and their children may provide further insights into the development and needs of children of the third generation.

Children of the second generation, on the other hand, tended to do poorer across several domains, an effect that was marginally retained when controlling for maternal mental health,

For peer review only - http://bmjopen.bmj.com/site/about/guidelines.xhtml

social support, and socio-demographic factors. The shock of maternal recent HIV diagnosis may have placed a major burden on these families, compounded by risk drivers which may have accounted for the HIV exposure in the first place, such as economic and food insecurity, or mental health struggles. This indicates that affected families may profit from additional support, including early cognitive interventions for infants [10, 42] and emotional and social support for mothers at the time of and following diagnosis. Furthermore, these mothers may need to be fast-tracked into services, given the particular double vulnerability of HIV and teen pregnancy.

Of note, the overall child sample underperformed somewhat compared to the American normative sample [23] and a similar group of children in Zimbabwe, who had caregivers living with HIV.[43] Although the level of disability did not differ across the three groups, 26% of the total sample scored for any disability. Targeted interventions could improve developmental outcomes,[44] and adapted disability services may be needed for many of these young children. The results from the current study indicate that addressing factors such as young pregnancy or poor maternal education could be of particular benefit. Maternal school progression delay in particular has been shown to be a risk factor for both early motherhood and HIV infection, as well as an outcome of perinatal HIV infection.[45, 46, 47] Therefore, it will be important to study this factor as a risk marker for child poor developmental outcomes. However, it is important to note that our model only explained a low amount of variance overall, suggesting that other factors may be key for improving child developmental outcomes.

It is important to note though that all adolescent mothers and their children in the current sample had a number of challenges to contend with. Only 24.6% attended creche (no difference by group). The mean age for first childbirth was 16.76 years, with the teens living with HIV being on average about a year older. Over 90% were in receipt of some form of household grant. This highlights the compounded disadvantages that adolescent/young mothers living in

For peer review only - http://bmjopen.bmj.com/site/about/guidelines.xhtml

marginalized areas may face, and the urgent need for support and intervention [22]. In accordance with previous literature, [16] adolescents faced different challenges depending on whether they were perinatally or recently infected (lower social support in the third generation, versus food insecurity and mental-health related difficulties in the second generation). This suggests second generation mothers may require additional support in the form of social protection and access to services [48, 49]. Mothers of the third generation, on the other hand, appear to have adapted to some degree, but may profit from initiatives to reduce chronic stigma and improve social support in an environment that has been long-term affected by HIV [50].

The current study has several limitations. Firstly, the data collected are cross-sectional in nature, and thus do not allow any deductions about temporal relations or causality. Furthermore, they stem solely from South Africa and sampling focused predominantly on a particular group (adolescent mothers from disadvantaged environments) and was thus not completely random, potentially limiting the generalizability of our findings to other countries and contexts. Thirdly, the majority of maternal data were collected through self-report, allowing the possibility for bias. Particularly maternal mental health difficulty rates were overall very low in this sample, leading to the question of whether this is due to high resilience or measurement difficulties. Fourth, as the study was community-based, and clinic records were wanting, an algorithm was required to categorise perinatal infection. Fifth, the standardised developmental inventory has been validated and used widely in Sub-Africa (e.g., Zimbabwe, Zambia, South Africa), but is set against USA norms, and local inventories with local references groups may be more sensitive for future studies. Sixth, while our sample predominantly comprised adolescent mothers, some older mothers up to age 25 whom we had historical data for were also included in our analyses to increase analytical power. This limited our ability to draw conclusions for adolescent mothers specifically. Furthermore, our study may not generalise to older mothers and data on fathers could help to further enhance our

For peer review only - http://bmjopen.bmj.com/site/about/guidelines.xhtml

understanding. Yet it seems important that real life multiple challenges (i.e., compounded effects of HIV exposure and young motherhood) should be appreciated, and these insights applied to wider groups. Finally, the group of children of the third generation was relatively small, which -while encouraging giving HIV preventative efforts- limited our power to investigate group differences and to perform more complex analyses, such as mediation or path models, to better understand pathways for intervention. Future studies may aim at specifically recruiting a larger sample of third generation children to replicate and extend our findings. The effects of child HIV on cognitive outcomes in the overall sample are explored elsewhere (Roberts et al., in preparation).

CONCLUSION

The current paper takes an important first look at the children of the "third generation", giving an impression of their developmental status and life circumstances. The data tentatively suggests that young mothers who have lived in families with HIV for many years learn to understand and accommodate the situation, have worked out pathways to support, and may not have to deal with the shock of HIV diagnosis at the time of pregnancy. Newly infected adolescents may not have the same level of family support and understanding, and they may be coming to terms with their diagnosis at the same time as adjusting to motherhood. For all children, access to early childhood programmes was low, despite them having a good evidence base for enhancing child outcomes. Policies around such provisions should be incorporated for these groups. Future studies will benefit from longitudinal data, including a larger group of children of mothers perinatally infected with HIV, to better understand child cognitive development of the second and third generation, and how this is influenced by maternal factors. While the long-term aim will be to eradicate perinatal and later life HIV infections completely, it is important to further understand how to support affected families adequately.

For peer review only - http://bmjopen.bmj.com/site/about/guidelines.xhtml

Contributorship Statement: LS, ET, & LC conceptualised the overall study. ET, LC, CW, BS, NL, JJ, JC, MM and WS were involved in study design, data collection and/or data management. KH led and KR, JT, WS, and SZ contributed to the current analyses. LS and KH were predominantly responsible for the writing of the paper, with contributions from the rest of the team. Two adolescent advisory groups served as advisors for contents and acceptability of the questionnaires and recruitment strategies. Community leaders served as advisors for recruitment.

Funding: This study received funding from the European Research Council (ERC) under the European Union's Horizon 2020 research and innovation programme (grant agreement No 771468); the United States Agency for International Development (USAID) Cooperative Agreement No. AID-OAA-LA-13-00001 and was made possible by the generous support of the American people through USAID and the United States President's Emergency Plan for AIDS Relief. The contents are the responsibility of Oxford University and do not necessarily reflect the views of USAID or the United States Government; Research England [0005218]; jointly from the UK Medical Research Council (MRC) and the UK Department for International Development (DFID) under the MRC/DFID Concordat agreement, and by the Department of Health Social Care (DHSC) through its National Institutes of Health Research (NIHR) [MR/R022372/1]; the Leverhulme Trust [PLP-2014-095]; UCL's HelpAge funding; UNICEF Eastern and Southern Africa Office (UNICEF-ESARO); from a CIPHER grant from International AIDS Society [2018/625-TOS]. The views expressed in written materials or publications [OR presented in this presentation] do not necessarily reflect the official policies of the International AIDS society; UKRI GCRF Accelerating Achievement for Africa's Adolescents (Accelerate) Hub (Grant Ref: ES/S008101/1); the Fogarty International Center, National Institute on Mental Health, National Institutes of Health under Award Number K43TW011434. The content is solely the responsibility of the authors and does not represent the official views of the National Institutes of Health; Oak Foundation [grant number: OFIL-20-057]; the Economic and Social Research Council [grant number ES/R501037/1]; the Economic and Social Research Council (grant number ES/J500112/1) the Grand Union - Doctoral Training Partnership; an Economic Social Research Council (ESRC) PhD studentship through the UBEL DTP.

Conflicts of Interest: None to declare

Ethical Approval Statement: Ethical approvals were obtained from the Universities of Oxford (R48876/RE002) and Cape Town (HREC 226/2017) as well as University College

For peer review only - http://bmjopen.bmj.com/site/about/guidelines.xhtml

London for secondary data analysis (14795/001). Additional local approvals and permissions were obtained from the Provincial Departments (Eastern Cape, South Africa) of Health, Education, and Social Development. The current research conforms to the principles embodied in the Declaration of Helsinki.

Data Sharing Agreement: The dataset is available upon request.

Acknowledgements: The authors are grateful to the young mothers and their families, the tireless data collection team, and partner organizations who supported the research process. We also thank our funders, including FHI360, the European Research Council, United States Agency for International Development, USAID, United States President's Emergency Plan for AIDS Relief, Research England, UK Medical Council, UK Department for International Development, National Institute of Health Research, Leverhulme Trust, University College London HelpAge, UNICED Eastern and Southern Africa Office, International AIDS Society, International AIDS society, UKRI GCRF Accelerating Achievement for Africa's Adolescents Hub, Fogarty International Center, National Institute on Mental Health, National Institutes of Health, Oak Foundation and the Economic and Social Research Council.

For peer review only

For peer review only - http://bmjopen.bmj.com/site/about/guidelines.xhtml

REFERENCES

[1] UNAIDS (2020). Infographic- people living with HIV. https://www.unaids.org/sites/default/files/media_asset/2020_aids-data-book_en.pdf [Date Accessed: 14 May 2021]

[2] LeCroix RH, Chan WY, Henrich C et al. Maternal HIV and adolescent Functioning in South Africa: The role of the mother-child relationship. *The Journal of Early Adolescence*. 2020;40(1):83103.doi:10.1177/0272431618824726 [published Online First: 24 January 2019].

[3] Richter LM, Sherr L, Adato M, et al. Strengthening families to support children affected by HIV and AIDS. *AIDS care*. 2009;21(sup1):3-12.doi: 10.1080/09540120902923121 [published Online First: 30 September 2009]

[4] Lowenthal ED, Bakeera-Kitaka S, Marukutira et al. Perinatally acquired HIV infection in adolescents from sub-Saharan Africa: a review of emerging challenges. *The Lancet infectious diseases*. 2014;14(7):627-39.doi:10.1016/S1473-3099(13)70363-3 [published Online First: 7 January 2014].

[5] South African National Department of Health. National Consolidated Guidelines for the Prevention of Mother-To-Child Transmission of HIV (PMTCT) and the Management of HIV in Children, Adolescents and Adults. https://sahivsoc.org/Files/ART%20Guidelines%2015052015.pdf [Date Accessed: 10 September 2021].

[6] Mann JM. A worldwide pandemic. In Gottlieb MS et al., eds. Current Topics in AIDS - Volume 2. Wiley and Sons.

[7] Banks LM, Zuurmond M, Ferrand R, Kuper H. The relationship between HIV and prevalence of disabilities in sub-Saharan Africa: systematic review (FA). *Tropical medicine & international health*. 2015; 20(4):411-29.doi: 10.1111/tmi.12449 [published Online First: 12 December 2014].

[8] McHenry MS, McAteer CI, Oyungu E et al. Neurodevelopment in young children born to HIV-infected mothers: a meta-analysis. *Pediatrics*. 2018;141(2). doi:10.1542/peds.2017-2888 [published Online First: 1 February 2018].

[9] Sherr L, Croome N, Castaneda KP et al. Developmental challenges in HIV infected children—An updated systematic review. *Children and Youth Services Review*. 2014;45:74-89.doi: 10.1016/j.childyouth.2014.03.040 [published Online First: 4 April 2014].

[10] Sherr L, Croome N, Bradshaw K, Parra Castaneda K. A systematic review examining whether interventions are effective in reducing cognitive delay in children infected and affected with HIV. *Aids Care*. 2014;26(sup1):S70-7.doi: 10.1080/09540121.2014.906560 [first Published Online: 10 April 2014].

[11] Townsend C, Schulte J, Thorne C et al. Antiretroviral therapy and preterm delivery-a pooled analysis of data from the United States and Europe. *BJOG : an international journal of obstetrics and gynaecology*.2010; 117(11):1399–1410. doi:10.1111/j.1471-0528.2010.02689.x

[12] Cruz ML, Cardoso CA, Joao EC et al. Pregnancy in HIV vertically infected adolescents and young women: a new generation of HIV-exposed infants. Aids. 2010;24(17):2727-31. doi: 10.1097/QAD.0b013e32833e50d4 [published Online First: 13 November 2010].

[13] Allen AB, Finestone M, Eloff I et al. The role of parenting in affecting the behavior and adaptive functioning of young children of HIV-infected mothers in South Africa. *AIDS and Behavior.* 2014;18(3):605-16.doi:10.1007/s10461-013-0544-7 [published Online First: 28 July 2013].

For peer review only - http://bmjopen.bmj.com/site/about/guidelines.xhtml

[14] Bailey H, Cruz ML, Songtaweesin WN, Puthanakit T. Adolescents with HIV and transition to adult care in the Caribbean, Central America and South America, Eastern Europe and Asia and Pacific regions. *Journal of the International AIDS Society.* 2017;20:21475.doi:10.7448/IAS.20.4.21475 [published Online First: 16 May 2017].
[15] Earnshaw VA, Kidman RC, Violari A. Stigma, depression, and substance use problems among perinatally HIV-infected youth in South Africa. *AIDS and Behavior*. 2018; 22(12):3892-6.doi: 0.1007/s10461-018-2201-7 [published Online First: 16 June 2018].
[16] Rochat T, Netsi E, Redinger S, Stein A. Parenting and HIV. *Current opinion in psychology*. 2017;15:155-61.doi: 10.1016/j.copsyc.2017.02.019 [published Online First: 22 February 2017].
[17] Sherr L, Cluver LD, Toska E, He E. Differing psychological vulnerabilities among behaviourally and perinatally HIV infected adolescents in South Africa–implications for targeted health service provision. *AIDS care*. 2018;30(sup2):92-101. 10.1080/09540121.2018.1476664 [published Online First: 30 May 2018].
[18] Anderson M, Elam G, Gerver S, et al. “It took a piece of me”: Initial responses to a positive HIV diagnosis by Caribbean people in the UK. *AIDS care*. 2010;22(12):1493-8. doi: 10.1080/09540121.2010.482125 [published Online First: 6 December 2018].
[19] Ramirez-Avila L, Regan S, Giddy J, et al. Depressive symptoms and their impact on health-seeking behaviors in newly-diagnosed HIV-infected patients in Durban, South Africa. *AIDS and Behavior*. 2012;16(8):2226-35.doi: 10.1007/s10461-012-0160-y [published Online First: 27 March 2012].
[20] Yah CS, Ndlovu S, Kutywayo A, et al. The prevalence of pregnancy among adolescent girls and young women across the Southern African development community economic hub: A systematic review and meta-analysis. *Health promotion perspectives*. 2020;10(4):325. doi:10.34172/hpp.2020.51 [published Online First: 7 November 2020].
[21] Glassman AL, Silverman R, McQueston K. Adolescent fertility in low-and middle-income countries: effects and solutions. Center for Global Development Working Paper. 2012;295.Available at SSRN: https://ssrn.com/abstract=2102763 or http://dx.doi.org/10.2139/ssrn.2102763 [Date Accessed: 10 September 2021]
[22] Toska E, Laurenzi CA, Roberts KJ et al. Adolescent mothers affected by HIV and their children: a scoping review of evidence and experiences from sub-Saharan Africa. *Global Public Health.* 2020;15(11):1655-73.doi: 10.1080/17441692.2020.1775867 [published Online First: 6 June 2020].
[23] Mullen EM. Mullen scales of early learning. Circle Pines, MN: AGS 1995:58-64.
[24] Bornman J, Romski M, Tonsing K, et al. Adapting and translating the Mullen Scales of Early Learning for the South African context. *South African Journal of Communication Disorders*. 2018;65(1):1-9.doi: 10.4102/sajcd.v65i1.571[published Online First: 8 March 2018].
[25] Koura GK, Boivin MJ, Davidson LL et al. Adaptation of the mullen scales of early learning to Benin setting. *American Journal of Epidemiology*. 2012;175(11):S128-.
[26] Milosavljevic B, Vellekoop P, Maris H, et al. Adaptation of the Mullen Scales of Early Learning for use among infants aged 5-to 24-months in rural Gambia. Developmental science. 2019;22(5):e12808. doi:10.1111/desc.12808 [published Online First: 9 February 2019].
[27] Durkin MS, Wang W, Shrout PE et al. Evaluating a Ten Questions screen for childhood disability: reliability and internal structure in different cultures. *Journal of Clinical Epidemiology*, 1995;48(5):657-66. doi:10.1016/0895-4356(94)00163-k

For peer review only - http://bmjopen.bmj.com/site/about/guidelines.xhtml

[28] Pillay U, Roberts B, Rule S. South African social attitudes: changing times, diverse voices. Cape Town: HSRC press 2006.
[29] Statistics South Africa. *Census 2001: Household Questionnaire*. Pretoria, SA: Statistics SA 2001.
[30] Labadarios D, Steyn NP, Maunder E et al. The national food consumption survey (NFCS): South Africa, 1999. *Public health nutrition*. 2005;8(5):533-43.doi:10.1079/phn2005816 [published Online First: 2 January 2007].
[31] Duong M, Piroth L, Grappin M et al. Evaluation of the Patient Medication Adherence Questionnaire as a tool for self-reported adherence assessment in HIV-infected patients on antiretroviral regimens. *HIV clinical trials*. 2001;2(2):128-35.doi: 10.1310/M3JR-G390-LXCM-F62G [published Online First: 29 January 2015].
[32] Pantelic M, Boyes M, Cluver L, Thabeng M. 'They say HIV is a punishment from god or from ancestors': cross-cultural adaptation and psychometric assessment of an HIV stigma scale for South African adolescents living with HIV (ALHIV-SS). *Child indicators research*. 2018;11(1):207-23. doi:10.1007/s12187-016-9428-5 [published Online First: 23 November 2016].
[33] Kovacs M. Children's depression inventory (CDI). Toronto: Multi-Health System 2003.
[34] Reynolds CR, & Richmond BO. Revised Children's Manifest Anxiety Scales (RCMAS). Los Angeles: Western Psychological Services 1985.
[35] Amaya-Jackson L, McCarthy G, Cherney MS & Newman E. Child PTSD checklist. Durham, NC: Duke University Medical Center 1995.
[36] Sheehan DV, Lecrubier Y, Sheehan KH et al. The Mini-International Neuropsychiatric Interview (MINI): the development and validation of a structured diagnostic psychiatric interview for DSM-IV and ICD-10. *Journal of clinical psychiatry*. 1998;59(20):22-33.
[37] Martin L, Revington N, Seedat S. The 39-item Child Exposure to Community Violence (CECV) Scale: Exploratory factor analysis and relationship to PTSD symptomatology in trauma-exposed children and adolescents. *International journal of behavioral medicine*. 2013;20(4):599-608.doi: 10.1007/s12529-012-9269-7 [published Online First: 2 October 2012].
[38] Finkelhor D, Hamby SL, Ormrod R, Turner H. The Juvenile Victimization Questionnaire: reliability, validity, and national norms. *Child abuse & neglect*. 2005;29(4):383-412.doi: 10.1016/j.chiabu.2004.11.001 [published Online First: 28 April 2005].
[39] Berry JO, Jones WH. The parental stress scale: Initial psychometric evidence. *Journal of social and personal relationships*. 1995 Aug;12(3):463-72.doi: 10.1177/0265407595123009
[40] Sherbourne CD, Stewart AL. The MOS social support survey. *Social science & medicine*. 1991;32(6):705-14.doi: 10.1016/0277-9536(91)90150-B
[41] Casale M. The importance of family and community support for the health of HIV-affected populations in Southern Africa: What do we know and where to from here?. *British Journal of Health Psychology*. 2015;20(1):21-35.doi: 10.1111/bjhp.12127 [first Published Online: 9 December 2014].
[42] Thomas T, Ahmed Y, Tan M, Grigorenko EL. Cognitive and Educational Interventions for Orphans and Vulnerable Children Affected by HIV/AIDS: A Meta-Analysis. *Child development*. 2021;92(1):7-20. DOI: 10.1111/cdev.13438
[43] Mebrahtu H, Simms V, Mupambireyi Z et al. Effects of parenting classes and economic strengthening for caregivers on the cognition of HIV-exposed infants: a pragmatic cluster randomised controlled trial in rural Zimbabwe. *BMJ global health*.

For peer review only

For peer review only - http://bmjopen.bmj.com/site/about/guidelines.xhtml

2019;4(5):e001651.doi: 10.1136/bmjgh-2019-001651[published Online First: 11 September 2019].

[44] Rao N, Sun J, Wong JM et al. Early childhood development and cognitive development in developing countries: A rigorous literature review. http://cerc.edu.hku.hk/wp-content/uploads/ECD-review.pdf [Date accessed: 10 May 2021]

[45] Johnson LF, Dorrington RE, Bradshaw D et al. The effect of educational attainment and other factors on HIV risk in South African women: results from antenatal surveillance, 2000–2005. *Aids*. 2009;23(12):1583-8. doi: 10.1097/QAD.0b013e32832d407e [published Online First: 31 July 2009].

[46] Merville O, Puangmala P, Suksawas P et al. School trajectory disruption among adolescents living with perinatal HIV receiving antiretroviral treatments: a case-control study in Thailand. *BMC public health*. 2021;21(1):1-2.doi: 10.1186/s12889-021-10189-x [published Online First: 21 January 2021].

[47] Toska E, Cluver L, Orkin M, Bains A, Sherr L, Berezin M, Gulaid L. Screening and supporting through schools: Educational experiences and needs of adolescents living with HIV in a South African cohort. *BMC Public Health*. 2019;19(1):1-0. doi: 10.1186/s12889-019-6580-0 [published Online First: 6 March 2019].

[48] Toska E, Gittings L, Hodes R, et al. Resourcing resilience: social protection for HIV prevention amongst children and adolescents in Eastern and Southern Africa [published correction appears in Afr J AIDS Res. 2016 Sep;15(3):314]. *Afr J AIDS Res*. 2016;15(2):123-140. doi:10.2989/16085906.2016.1194299

[49] van der Wal R, Loutfi D, Hong QN, Vedel I, Cockcroft A, Johri M, Andersson N. HIV-sensitive social protection for vulnerable young women in East and Southern Africa: a systematic review. J Int AIDS Soc. 2021 Sep;24(9):e25787. doi: 10.1002/jia2.25787. PMID: 34473406; PMCID: PMC8412122.

[50] Pantelic M, Steinert JI, Park J, Mellors S, Murau F. 'Management of a spoiled identity': systematic review of interventions to address self-stigma among people living with and affected by HIV. BMJ Glob Health. 2019*; 4*(2):e001285. doi:10.1136/bmjgh-2018-00128

For peer review only - http://bmjopen.bmj.com/site/about/guidelines.xhtml

Figure Legend

Figure 1

Flow Chart of Classification by Mode of Maternal HIV Acquisition

For peer review only

For peer review only - http://bmjopen.bmj.com/site/about/guidelines.xhtml

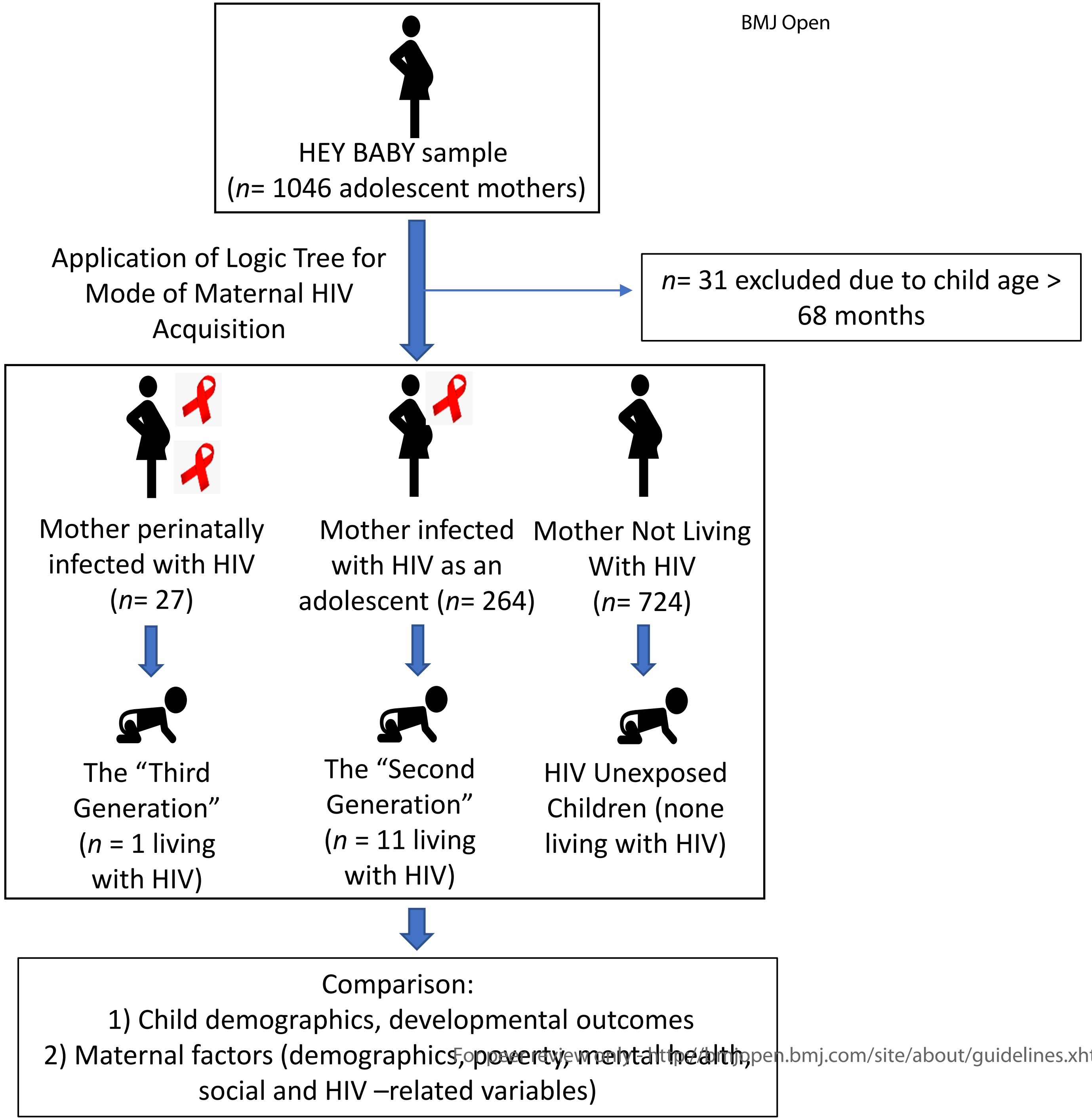

For peer review only - http://bmjopen.bmj.com/site/about/guidelines.xhtml

Appendix 1

Infant (0-68 month) developmental outcomes, according to familial patterns of HIV infection – sample limited to adolescent mothers only

	Overall (*N*=1015)	Third Generation (*n*= 23)	Second Generation (*n*= 229)	No HIV Exposure (*n*= 721)	p-value
**Mullen Scales of Child Development** *(M, SD)*					
Gross Motor Score*	49.7 (12.5)	54.3 (16. 7)[a]	46.8 (12.7)[b]	50.3 (12.2)	<.001
Visual Reception Score	42.1 (14.2)	44.0 (13.7)	40.4 (14.1)	42.5 (14.2)	.12
Fine Motor Score	43.7(14.7)	45.0 (16.2)	41.3 (15.5)[b]	44.4 (14.3)	.017
Receptive Language Score	47.5 (13.5)	48.5 (14.0)	46.4 (14.9)	47.8 (13.0)	.37
Expressive Language Score	51.6 (13.5)	51.1 (14.0)	51.0 (14.8)	51.8 (13.0)	.71
Composite Score	93.3 (21.3)	95.0 (23.1)	90.6 (22.8)	94.1 (20.7)	.098
**WHO Disability Score- Any Disability** *(N, %)*	234 (26.9%)	6 (27.3%)	63 (28.9%)	165 (26.2%)	.73
**Any Days Sick** (past month) *(N, %)*	212 (22.1%)	3 (15.0%)	37 (16.2%)	172 (24.2%)	.032

Notes. Group comparisons were conducted using χ^2 tests for categorical variables and univariate analyses of variance for continuous variables. *Gross motor score: n = 831. Mullen subdomain scores range from 20-80, composite scores from 49-155. Tukey's range post hoc tests were undertaken to identify mean differences between groups for continuous variable: [a] Statistically different from the second generation (p<0.05), [b] Statistically different from HIV unexposed group (p<0.05).

For peer review only

BMJ Open

The development of young children born to young mothers with no, first or second generation HIV acquisition in the Eastern Cape province, South Africa: a cross-sectional study

Journal:	*BMJ Open*
Manuscript ID	bmjopen-2021-058340.R3
Article Type:	Original research
Date Submitted by the Author:	30-Sep-2022
Complete List of Authors:	Sherr, Lorraine; University College London<br>Haag, Katharina; UCL, Institute for Global Health<br>Roberts, Kathryn; UCL, Institute for Global Health<br>Cluver, Lucie; University of Oxford, Centre for Evidence-Based Social Intervention; University of Cape Town, Department of Psychiatry and Mental Health<br>Wittesaele, Camille; University of Oxford, Department of Social Policy & Intervention; London School of Hygiene & Tropical Medicine, Department of Infectious Disease Epidemiology<br>Saliwe, Bongiwe; University of Cape Town, Centre for Social Science Research<br>Tolmay, Janke; University of Cape Town, Centre for Social Science Research<br>Langwenya, Nontokozo; University of Cape Town, Centre for Social Science<br>Jochim, Janina; University of Oxford, Department of Social Policy & Intervention<br>Saal, Wylene; University of Cape Town, Centre for Social Science Research<br>Zhou, Siyanai; University of Cape Town, Centre for Social Science Research<br>Marlow, Marguerite; University of Stellenbosch, Institute for Life Course Health Research, Department of Global Health, Faculty of Medicine and Health Sciences<br>Chen-Charles, Jenny; University of Oxford, Department of Social Policy & Intervention<br>Toska, Elona; University of Cape Town Faculty of Humanities, Centre for Social Science Research & Department of Sociology; University of Oxford, Social Policy and Intervention
**Primary Subject Heading**:	HIV/AIDS
Secondary Subject Heading:	Global health, Mental health
Keywords:	HIV & AIDS < INFECTIOUS DISEASES, Community child health < PAEDIATRICS, EPIDEMIOLOGY, Health policy < HEALTH SERVICES ADMINISTRATION & MANAGEMENT, Developmental neurology & neurodisability < PAEDIATRICS

SCHOLARONE™
Manuscripts

I, the Submitting Author has the right to grant and does grant on behalf of all authors of the Work (as defined in the below author licence), an exclusive licence and/or a non-exclusive licence for contributions from authors who are: i) UK Crown employees; ii) where BMJ has agreed a CC-BY licence shall apply, and/or iii) in accordance with the terms applicable for US Federal Government officers or employees acting as part of their official duties; on a worldwide, perpetual, irrevocable, royalty-free basis to BMJ Publishing Group Ltd ("BMJ") its licensees and where the relevant Journal is co-owned by BMJ to the co-owners of the Journal, to publish the Work in this journal and any other BMJ products and to exploit all rights, as set out in our licence.

The Submitting Author accepts and understands that any supply made under these terms is made by BMJ to the Submitting Author unless you are acting as an employee on behalf of your employer or a postgraduate student of an affiliated institution which is paying any applicable article publishing charge ("APC") for Open Access articles. Where the Submitting Author wishes to make the Work available on an Open Access basis (and intends to pay the relevant APC), the terms of reuse of such Open Access shall be governed by a Creative Commons licence – details of these licences and which Creative Commons licence will apply to this Work are set out in our licence referred to above.

Other than as permitted in any relevant BMJ Author's Self Archiving Policies, I confirm this Work has not been accepted for publication elsewhere, is not being considered for publication elsewhere and does not duplicate material already published. I confirm all authors consent to publication of this Work and authorise the granting of this licence.

For peer review only

Title: **The development of young children born to young mothers with no, first or second generation HIV acquisition in the Eastern Cape province, South Africa: a cross-sectional study**

Authors: Lorraine Sherr[1], Katharina Haag[1], Kathryn J. Steventon Roberts[1,2], Lucie Dale Cluver[2,3], Camille Wittesaele2,[4], Bongiwe Saliwe[5], Janke Tolmay[5], Nontokozo Langwenya[5], Janina Jochim[2], Wylene Saal[5], Siyanai Zhou[5], Marguerite Marlow[6], Jenny Chen-Charles[2], Elona Toska[2,5,7]

1 Institute for Global Health, University College London, UK

2 Department of Social Policy & Intervention, University of Oxford, Oxford, UK

3 Department of Psychiatry and Mental Health, University of Cape Town, Cape Town, South Africa

4 Department of Infectious Disease Epidemiology, London School of Hygiene & Tropical Medicine, London, UK

5 Centre for Social Science Research, University of Cape Town, Cape Town, South Africa

6 Institute for Life Course Health Research, Department of Global Health, Faculty of Medicine and Health Sciences, Stellenbosch University, South Africa

7 Department of Sociology, University of Cape Town, Cape Town, South Africa

Corresponding author:

Professor Lorraine Sherr (l.sherr@ucl.ac.uk), University College London, Department of Global Health, Royal Free Hospital Campus, Rowland Hill St, London, NW3 2PF

Tel: +442080168071, ext 68071

Fax: +44 207 794 1224

Word Count: 4668 (excluding title page, abstract, references, figures, and tables)

For peer review only

For peer review only - http://bmjopen.bmj.com/site/about/guidelines.xhtml

ABSTRACT

Background: The inter-generational effects of HIV require long term investigation. We compared developmental outcomes of different generations impacted by HIV – children of mothers not living with HIV, the "second generation" (i.e., with recently infected mothers), and the "third generation" (i.e., children of perinatally infected mothers).

Methods: A cross-sectional community sample of *N*=1015 young mothers (12-25 years) and their first children (2- 68 months, 48.2% female), from South Africa's Eastern Cape Province. 71.3% (*n*=724) of children were born to mothers not living with HIV; 2.7% (*n*=27; 1 living with HIV) were third and 26.0% (*n*=264; 11 living with HIV) second generation children. Child scores on the Mullen Scales of Early Learning (MSEL), the WHO Ten Questions Screen for Disability and maternal demographics were compared between groups using χ2-tests and univariate approach, analysis of variance (ANOVA) analysis. Hierarchical linear regressions investigated predictive effects of familial HIV infection patterns on child MSEL composite scores, controlling for demographic and family environment variables.

Results: Second generation children performed poorer on gross (*M*=47.0, *SD*=13.1) and fine motor functioning (*M*=41.4, *SD*=15.2) and the MSEL composite score (*M*=90.6, *SD*=23.0) than children with non-infected mothers (gross motor: *M*=50.4, *SD*=12.3; fine motor: *M*=44.4, *SD*=14.1; composite score: *M*=94.1, *SD*=20.7). The third generation performed at similar levels to non-exposed children (gross motor: *M*=52.4, *SD*=16.1; fine motor: *M*=44.3, *SD*=16.1, composite score: *M*=94.7, *SD*=22.2), though analyses were underpowered for definite conclusions. Hierarchical regression analyses suggest marginal predictive effects of being second generation child compared to having a mother not living with HIV (*B*=-3.3, *CI*=-6.8; .1) on MSEL total scores, and non-significant predictive effects of being a third generation child (*B*= 1.1, *CI* = -7.5: 9.7) when controlling for covariates. No group differences were found

For peer review only - http://bmjopen.bmj.com/site/about/guidelines.xhtml

for disability rates (26.9% third generation, 27.7% second generation, 26.2% non-exposed; $\chi^2 = .02$, $p = .90$).

Conclusion: Recently infected mothers and their children may struggle due to the disruptiveness of new HIV diagnoses and incomplete access to care/ support during pregnancy and early motherhood. Longstanding familial HIV infection may facilitate care pathways and coping, explaining similar cognitive development among not exposed and third generation children. Targeted intervention and fast-tracking into services may improve maternal mental health and socioeconomic support.

Keywords: HIV; adolescent mothers, infants, cognitive development, mental health, environmental factors

For peer review only

STRENGTHS AND LIMITATIONS OF THIS STUDY

- To our knowledge, this is the first study to investigate developmental outcomes and life circumstances of the "third generation", i.e. children with a perinatally infected mothers.
- A particular strength is the comparison to both children of the "second generation" (i.e., with recently infected mothers) and children not affected by HIV but living in similar high-risk environments characterized by poverty and deprivation.
- While we found that children of the third generation perform equally well or better than children not directly exposed to HIV, the sample of the former was small, limiting our power to draw definite conclusion.
- Our sample consisted predominantly of young mothers. While both rates of HIV infection and young pregnancy may be compounded in deprived areas, the current findings need to be replicated in a more representative samples.

For peer review only

INTRODUCTION

Approximately 7.5 million people in South Africa were living with HIV in 2020, including 360,000 adolescents aged 10-19 years.[1] Generally, HIV infections have been linked to higher rates of morbidity, stigma, poverty, and other adverse outcomes.[2, 3] While rates of perinatal HIV infection are declining, due to improved screening and treatment options [4-6], historical exposure means that an increasing number of perinatally-infected children are now reaching adolescence and young adulthood and are becoming parents themselves. There is a need to study their children's developmental outcomes, in order to devise effective support for these families.

To knowledge of the authors, to date, there have been limited studies focusing on this "third generation", i.e., children who have their mother perinatally infected by HIV. Most designs do not differentiate between mothers who were recently or perinatally infected and instead focus on comparing HIV infected, HIV exposed and uninfected, and HIV unexposed children.[7-9] Findings suggest that the former two groups may show developmental sequelae (including cognitive and motor), which can be explained by exposure to the virus (in utero) and Antiretroviral therapies (ART) as well as environmental factors associated with HIV infection (e.g., prematurity, poor parental mental health and substance use, stigma, caretaker changes, food insecurity).[10, 11] However, specific knowledge on the third generation is still extremely limited. The few existing studies focus mainly on maternal characteristics (e.g., ART adherence, age at pregnancy) and child infection status.[12]

Of note, previous research on adolescents who were perinatally infected (potential mothers of the third generation) versus recently infected (potential mothers of the second generation) indicates that they may be facing different types of life challenges that could affect their parenting in divergent ways. Those perinatally infected tend to have experienced more chronic adversity, such as parental and/or sibling illness and death, caretaking responsibilities,

For peer review only - http://bmjopen.bmj.com/site/about/guidelines.xhtml

poorer parenting and compromised parent-child relationships, poor wider family and community support, and compounded internalized and associative stigma, all of which may increase the risk for lower well-being and higher substance use.[2, 4, 13-17] Those recently infected have more likely been marginalised and struggle with the impact of their diagnosis, including biographical disruptions and reduced hope for the future, and to have difficulties with accessing and engaging in care. All of these issues may be compounded by mental health problems including anxiety, depression, substance use, and internalized stigma.[14, 17-19]

The current study was set up to explore the development of young children (2-68 months) of adolescent and young adult mothers living in the Eastern Cape of South Africa, with a specific aim to gain a better understanding of third- and second-generation HIV effects. HIV infections often tend to cluster in disadvantaged environments, leading to disproportionally affected communities, where risks of initial HIV infection and HIV being passed on across generations are heightened. These communities are often also affected by high adolescent pregnancy rates,[20] which are associated with a range of risks for mother and child such as poor educational prospects, exposure to poverty and violence, and poor developmental outcomes.[21,22] Furthermore, they can also be a marker for adolescent sexual risk behaviours, and there may be a syndemic occurrence between HIV and adolescent parenthood risk among young women in sub-Saharan Africa.[22]

We had two main analysis aims: 1) to compare child developmental scores (fine and gross motor, visual reception, expressive and receptive language skills) at ages 2-68 months, independent of child HIV status, in children of mothers 19 years or younger who were not living with HIV, were recently infected through sexual exposure (second generation) and were perinatally infected (third generation), and 2) to investigate how mothers of these groups may differ in terms of their socio-economic status, well-being, and social support.

METHODS

For peer review only - http://bmjopen.bmj.com/site/about/guidelines.xhtml

Participants

Data utilised within these analyses originate from the "Helping Empower Youth Brought up in Adversity with their Babies and Young children" (HEY BABY) study. The study was conducted in rural and peri-urban health districts of South Africa's Eastern Cape and aimed at investigating the effects of adolescent motherhood, as well as intergenerational effects of HIV exposure. 1046 adolescent and young adult mothers (10-25 years) with at least one living child were interviewed between March 2018 and July 2019. The required sample size was estimated based on expected effect sizes for key outcomes. Participants were partially recruited from a previous study (*n*= 159: any young mothers included), as well as through six parallel sampling strategies developed in cooperation with local experts and an adolescent mother advisory group to ensure representativeness (*n*= 887: only adolescent mothers). This comprised sampling through 73 known health facilities within the districts, 43 secondary schools, 9 maternity units, and referrals by service providers, social workers, and adolescent mothers themselves. 95%-98% of eligible mothers from each recruitment channel were successfully enrolled into the study. The current analyses were limited to data on first child of the young mother only (10-25 years), and children aged 68 months or younger who were within the normed range for the Mullen Scales (*n*= 31 excluded based on this age restriction), limiting the sample to n = 1015. We also performed analyses including adolescent mothers (age at pregnancy ≤ 19, *n*= 972) only for the key outcomes (see Appendix 1), as these mothers had been the main recruitment focus for the study.

Patient and Public Involvement

The study team were advised on recruitment methods by adolescent mothers, whose suggestions were included in the study protocol. Furthermore, the team has been working with two Teen Advisory groups in the Eastern and Western Cape of South Africa, who were

For peer review only - http://bmjopen.bmj.com/site/about/guidelines.xhtml

involved in piloting the study. Feedback was incorporated to improve relevance and acceptability of the questionnaire and the research procedure. Finally, through the engagement of community leaders, it was ensured that recruitment strategies were effective, sensitive to participant's circumstances and minimised stigma risks.

Procedure

The dataset analysed combined four data sources. Firstly, all adolescent mother participants completed a detailed study questionnaire relating to socio-demographic characteristics, sexual and reproductive health, physical and mental health, relationships, and social support. Secondly, they completed an adolescent parent questionnaire, which collected data on maternal and child health, child development, the father of the child, and maternal factors including social support, the parenting experience, and violence exposure. Thirdly, cognitive assessments of the children were performed by a trained administrator, using the Mullen Scales of Early Learning. Finally, details from the child's medical records (*Road to Health Booklet*) were also included in the database. All participants provided written consent, and interviews were conducted in the language of their choice (i.e., English or isiXhosa). Regardless of participation, all adolescents received a small 'snack pack' containing a snack and juice during the interview, and a small 'thank you pack', with personal products such as toothpaste and a toothbrush. Items included in these packs were selected by the adolescent advisory group as preferable and appropriate. New mothers also received a 'baby pack', the contents of which were also chosen by an adolescent advisory group, and included nappies and baby cream.

For peer review only - http://bmjopen.bmj.com/site/about/guidelines.xhtml

Measures

Mode of maternal HIV acquisition

Mode of maternal HIV acquisition (perinatal versus recent) was assessed through an algorithm, given that the study was community-based and not linked to clinical testing data. Accordingly, it was derived using a logic tree based on clinical and fieldwork experiences .[17] The algorithm allowed for categorisation according to self-report, age of ART initiation, and parental death information (Tolmay, Saal et al., in preparation). For the current analyses, we compared the following three groups: children of the third generation (mother perinatally infected), children of the second generation (mother recently infected), and children of mothers not living with HIV (see Figure 1). Child HIV status was not taken into account in these classifications, since absolute numbers were low (n = 12 based on maternal self-reported data), with only one of these children being in the third generation. Thus, we were underpowered to study these children separately. We decided however to retain them in the analyses since they are relevant members of the second and third generations respectively.

Child developmental outcomes

Child developmental outcomes were assessed using the Mullen Scales of Early Development (MSEL).[23] The MSEL is normed for children aged 0 to 68 months (USA) and assesses child performance across five domains: gross motor (only for age <39 months), fine motor, visual reception, receptive and expressive language (score range: 20-80). A composite score (score range: 49-155) can be derived, and the scales have been validated for use in Sub-Saharan Africa.[24-26] Child disability status was assessed using the WHO Ten Questions Screen for Disability.[27] This measure can be applied to detect common disabilities (hearing, visual, physical, speech, mental, and epilepsy) in children. A score indicating any disability ("yes" across any of the 10 items) was derived for the current analyses.

For peer review only - http://bmjopen.bmj.com/site/about/guidelines.xhtml

Maternal variables

Adolescent mothers were compared on various variables, including demographic factors (maternal age, maternal and paternal age at pregnancy), child feeding method used (formula, breastfeeding, combined, other), maternal school progression (self-report: repeated at least one school grade), poverty (number of the eight socially perceived necessities for children the family had access to, [28] household access to any government cash transfers (child support, foster child, pension, disability, or care dependency grant, measured via South Africa Census item [29]), food security (number of days there was not enough food for the household in the past seven days [30]), full ART adherence over the past seven days (Patient Medication Adherence Questionnaire [31]), any HIV clinic appointments missed in the past year, extent of HIV-related stigma (Adolescents Living with HIV Stigma Scale [32]), depressive symptoms in the past two weeks (Child Depression Inventory-Short Form [33])), anxiety symptoms in the past month (Revised Children's Manifest Anxiety Scale [34]), PTSD symptoms in the past month (Child PTSD Checklist-Short Form [35]), suicidality symptoms during the past month (Mini International Psychiatric Interview for Children and Adolescents-Suicidality and Self-Harm Subscale [36]), extent of community violence exposure in the past year (item from the Child Exposure to Community Violence Checklist [37]), intimate partner violence exposure in the past month (Juvenile Victimization Questionnaire [38]), current parenting stress (Parental Stress Scale [39]) and amount of social support received (adjusted version of the Medical Outcomes Study Social Support Survey [40], previously used in the South African context [41]).

Statistical analyses

All analyses were conducted using STATA 16 SE. Firstly, child and adolescent mother descriptive information for the three groups of interest (children of the third generation, children of the second generation, and HIV-unexposed children) was provided. Next, child

For peer review only - http://bmjopen.bmj.com/site/about/guidelines.xhtml

developmental outcomes and maternal factors surrounding socio-economic status, HIV-related variables (only HIV-affected mothers), mental health and social environment were compared between groups, using χ2 tests and univariate analyses of variance (ANOVA) as appropriate. For continuous variables, Tukey's range tests were used to conduct post-hoc group comparisons. Effects at p<.05 were considered relevant. Finally, a hierarchical regression model was run, with the child composite score on the Mullen scales as a key outcome. In a first step, child familial HIV exposure (third generation, second generation, HIV-unexposed) was added as a predictor. In a second step, we included relevant control variables that were found to be associated with the predictor or outcome variables in univariate analyses or were identified as relevant within the literature to see whether child familial HIV exposure would remain a relevant predictor.

RESULTS

Descriptive variables

After applying the logic tree for mode of maternal HIV acquisition, 27 mothers (2.7%) were classified as perinatally infected, 264 (26.0%) as recently/sexually infected, and 724 (71.3%) as not living with HIV. Table 1 presents adolescent mother and child sample characteristics, split according to whether the child was in the third generation, the second generation, or not exposed to HIV. There were no significant group differences in child sex, HIV status, and creche attendance; however, children of the second generation were on average older than those without HIV exposure (*Diff*= 6.7, *95% CI*= 4.3; 9.1).No significant age differences were found between children of the third generation and the other two groups (*Diff*= 2.6, *95% CI*= -4.2; 9.5). Formula (without breastfeeding) use was the most common feeding method during the first six months of the child's life for mothers of the third generation (63.0%). For mothers of the second generation, formula use (only) and breastfeeding (only)

For peer review only - http://bmjopen.bmj.com/site/about/guidelines.xhtml

were similarly common (35.0% and 38.4%), while the majority of mothers of HIV-unexposed children opted for a mix of both (40.9%).

Similar to their children, mothers of the second generation (*M*= 19.7 years) were slightly older than those of third generation (*M*= 18.7 years), and the latter were older than mothers of HIV-unexposed children (*M*= 17.7 years) (F= 148.01, p<.001). Furthermore, mothers of the second and third generation were older at pregnancy with the participating child (*M*= 17.8 and *M*= 17.3 versus *M*= 16.3 years respectively; F= 89.23, p <.001) compared to mothers of HIV-unexposed children. Fathers of the second generation (*M*= 23.2 years) were on average older at childbirth than those of the third generation and HIV-unexposed children (*M*= 21.3 and *M*= 20.4 years respectively, F= 49.08, p <.001). There was no difference between groups regarding whether the mother lived in informal housing or a rural area, though mothers unaffected by HIV were marginally less likely to be in a relationship (64.3%, versus 77.8%% for the third generation and 71.5% for the second generation; χ^2= 6.52; p= .038). Mothers of the second generation were more likely to have repeated a school grade (69.3%), versus 61.5% for mothers of the third generation and 57.9% for mothers of HIV-unexposed children (χ^2= 9.91, p= .007).

Mothers of the second and third generations had on average fewer of the eight necessities available (F= 10.11, p< .001) (see Table 1); post-hoc tests identified significant differences for second generation (p< .001) and non- significant differences for third generation mothers (p = .060) compared to those not living with HIV. Mothers of the second and third generations were also more likely to receive household cash grants (χ^2= 8.10; p= .017). However, mothers of the third generation had the lowest average days on which they had no food available for the child in the past week, followed by mothers not living with HIV, and then recently infected mothers, though the overall model was only close to significant (F=2.73; p = .066).

For peer review only - http://bmjopen.bmj.com/site/about/guidelines.xhtml

Table 1

Sociodemographic characteristics of adolescent mothers and their children according to familial patterns of HIV infection

	Overall (*N*=1015)	Third Generation (*n*= 27)	Second Generation (*n*= 264)	No HIV Exposure (*n*= 724)	p-value
**Child Variables**					
Age in months (M, SD)	18.7 (14.7)	20.9 (16.7)	23.5 (16.4)[b]	16.8 (13.5)	<.001
Sex (1= female)	489 (48.2%)	16 (59.3%)	123 (48.2%)	350 (48.3%)	.46
HIV Status	12 (5.0%)	1 (9.1%)	11 (10.0%)	-	.92
Feeding					
Breastfeeding And Formula	347 (34.2%)	4 (14.8%)	47 (17.9%)	296 (40.9%)	<.001
Baby Formula Only	318 (31.4%)	17 (63.0%)	92 (35.0%)	209 (28.9%)	
Breastfeeding Only	258 (25.4%)	5 (18.5%)	101 (38.4%)	152 (21.0%)	
Other Milk or Water	91 (9.0%)	1 (3.7%)	23 (8.8%)	67 (9.3%)	
Creche Attendance	304 (29.8%)	8 (29.6%)	80 (30.4%)	216 (29.8%)	.98
**Maternal Variables**					
Age in years (M, SD)	18.2 (1.8)	18.7 (1.6)[a],[b]	19.7 (2.0)[b]	17.7 (1.5)	<.001
Age at Pregnancy With The Participating Child (years; M, SD)	16.8 (1.7)	17.3 (2.0)	17.8 (1.9)	16.3 (1.4)	<.001
Paternal Age at Pregnancy (years, M, SD)	21.1 (3. 9)	21.3 (4.3)[a]	23.2 (4.08)[b]	20.4 (3.5)	<.001
Informal Housing	217 (21.9%)	6 (23.1%)	60 (23.6%)	151 (21.2%)	.76
Living in Rural Area	293 (28.9%)	8 (29.6%)	63(23.9%)	222 (30.7%)	.11
Any School Grade Repeated	581 (60.8%)	16 (61.5%)	169 (69.3%)	396 (57.8%)	.007
Current Relationship	670 (66.5%)	21 (77.8%)	186 (71.8%)	463 (64.2%)	.038
**Poverty and Social Protection**					
Number of Eight Necessities Available	5.2 (2.2)	4.4 (2.7)	4.8 (2.3)[b]	5.4 (2.1)	<.001
Any Household Grant	944 (92.5%)	26 (96.3%)	254 (96.2%)	659 (91.1%)	.017
Days Insufficient Food for Child (past week)	.3 (1.1)	.0 (.2)	.4 (1.2)	.3 (1.0)	.07

Notes. Group comparisons were conducted using χ2 tests for categorical variables and univariate analyses of variance for continuous variables. Tukey 's range post hoc tests were undertaken to identify mean differences between groups for continuous variables: [a] Statistically

For peer review only - http://bmjopen.bmj.com/site/about/guidelines.xhtml

different from the second generation (p<0.05), [b] Statistically different from HIV unexposed group (p<0.05). Underlying Ns vary depending on missing values (N= 933 to N= 1015).

Child developmental outcomes

Table 2 presents child developmental outcomes according to familial patterns of HIV infection. For the Mullen Scales of Early Child Development, we found that the third generation had the highest gross motor scores (M= 52.4, SD= 16.1), ahead of children not exposed to HIV (M= 50.4, SD = 12.3) and the second generation (M= 47.0, SD = 13.1; F = 6.50, p= .001). However, due to low power resulting from the small sample of children in the third generation, Tukey's range post hoc tests only identified a difference between the latter two groups (*Diff*= 3.41, *95% CI*= 1.08; 5.73). Children of the second generation (M= 41.4, SD= 15.2) exhibited poorer fine motor scores than both HIV non-exposed children (M= 44.4, SD= 14.1) and children of the third generation (M= 44.3, SD= 16.1; F= 4.26, p= .014), though post-hoc tests again only indicated differences between the former two groups (*Diff*= 3.04, *95%CI*= .59: 5.49). No differences were found on the visual reception, receptive, and expressive language subscales, though scores for the second generation were consistently lowest, while children of the third generation tended to do as well or even better than non-exposed children. In terms of the composite scores, children of the second generation performed marginally lower (M= 90.6, SD= 23.0) than those not exposed to HIV (M= 94.1, SD= 20.7; p= .055) in post-hoc tests (*Diff*= 3.55, *95%CI*= -.05; 7.16). Differences of the former to third generation (M= 94.7, SD= 22.2) were not significant in post-hoc tests (p= .66), despite the scores of the third generation being approximately equal to those of non-infected children (overall model: F= 2.72 p= .066). Of note, all groups performed lower than expected compared to the US reference population based on which the Mullen Scales were developed (M= 100.0 for the composite score; p<.001). No group differences were found for presence of any disability (χ^2 = .02, p= .90). Finally, the rate of children who had been sick at least one day during the past month was

For peer review only - http://bmjopen.bmj.com/site/about/guidelines.xhtml

lowest for the second generation (15.6%), with increasing rates towards the third generation (17.4%) and those not exposed to HIV (24.2%) (χ^2 = 8.62, p = .013). When limiting the sample to adolescent mothers (age <=19) only, the same magnitude and directions of findings were obtained (see Appendix 1).

Table 2

Infant (0-68 month) developmental outcomes, according to familial patterns of HIV infection

	Overall (*N*=1015)	Third Generation (*n*= 27)	Second Generation (*n*= 264)	No HIV Exposure (*n*= 724)	p-value
**Mullen Scales of Child Development** *(M, SD)*					
Gross Motor Score*	49.6 (12.7)	52.4(16.1)	47.0 (13.1)[b]	50.4 (12.3)	.002
Visual Reception Score	42.1 (14.2)	43.9 (14.7)	40.7 (14.3)	42.6 (14.2)	.14
Fine Motor Score	43.6 (14.6)	44.3 (16.1)	41.4 (15.2)[b]	44.4 (14.2)	.014
Receptive Language Score	47.4 (13.6)	47.7 (13.8)	46.1 (15.0)	47.8 (13.0)	.21
Expressive Language Score	51.6 (13.5)	51.7 (13.4)	50.7 (14.8)	51.9 (13.0)	.48
Composite Score	93.2 (21.4)	94.6 (23.4)	90.6 (23.0)[b]	94.1 (20.7)	.066
**WHO Disability Score- Any Disability** *(N, %)*	242 (26.7%)	7 (26.9%)	69 (27.7%)	166 (26.2%)	.90
**Any Days Sick** (past month) *(N, %)*	218 (21.8%)	4 (17.4%)	41 (15.6%)	173 (24.2%)	.013

Notes. Group comparisons were conducted using χ^2 tests for categorical variables and univariate analyses of variance for continuous variables. *Gross motor score: n = 831. Mullen subdomain scores range from 20-80, composite scores from 49-155. Tukey's range post hoc tests were undertaken to identify mean differences between groups for continuous variable: [a] Statistically different from the second generation (p<0.05), [b] Statistically different from HIV unexposed group (p<0.05).

Maternal characteristics for the three subgroups

Mothers of the second and third generations did not differ regarding ART adherence, clinic appointments missed, and HIV-related stigma (p= .69-.95). Both groups were about half as likely to have been in excellent physical health in the past twelve months as mothers of non-exposed children (χ^2= 108.46; p <.001). Mental health symptom scores were overall low, and

For peer review only - http://bmjopen.bmj.com/site/about/guidelines.xhtml

no differences were found between groups in terms of anxiety (F= .89, p= .41) and only marginal differences for PTSD, with mothers from the second generation scoring higher than bother other groups (F= 2.38 p = .093). Mothers of the second generation also had the highest depression scores (F= 6.12, p = .002), with post-hoc tests indicating significant differences to those not HIV exposed (p = .005) and marginal differences to mothers of third generation (p = .053). Second generation mothers also scored substantially higher than both other groups on suicidality levels (F= 4.11, p = .017), though due to low power, only significant differences to mothers not living with HIV were identified in post-hoc tests (p = .017). Finally, no group differences were detected for exposure to intimate partner and community violence or parenting stress (p= .39-.97). However, mothers of the third generation indicated the lowest levels of social support, followed by the second generation and then mothers of non-exposed children (F= 3.98, p= .019), though post-hoc tests indicated only close to significant differences between mothers of the third generation and those of HIV-unexposed children (p = .061; see Table 3).

Table 3

Comparison of maternal life circumstances, according to familial patterns of HIV infection

	Overall (N=1015)	Third Generation (n= 27)	Second Generation (n= 264)	No HIV Exposure (n= 724)	p-value
**HIV-associated variables**					
Full ART Adherence past 7 days	213 (75.8%)	19 (79.2%)	194 (75.5%)	-	.69
Any Clinic Appointments Missed past month	10 (4.1%)	1 (4.6%)	9 (4.1%)	-	.92
Any Stigma	56 (20.3%)	5 (20.8%)	51 (20.2%)	-	.95
**Physical and Mental Health**					
Excellent Physical Health past 12 months	598 (58.6%)	9 (33.3%)	88 (33.3%)	499 (68.9%)	<.001

For peer review only - http://bmjopen.bmj.com/site/about/guidelines.xhtml

Depression Score past 2 weeks (0-20)	.7 (1.4)	.3 (.7)	1.0 (1.7)[b]	.6 (1.3)	.002
Anxiety Score past month (0-14)	.8 (1.9)	.7 (1.6)	.6 (1.9)	.8 (1.9)	.41
PTSD Score past month (0-36)	3.0 (5.6)	2.0 (4.9)	3.6 (5.6)	2.9 (4.9)	.09
Suicidality Score past month (0-5)	.2 (.8)	.1 (.3)	.3 (1.1)[b]	.1 (.7)	.017
**Social and Environmental Factors**					
Parenting Stress Score (18-90)	24.8 (5.8)	24.7 (4.8)	24.9 (5.6)	24.8 (5.8)	.97
Social Support Score (0-14)	13.4 (2.04)	12.7 (2.5)	13.2(2.3)	13.5 (1.9)	.019
Extent of Intimate Partner Violence Exposure (0-4)	.1 (.6)	.1 (.4)	.2 (.7)	.1 (.5)	.39
Extent of Community Violence Exposure (0-4)	.3 (.8)	.2 (.6)	.3 (.8)	.3 (.8)	.79

Notes. Group comparisons were conducted using χ^2 tests for categorical variables and univariate analysis of variance for continuous variables. Tukey's range post hoc tests were undertaken to identify mean differences between groups for continuous variable: [a] Statistically different from the second generation (p<0.05), [b] Statistically different from HIV unexposed group (p<0.05). Underlying *N*s vary depending on missing values (N= 950 to N= 1015; N = 828 for Intimate Partner Violence- low response rates may be due to the sensitive topic).

Regression models for predicting child cognitive development composite score

Table 4 shows the results of the hierarchical linear regression model predicting the child composite score on the Mullen Scales. In step 1, compared to the baseline class of children not exposed to HIV, being a child of the second generation was associated with a lower developmental score (p= .021); no predictive effect was found for being a child of the third generation (p = .91; model: $F(2,1012)$ = 2.72, p = .066). The former association was retained at a marginal level in the adjusted model in step 2 (p = .059; overall model: $F(8,889)$ = 4.08, p <.001), with mothers having repeated a school grade additionally being predictive of poorer outcomes, and higher maternal age at birth exerting a protective influence. No predictive effects

For peer review only - http://bmjopen.bmj.com/site/about/guidelines.xhtml

were found for maternal depression, social support, number of necessities, and food insecurity. Overall, however, the amount of variance explained by both models was relatively low.

Table 4

Multiple regression analysis predicting child developmental score from familial patterns of HIV infection in step 1, with step 2 controlling for relevant child and maternal variables

	Unstandardized regression weights (B;95% CI)	Standardized regression weights (β)	p-value
**Step 1: Unadjusted model**			
Mother not living with HIV (Ref.)	-		
Mother perinatally infected	.46 (-7.77; 8.69)	.00	.91
Mother recently infected	-3.5 (-6.57; -.53)	-.07	.021
Adjusted R2	.003		
**Step 2: Adjusted model**			
Mother not living with HIV (Ref.)	-		
Mother perinatally infected	1.06 (-7.51; 9.65)	.01	.81
Mother recently infected	-3.36 (-6.85; .12)	-.07	.059
Maternal Depression Score	-.45 (-1.46; .57)	-.03	.39
Maternal Social Support	.21 (-.47; .89)	.02	.54
Number of Necessities	.36 (-.30; .1.03)	.04	.29
Maternal Age at Pregnancy	1.10 (.21; 1.99)	.09	.016
Mother repeated any school grade	-5.98 (-8.87; -3.07)	-.14	<.001
Number of days insufficient food for child	-.32 (-1.66; 1.01)	-.02	.64
Adjusted R2	.027		

Note. Step 2 (adjusted model) based on *N* = 898, due to missing data on the control variables. B = unstandardized regression weights. β = standardized regression weights.

DISCUSSION

In this sample of young mothers and their infants living in South Africa, we found that children of the second generation (i.e., with mothers recently infected with HIV) scored significantly lower on gross motor and fine motor functioning. For the overall composite score they tended to show lower scores as well than children not exposed to HIV. Children of the third generation (i.e., of perinatally infected mothers) appeared score similarly or even higher

For peer review only - http://bmjopen.bmj.com/site/about/guidelines.xhtml

than children not exposed to HIV across all domains. However, the comparatively small sample size in this group makes it challenging to draw definite conclusions. Mothers also differed in their life circumstances, with mothers of the second generation showing higher rates of depression and suicidality symptoms, as well as lower food security, while mothers of the third generation indicated lower social support. No differences were found on adherence and clinical attendance variables.

Any interpretation of the current data warrants caution, due to low power resulting from the small sample size of the third-generation group. Still, our data appear to provide a tentative first indication that children of the third generation could be doing similarly well as those not exposed to familial HIV across most developmental domains. This would be promising and may indicate that families affected by HIV are in the long-term profiting from efforts to provide social protection, links to services and antiretroviral treatment, which could prevent chronic adversity from being perpetuated. However, it remains to be seen whether such benefits are sustained, with studies in children living with HIV suggesting positive early developmental outcomes, but declines with age [26; Roberts et al., in preparation]. While the aim is to ultimately prevent perinatal HIV infections, larger studies of already infected young adults and their children may provide further insights into the development and needs of children of the third generation.

Children of the second generation, on the other hand, tended to do poorer across several domains, an effect that was marginally retained when controlling for maternal mental health, social support, and socio-demographic factors. The shock of maternal recent HIV diagnosis may have placed a major burden on these families, compounded by risk drivers which may have accounted for the HIV exposure in the first place, such as economic and food insecurity, or mental health struggles. This indicates that affected families may profit from additional support, including early cognitive interventions for infants [10, 42] and emotional and social

For peer review only - http://bmjopen.bmj.com/site/about/guidelines.xhtml

support for mothers at the time of and following diagnosis. Furthermore, these mothers may need to be fast-tracked into services, given the particular double vulnerability of HIV and teen pregnancy.

Of note, the overall child sample underperformed somewhat compared to the American normative sample [23] and a similar group of children in Zimbabwe, who had caregivers living with HIV.[43] Although the level of disability did not differ across the three groups, 26% of the total sample scored for any disability. Targeted interventions could improve developmental outcomes,[44] and adapted disability services may be needed for many of these young children. The results from the current study indicate that addressing factors such as young pregnancy or poor maternal education could be of particular benefit. Maternal school progression delay in particular has been shown to be a risk factor for both early motherhood and HIV infection, as well as an outcome of perinatal HIV infection.[45, 46, 47] Therefore, it will be important to study this factor as a risk marker for child poor developmental outcomes. However, it is important to note that our model only explained a low amount of variance overall, suggesting that other factors may be key for improving child developmental outcomes.

It is important to note though that all adolescent mothers and their children in the current sample had a number of challenges to contend with. Only 24.6% attended creche (no difference by group). The mean age for first childbirth was 16.76 years, with the teens living with HIV being on average about a year older. Over 90% were in receipt of some form of household grant. This highlights the compounded disadvantages that adolescent/young mothers living in marginalized areas may face, and the urgent need for support and intervention [22]. In accordance with previous literature, [16] adolescents faced different challenges depending on whether they were perinatally or recently infected (lower social support in the third generation, versus food insecurity and mental-health related difficulties in the second generation). This suggests second generation mothers may require additional support in the form of social

For peer review only - http://bmjopen.bmj.com/site/about/guidelines.xhtml

protection and access to services [48, 49]. Mothers of the third generation, on the other hand, appear to have adapted to some degree, but may profit from initiatives to reduce chronic stigma and improve social support in an environment that has been long-term affected by HIV [50].

The current study has several limitations. Firstly, the data collected are cross-sectional in nature, and thus do not allow any deductions about temporal relations or causality. Furthermore, they stem solely from South Africa and sampling focused predominantly on a particular group (adolescent mothers from disadvantaged environments) and was thus not completely random, potentially limiting the generalizability of our findings to other countries and contexts. Thirdly, the majority of maternal data were collected through self-report, allowing the possibility for bias. Particularly maternal mental health difficulty rates were overall very low in this sample, leading to the question of whether this is due to high resilience or measurement difficulties. Fourth, as the study was community-based, and clinic records were wanting, an algorithm was required to categorise perinatal infection. Fifth, the standardised developmental inventory has been validated and used widely in Sub-Africa (e.g., Zimbabwe, Zambia, South Africa), but is set against USA norms, and local inventories with local references groups may be more sensitive for future studies. Sixth, while our sample predominantly comprised adolescent mothers, some older mothers up to age 25 whom we had historical data for were also included in our analyses to increase analytical power. This limited our ability to draw conclusions for adolescent mothers specifically. Furthermore, our study may not generalise to older mothers and data on fathers could help to further enhance our understanding. Yet it seems important that real life multiple challenges (i.e., compounded effects of HIV exposure and young motherhood) should be appreciated, and these insights applied to wider groups. Finally, the group of children of the third generation was relatively small, which -while encouraging giving HIV preventative efforts- limited our power to investigate group differences and to perform more complex analyses, such as mediation or path

For peer review only - http://bmjopen.bmj.com/site/about/guidelines.xhtml

models, to better understand pathways for intervention. Future studies may aim at specifically recruiting a larger sample of third generation children to replicate and extend our findings. The effects of child HIV on cognitive outcomes in the overall sample are explored elsewhere (Roberts et al., in preparation).

CONCLUSION

The current paper takes an important first look at the children of the “third generation”, giving an impression of their developmental status and life circumstances. The data tentatively suggests that young mothers who have lived in families with HIV for many years learn to understand and accommodate the situation, have worked out pathways to support, and may not have to deal with the shock of HIV diagnosis at the time of pregnancy. Newly infected adolescents may not have the same level of family support and understanding, and they may be coming to terms with their diagnosis at the same time as adjusting to motherhood. For all children, access to early childhood programmes was low, despite them having a good evidence base for enhancing child outcomes. Policies around such provisions should be incorporated for these groups. Future studies will benefit from longitudinal data, including a larger group of children of mothers perinatally infected with HIV, to better understand child cognitive development of the second and third generation, and how this is influenced by maternal factors. While the long-term aim will be to eradicate perinatal and later life HIV infections completely, it is important to further understand how to support affected families adequately.

Contributorship Statement: LS, ET, & LC conceptualised the overall study. ET, LC, CW, BS, NL, JJ, JC, MM and WS were involved in study design, data collection and/or data management. KH led and KR, JT, WS, and SZ contributed to the current analyses. LS and KH were predominantly responsible for the writing of the paper, with contributions from the rest of the team. Two adolescent advisory groups served as advisors for contents and acceptability of the questionnaires and recruitment strategies. Community leaders served as advisors for recruitment.

For peer review only - http://bmjopen.bmj.com/site/about/guidelines.xhtml

Funding: This study received funding from the European Research Council (ERC) under the European Union's Horizon 2020 research and innovation programme (grant agreement No 771468); the United States Agency for International Development (USAID) Cooperative Agreement No. AID-OAA-LA-13-00001 and was made possible by the generous support of the American people through USAID and the United States President's Emergency Plan for AIDS Relief. The contents are the responsibility of Oxford University and do not necessarily reflect the views of USAID or the United States Government; Research England [0005218]; jointly from the UK Medical Research Council (MRC) and the UK Department for International Development (DFID) under the MRC/DFID Concordat agreement, and by the Department of Health Social Care (DHSC) through its National Institutes of Health Research (NIHR) [MR/R022372/1]; the Leverhulme Trust [PLP-2014-095]; UCL's HelpAge funding; UNICEF Eastern and Southern Africa Office (UNICEF-ESARO); from a CIPHER grant from International AIDS Society [2018/625-TOS]. The views expressed in written materials or publications [OR presented in this presentation] do not necessarily reflect the official policies of the International AIDS society; UKRI GCRF Accelerating Achievement for Africa's Adolescents (Accelerate) Hub (Grant Ref: ES/S008101/1); the Fogarty International Center, National Institute on Mental Health, National Institutes of Health under Award Number K43TW011434. The content is solely the responsibility of the authors and does not represent the official views of the National Institutes of Health; Oak Foundation [grant number: OFIL-20-057]; the Economic and Social Research Council [grant number ES/R501037/1]; the Economic and Social Research Council (grant number ES/J500112/1) the Grand Union - Doctoral Training Partnership; an Economic Social Research Council (ESRC) PhD studentship through the UBEL DTP.

Conflicts of Interest: None to declare

Ethical Approval Statement: Ethical approvals were obtained from the Universities of Oxford (R48876/RE002) and Cape Town (HREC 226/2017) as well as University College London for secondary data analysis (14795/001). Additional local approvals and permissions were obtained from the Provincial Departments (Eastern Cape, South Africa) of Health, Education, and Social Development. The current research conforms to the principles embodied in the Declaration of Helsinki. Where need was identified among the study population (e.g., mental health needs), appropriate referrals to local services were made.

Data Sharing Agreement: The dataset is available upon request.

For peer review only

Acknowledgements: The authors are grateful to the young mothers and their families, the tireless data collection team, and partner organizations who supported the research process. We also thank our funders, including FHI360, the European Research Council, United States Agency for International Development, USAID, United States President's Emergency Plan for AIDS Relief, Research England, UK Medical Council, UK Department for International Development, National Institute of Health Research, Leverhulme Trust, University College London HelpAge, UNICED Eastern and Southern Africa Office, International AIDS Society, International AIDS society, UKRI GCRF Accelerating Achievement for Africa's Adolescents Hub, Fogarty International Center, National Institute on Mental Health, National Institutes of Health, Oak Foundation and the Economic and Social Research Council.

For peer review only

REFERENCES

[1] UNAIDS (2020). Infographic- people living with HIV. https://www.unaids.org/sites/default/files/media_asset/2020_aids-data-book_en.pdf [Date Accessed: 14 May 2021]

[2] LeCroix RH, Chan WY, Henrich C et al. Maternal HIV and adolescent Functioning in South Africa: The role of the mother-child relationship. *The Journal of Early Adolescence*. 2020;40(1):83103.doi:10.1177/0272431618824726 [published Online First: 24 January 2019].

For peer review only - http://bmjopen.bmj.com/site/about/guidelines.xhtml

[3] Richter LM, Sherr L, Adato M, et al. Strengthening families to support children affected by HIV and AIDS. *AIDS care*. 2009;21(sup1):3-12.doi: 10.1080/09540120902923121 [published Online First: 30 September 2009]
[4] Lowenthal ED, Bakeera-Kitaka S, Marukutira et al. Perinatally acquired HIV infection in adolescents from sub-Saharan Africa: a review of emerging challenges. *The Lancet infectious diseases*. 2014;14(7):627-39.doi:10.1016/S1473-3099(13)70363-3 [published Online First: 7 January 2014].
[5] South African National Department of Health. National Consolidated Guidelines for the Prevention of Mother-To-Child Transmission of HIV (PMTCT) and the Management of HIV in Children, Adolescents and Adults. https://sahivsoc.org/Files/ART%20Guidelines%2015052015.pdf [Date Accessed: 10 September 2021].
[6] Mann JM. A worldwide pandemic. In Gottlieb MS et al., eds. Current Topics in AIDS - Volume 2. Wiley and Sons.
[7] Banks LM, Zuurmond M, Ferrand R, Kuper H. The relationship between HIV and prevalence of disabilities in sub-Saharan Africa: systematic review (FA). *Tropical medicine & international health*. 2015; 20(4):411-29.doi: 10.1111/tmi.12449 [published Online First: 12 December 2014].
[8] McHenry MS, McAteer CI, Oyungu E et al. Neurodevelopment in young children born to HIV-infected mothers: a meta-analysis. *Pediatrics*. 2018;141(2). doi:10.1542/peds.2017-2888 [published Online First: 1 February 2018].
[9] Sherr L, Croome N, Castaneda KP et al. Developmental challenges in HIV infected children—An updated systematic review. *Children and Youth Services Review*. 2014;45:74-89.doi: 10.1016/j.childyouth.2014.03.040 [published Online First: 4 April 2014].
[10] Sherr L, Croome N, Bradshaw K, Parra Castaneda K. A systematic review examining whether interventions are effective in reducing cognitive delay in children infected and affected with HIV. *Aids Care*. 2014;26(sup1):S70-7.doi: 10.1080/09540121.2014.906560 [first Published Online: 10 April 2014].
[11] Townsend C, Schulte J, Thorne C et al. Antiretroviral therapy and preterm delivery-a pooled analysis of data from the United States and Europe. *BJOG : an international journal of obstetrics and gynaecology*.2010; 117(11):1399–1410. doi:10.1111/j.1471-0528.2010.02689.x
[12] Cruz ML, Cardoso CA, Joao EC et al. Pregnancy in HIV vertically infected adolescents and young women: a new generation of HIV-exposed infants. Aids. 2010;24(17):2727-31. doi: 10.1097/QAD.0b013e32833e50d4 [published Online First: 13 November 2010].
[13] Allen AB, Finestone M, Eloff I et al. The role of parenting in affecting the behavior and adaptive functioning of young children of HIV-infected mothers in South Africa. *AIDS and Behavior.* 2014;18(3):605-16.doi:10.1007/s10461-013-0544-7 [published Online First: 28 July 2013].
[14] Bailey H, Cruz ML, Songtaweesin WN, Puthanakit T. Adolescents with HIV and transition to adult care in the Caribbean, Central America and South America, Eastern Europe and Asia and Pacific regions. *Journal of the International AIDS Society.* 2017;20:21475.doi:10.7448/IAS.20.4.21475 [published Online First: 16 May 2017].
[15] Earnshaw VA, Kidman RC, Violari A. Stigma, depression, and substance use problems among perinatally HIV-infected youth in South Africa. *AIDS and Behavior*. 2018; 22(12):3892-6.doi: 0.1007/s10461-018-2201-7 [published Online First: 16 June 2018].

For peer review only - http://bmjopen.bmj.com/site/about/guidelines.xhtml

[16] Rochat T, Netsi E, Redinger S, Stein A. Parenting and HIV. *Current opinion in psychology*. 2017;15:155-61.doi: 10.1016/j.copsyc.2017.02.019 [published Online First: 22 February 2017].
[17] Sherr L, Cluver LD, Toska E, He E. Differing psychological vulnerabilities among behaviourally and perinatally HIV infected adolescents in South Africa–implications for targeted health service provision. *AIDS care*. 2018;30(sup2):92-101. 10.1080/09540121.2018.1476664 [published Online First: 30 May 2018].
[18] Anderson M, Elam G, Gerver S, et al. "It took a piece of me": Initial responses to a positive HIV diagnosis by Caribbean people in the UK. *AIDS care*. 2010;22(12):1493-8. doi: 10.1080/09540121.2010.482125 [published Online First: 6 December 2018].
[19] Ramirez-Avila L, Regan S, Giddy J, et al. Depressive symptoms and their impact on health-seeking behaviors in newly-diagnosed HIV-infected patients in Durban, South Africa. *AIDS and Behavior*. 2012;16(8):2226-35.doi: 10.1007/s10461-012-0160-y [published Online First: 27 March 2012].
[20] Yah CS, Ndlovu S, Kutywayo A, et al. The prevalence of pregnancy among adolescent girls and young women across the Southern African development community economic hub: A systematic review and meta-analysis. *Health promotion perspectives*. 2020;10(4):325. doi:10.34172/hpp.2020.51 [published Online First: 7 November 2020].
[21] Glassman AL, Silverman R, McQueston K. Adolescent fertility in low-and middle-income countries: effects and solutions. Center for Global Development Working Paper. 2012;295.Available at SSRN: https://ssrn.com/abstract=2102763 or http://dx.doi.org/10.2139/ssrn.2102763 [Date Accessed: 10 September 2021]
[22] Toska E, Laurenzi CA, Roberts KJ et al. Adolescent mothers affected by HIV and their children: a scoping review of evidence and experiences from sub-Saharan Africa. *Global Public Health*. 2020;15(11):1655-73.doi: 10.1080/17441692.2020.1775867 [published Online First: 6 June 2020].
[23] Mullen EM. Mullen scales of early learning. Circle Pines, MN: AGS 1995:58-64.
[24] Bornman J, Romski M, Tonsing K, et al. Adapting and translating the Mullen Scales of Early Learning for the South African context. *South African Journal of Communication Disorders*. 2018;65(1):1-9.doi: 10.4102/sajcd.v65i1.571[published Online First: 8 March 2018].
[25] Koura GK, Boivin MJ, Davidson LL et al. Adaptation of the mullen scales of early learning to Benin setting. *American Journal of Epidemiology*. 2012;175(11):S128-.
[26] Milosavljevic B, Vellekoop P, Maris H, et al. Adaptation of the Mullen Scales of Early Learning for use among infants aged 5-to 24-months in rural Gambia. Developmental science. 2019;22(5):e12808. doi:10.1111/desc.12808 [published Online First: 9 February 2019].
[27] Durkin MS, Wang W, Shrout PE et al. Evaluating a Ten Questions screen for childhood disability: reliability and internal structure in different cultures. *Journal of Clinical Epidemiology*, 1995;48(5):657-66. doi:10.1016/0895-4356(94)00163-k
[28] Pillay U, Roberts B, Rule S. South African social attitudes: changing times, diverse voices. Cape Town: HSRC press 2006.
[29] Statistics South Africa. *Census 2001: Household Questionnaire*. Pretoria, SA: Statistics SA 2001.
[30] Labadarios D, Steyn NP, Maunder E et al. The national food consumption survey (NFCS): South Africa, 1999. *Public health nutrition*. 2005;8(5):533-43.doi:10.1079/phn2005816 [published Online First: 2 January 2007].

For peer review only - http://bmjopen.bmj.com/site/about/guidelines.xhtml

[31] Duong M, Piroth L, Grappin M et al. Evaluation of the Patient Medication Adherence Questionnaire as a tool for self-reported adherence assessment in HIV-infected patients on antiretroviral regimens. *HIV clinical trials*. 2001;2(2):128-35.doi: 10.1310/M3JR-G390-LXCM-F62G [published Online First: 29 January 2015].
[32] Pantelic M, Boyes M, Cluver L, Thabeng M. 'They say HIV is a punishment from god or from ancestors': cross-cultural adaptation and psychometric assessment of an HIV stigma scale for South African adolescents living with HIV (ALHIV-SS). *Child indicators research*. 2018;11(1):207-23. doi:10.1007/s12187-016-9428-5 [published Online First: 23 November 2016].
[33] Kovacs M. Children's depression inventory (CDI). Toronto: Multi-Health System 2003.
[34] Reynolds CR, & Richmond BO. Revised Children's Manifest Anxiety Scales (RCMAS). Los Angeles: Western Psychological Services 1985.
[35] Amaya-Jackson L, McCarthy G, Cherney MS & Newman E. Child PTSD checklist. Durham, NC: Duke University Medical Center 1995.
[36] Sheehan DV, Lecrubier Y, Sheehan KH et al. The Mini-International Neuropsychiatric Interview (MINI): the development and validation of a structured diagnostic psychiatric interview for DSM-IV and ICD-10. *Journal of clinical psychiatry*. 1998;59(20):22-33.
[37] Martin L, Revington N, Seedat S. The 39-item Child Exposure to Community Violence (CECV) Scale: Exploratory factor analysis and relationship to PTSD symptomatology in trauma-exposed children and adolescents. *International journal of behavioral medicine.* 2013;20(4):599-608.doi: 10.1007/s12529-012-9269-7 [published Online First: 2 October 2012].
[38] Finkelhor D, Hamby SL, Ormrod R, Turner H. The Juvenile Victimization Questionnaire: reliability, validity, and national norms. *Child abuse & neglect*. 2005;29(4):383-412.doi: 10.1016/j.chiabu.2004.11.001 [published Online First: 28 April 2005].
[39] Berry JO, Jones WH. The parental stress scale: Initial psychometric evidence. *Journal of social and personal relationships*. 1995 Aug;12(3):463-72.doi: 10.1177/0265407595123009
[40] Sherbourne CD, Stewart AL. The MOS social support survey. *Social science & medicine*. 1991;32(6):705-14.doi: 10.1016/0277-9536(91)90150-B
[41] Casale M. The importance of family and community support for the health of HIV-affected populations in Southern Africa: What do we know and where to from here?. *British Journal of Health Psychology*. 2015;20(1):21-35.doi: 10.1111/bjhp.12127 [first Published Online: 9 December 2014].
[42] Thomas T, Ahmed Y, Tan M, Grigorenko EL. Cognitive and Educational Interventions for Orphans and Vulnerable Children Affected by HIV/AIDS: A Meta-Analysis. *Child development*. 2021;92(1):7-20. DOI: 10.1111/cdev.13438
[43] Mebrahtu H, Simms V, Mupambireyi Z et al. Effects of parenting classes and economic strengthening for caregivers on the cognition of HIV-exposed infants: a pragmatic cluster randomised controlled trial in rural Zimbabwe. *BMJ global health*. 2019;4(5):e001651.doi: 10.1136/bmjgh-2019-001651[published Online First: 11 September 2019].
[44] Rao N, Sun J, Wong JM et al. Early childhood development and cognitive development in developing countries: A rigorous literature review. http://cerc.edu.hku.hk/wp-content/uploads/ECD-review.pdf [Date accessed: 10 May 2021]
[45] Johnson LF, Dorrington RE, Bradshaw D et al. The effect of educational attainment and other factors on HIV risk in South African women: results from antenatal

For peer review only - http://bmjopen.bmj.com/site/about/guidelines.xhtml

surveillance, 2000–2005. *Aids*. 2009;23(12):1583-8. doi: 10.1097/QAD.0b013e32832d407e [published Online First: 31 July 2009].
[46] Merville O, Puangmala P, Suksawas P et al. School trajectory disruption among adolescents living with perinatal HIV receiving antiretroviral treatments: a case-control study in Thailand. *BMC public health*. 2021;21(1):1-2.doi: 10.1186/s12889-021-10189-x [published Online First: 21 January 2021].
[47] Toska E, Cluver L, Orkin M, Bains A, Sherr L, Berezin M, Gulaid L. Screening and supporting through schools: Educational experiences and needs of adolescents living with HIV in a South African cohort. *BMC Public Health*. 2019;19(1):1-0. doi: 10.1186/s12889-019-6580-0 [published Online First: 6 March 2019].
[48] Toska E, Gittings L, Hodes R, et al. Resourcing resilience: social protection for HIV prevention amongst children and adolescents in Eastern and Southern Africa [published correction appears in Afr J AIDS Res. 2016 Sep;15(3):314]. *Afr J AIDS Res*. 2016;15(2):123-140. doi:10.2989/16085906.2016.1194299
[49] van der Wal R, Loutfi D, Hong QN, Vedel I, Cockcroft A, Johri M, Andersson N. HIV-sensitive social protection for vulnerable young women in East and Southern Africa: a systematic review. J Int AIDS Soc. 2021 Sep;24(9):e25787. doi: 10.1002/jia2.25787. PMID: 34473406; PMCID: PMC8412122.
[50] Pantelic M, Steinert JI, Park J, Mellors S, Murau F. 'Management of a spoiled identity': systematic review of interventions to address self-stigma among people living with and affected by HIV. BMJ Glob Health. 2019*; 4*(2):e001285. doi:10.1136/bmjgh-2018-00128

For peer review only

Figure Legend

Figure 1

Flow Chart of Classification by Mode of Maternal HIV Acquisition

For peer review only - http://bmjopen.bmj.com/site/about/guidelines.xhtml

For peer review only

For peer review only - http://bmjopen.bmj.com/site/about/guidelines.xhtml

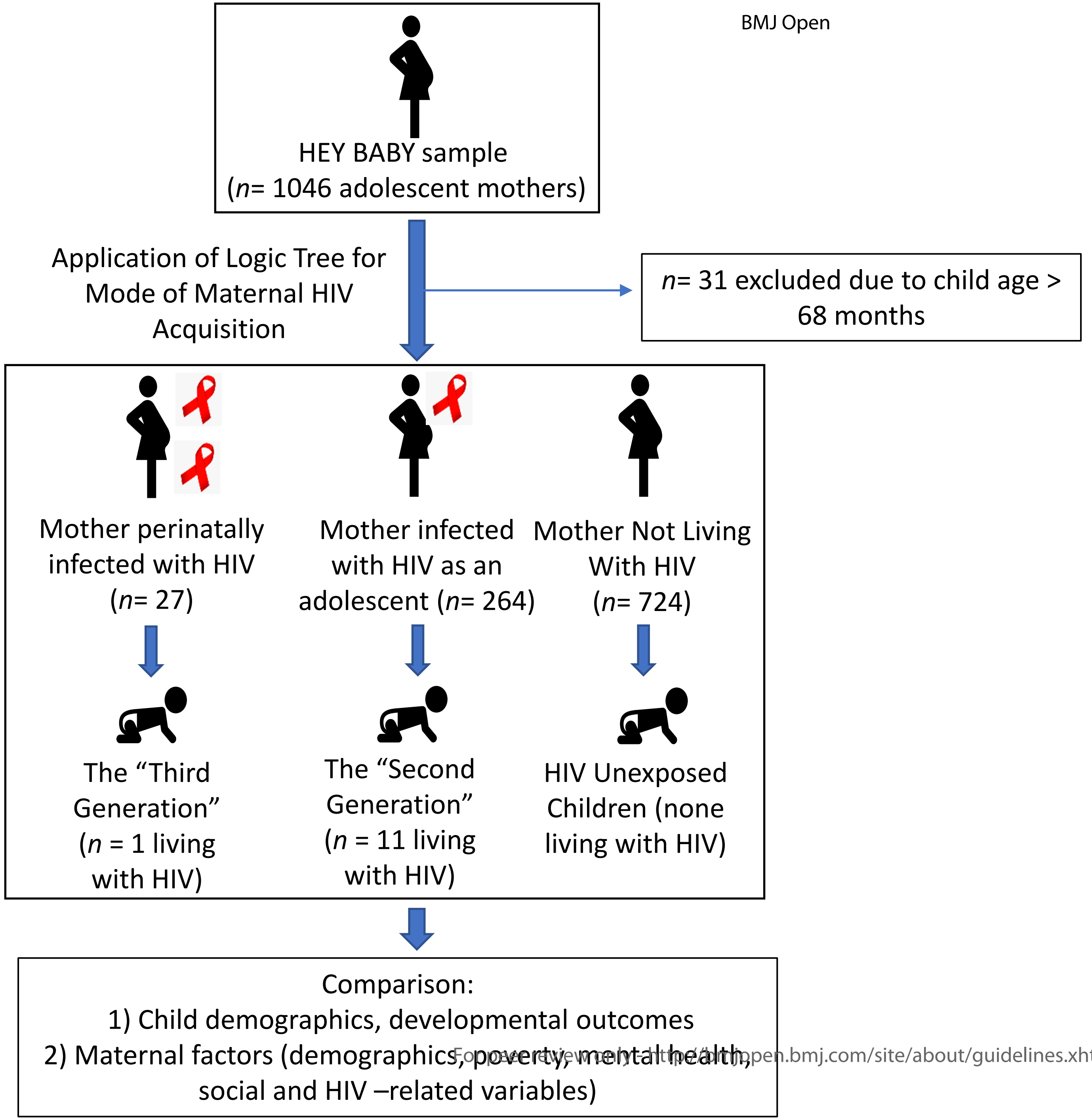

For peer review only - http://bmjopen.bmj.com/site/about/guidelines.xhtml

Appendix 1

Infant (0-68 month) developmental outcomes, according to familial patterns of HIV infection – sample limited to adolescent mothers only

	Overall (*N*=1015)	Third Generation (*n*= 23)	Second Generation (*n*= 229)	No HIV Exposure (*n*= 721)	p-value
**Mullen Scales of Child Development** *(M, SD)*					
Gross Motor Score*	49.7 (12.5)	54.3 (16. 7)[a]	46.8 (12.7)[b]	50.3 (12.2)	<.001
Visual Reception Score	42.1 (14.2)	44.0 (13.7)	40.4 (14.1)	42.5 (14.2)	.12
Fine Motor Score	43.7(14.7)	45.0 (16.2)	41.3 (15.5)[b]	44.4 (14.3)	.017
Receptive Language Score	47.5 (13.5)	48.5 (14.0)	46.4 (14.9)	47.8 (13.0)	.37
Expressive Language Score	51.6 (13.5)	51.1 (14.0)	51.0 (14.8)	51.8 (13.0)	.71
Composite Score	93.3 (21.3)	95.0 (23.1)	90.6 (22.8)	94.1 (20.7)	.098
**WHO Disability Score- Any Disability** *(N, %)*	234 (26.9%)	6 (27.3%)	63 (28.9%)	165 (26.2%)	.73
**Any Days Sick** (past month) *(N, %)*	212 (22.1%)	3 (15.0%)	37 (16.2%)	172 (24.2%)	.032

Notes. Group comparisons were conducted using χ^2 tests for categorical variables and univariate analyses of variance for continuous variables. *Gross motor score: n = 831. Mullen subdomain scores range from 20-80, composite scores from 49-155. Tukey's range post hoc tests were undertaken to identify mean differences between groups for continuous variable: [a] Statistically different from the second generation (p<0.05), [b] Statistically different from HIV unexposed group (p<0.05).

For peer review only
